# 3-*O*-p-coumaroylquinic acid and 4-*O*-p-coumaroylquinic acid from *Hemerocallis citrina* Baroni exert antidepressant effects via CA/Dopa/DA/NE and PKA/CREB/BDNF signaling pathways

**DOI:** 10.1038/s41538-026-00934-0

**Published:** 2026-07-01

**Authors:** Faying Jiang, Zhengyue Qiu, Juhua Zhong, Tian Ye, Jinghong Liu, Yishuo Zhu, Lewei Zhao, Siyu Chen, Hongbao Chen, Haobo Wang, Lin Kang, Tonghe Yang, Shuai Li, Si Liu, Jianguo Zeng, Zhixing Qing

**Affiliations:** 1 College of Veterinary Medicine, Hunan Agricultural University, Changsha, China; 2 College of tea culture, Hunan Vocational College of Commerce, Changsha, China; 3 Yuelushan Laboratory, Changsha, China; 4 Changsha Changxu Biotechnology Ltd., Changsha, China

**Keywords:** Diseases, Drug discovery, Neuroscience

## Abstract

The ancient text “Compendium of Materia Medica” records that *Hemerocallis citrina* Baroni (daylily) was used for antidepressant, but its active components and mechanisms remain unknown. In this study, the *H. citrina* extract was initially fractionated into three parts, and only the 35% HCW (35% ethanol-eluted fraction of *Hemerocallis citrina* water extract, LILYANTIDEPRES^®^) demonstrated significant antidepressant activity, notably increasing the levels of dopamine (DA), norepinephrine (NE), and brain-derived neurotrophic factor (BDNF) in the brain of depressed mice. It’s also significantly reducing the IL-6 (Interleukin-6) and MDA (Malondialdehyde) levels, and improving the gut microbiota. The main components of the 35% HCW were identified as 3-*O-*p-coumaroylquinic acid (**1**) and 4-*O-*p-coumaroylquinic acid (**2**), which were also chemically synthesized. Both compounds exhibited significant antidepressant activity by increasing the levels of DA, NE, and BDNF in the brain of depressed mice and significantly reducing the levels of IL-6 and TNF-α (Tumor Necrosis Factor-α). A potential mechanism to elevate DA and NE levels in the depressed mice brain via the CA (coumaroylquinic acid)/Dopa/DA/NE pathway. Additionally, compounds **1** and **2** significantly increase BDNF levels through the PKA (Protein Kinase A)/CREB (cAMP Response Element-Binding Protein)/BDNF signaling pathway. This study provides the first elucidation of the antidepressant components of *H. citrina* and the mechanisms, laying the foundation for the development of antidepressant products and the high-value utilization of *H. citrina* resources.

## Introduction

Depression is recognized as a major disease of the 21st century, characterized by high prevalence, suicide, disability, and recurrence rates^1,2^. According to the World Health Organization, depression is projected to be the disease with the heaviest economic burden worldwide by 2030^3^. Clinical drug treatment mainly relies on chemically synthesized medications, such as the selective serotonin reuptake inhibitors (SSRIs) fluoxetine and paroxetine, as well as ketamine, which was approved by the U.S. FDA in 2019 for rapidly treating major depressive disorder. However, these synthetic drugs often suffer from limitations such as adverse effects, slow onset of action, low efficacy rates, and high costs, resulting in suboptimal treatment outcomes^4–6^. Consequently, the development of natural antidepressants has become a trend in the research of novel antidepressant drugs.

*Hemerocallis citrina* Baroni, also known as the daylily, is traditionally referred to as the “forget-sorrow herb,” or “calming vegetable”. It belongs to the *Hemerocallis* genus in the Asphodelaceae family and is a plant with a long history of both medicinal and culinary use in China^7,8^. As early as the Ming Dynasty, Li Shizhen’s “Compendium of Materia Medica” recorded that *H. citrina* makes one happy and free from worry, explicitly highlighting its calming and depression-relieving properties^9^. Additionally, *H. citrina* appears as an important herb in various formulas designed to alleviate melancholy and promote peaceful sleep, such as “Xuan Cao Forget-Sorrow Decoction”. Thus, both classical Chinese medical texts and traditional formulas for treating depression indicate that *H. citrina* possesses antidepressant effects.

Scientists have confirmed that *H. citrina* extracts exhibit significant antidepressant activity. In 2012, researchers first discovered that a 75% hydroethanolic extract of *H. citrina* significantly increased the forced swimming time in depressed mice and extended their activity duration in the tail suspension test, thereby demonstrating its antidepressant activity^10,11^. From 2012 to 2023^12–16^, numerous studies have found that *H. citrina* extracts possess notable antidepressant activity, which is closely related to their total phenolic content, however, the specific antidepressant components and mechanisms remain unknown.

The monoamine neurotransmitters such as DA, NE, and 5-hydroxytryptamine (5-HT) are related to the depression^17–19^. In previous studies, researchers investigated the antidepressant activity of *H. citrina* extract, and they found that this activity is closely associated with increased levels of monoamine neurotransmitters like DA and NE in the brains of depressed mice^12,16^. Our earlier studies also demonstrated that *H. citrina* extract can significantly elevate the concentrations of monoamine neurotransmitters, including DA and NE, in the frontal cortex and hippocampal region of depressed mice^12^.

The PKA/CREB/BDNF pathway is also closely associated with neuronal survival and antidepressant responses. Studies have indicated that after the activation of the PKA/CREB/BDNF signaling pathway, it can exert a certain protective effect on cortisol-induced hippocampal neuron damage and improve depressive conditions^20,21^. The binding of BDNF to TrkB (Tropomyosin receptor kinase B) can, in turn, activate CREB, promoting the growth and differentiation of neurons and alleviating depressive symptoms^22^. Research has also confirmed that after the onset of depression, the expressions of PKA, CREB, and BDNF in the brain are inhibited, while antidepressant treatment can activate the activities of PKA, CREB, and BDNF^23–25^. Our previous research indicated that *H. citrina* extract can significantly increase the levels of BDNF in the cerebral cortex and hippocampal region of depressed mice^12^.

Accumulating evidence suggests that depression is closely associated with alterations in gut microbiota, which may affect brain function through the gut–brain axis^26–29^. Chronic stress may disrupt gut microbial homeostasis, increase endotoxin levels such as LPS, induce peripheral inflammatory responses, and promote the release of pro-inflammatory cytokines, including IL-6 and TNF-α. These circulating inflammatory mediators may further activate microglia in the cerebral cortex and hippocampus, leading to neuroinflammation and neuronal damage, thereby contributing to the development of depression^30,31^. Whether the active compounds in *H. citrina* exert antidepressant effects through these pathways remains unclear.

**Fig. 1:**
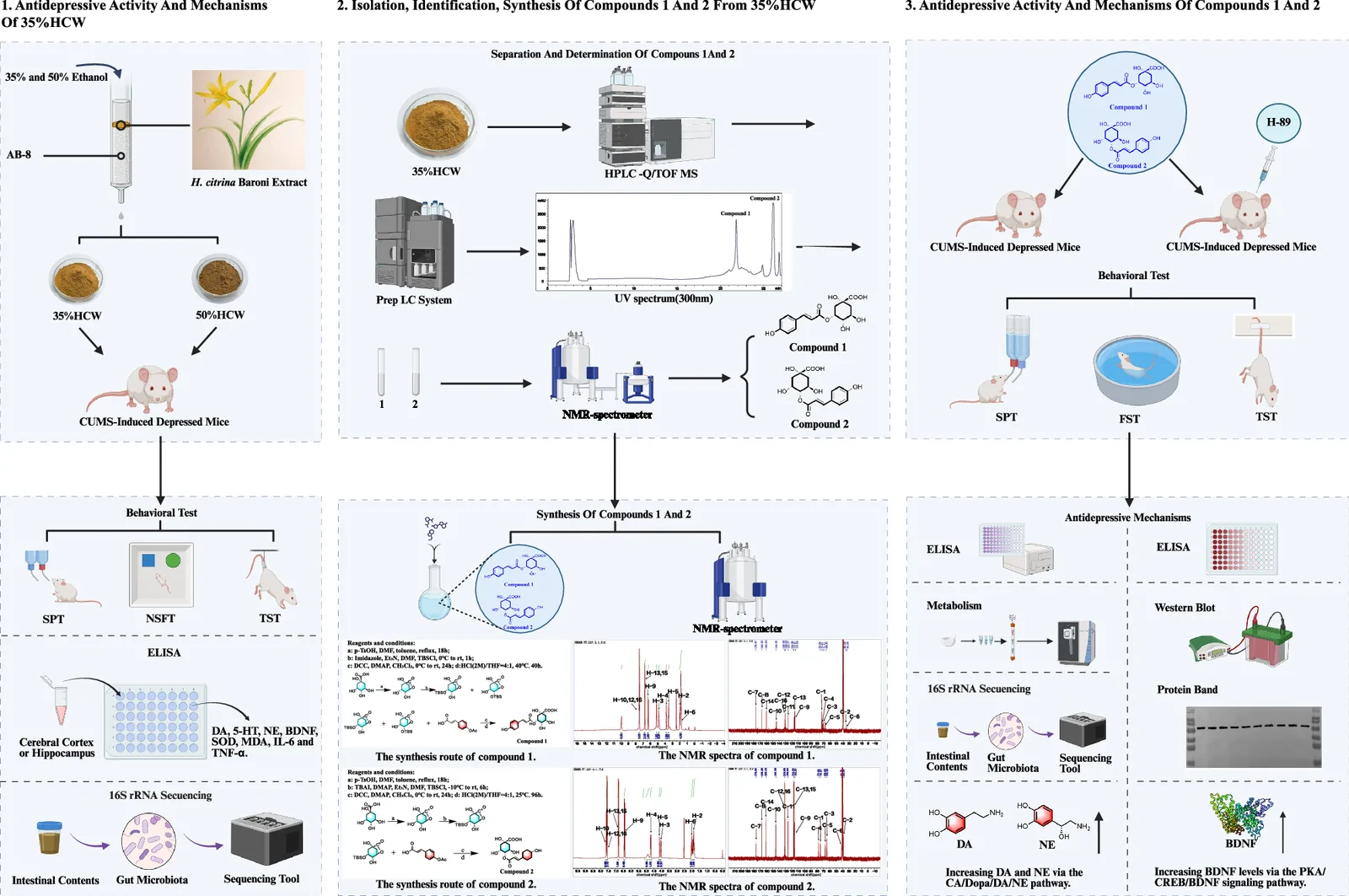
Flowchart of the whole experiment. Created in BioRender. Jiang, F. (2026) https://BioRender.com/8tmz93b. (35% HCW 35% ethanol-eluted fraction of *Hemerocallis citrina* water extract, 50% HCW 50% ethanol-eluted fraction of *Hemerocallis citrina* water extract, 5-HT 5-Hydroxytryptamine, AB-8 AB-8 macroporous adsorption resin, BDNF Brain-Derived Neurotrophic Factor, CA Coumaroylquinic Acid, CREB cAMP Response Element-Binding Protein, CUMS Chronic Unpredictable Mild Stress, DA Dopamine, ELISA Enzyme-Linked Immunosorbent Assay, FST Forced Swim Test, HPLC-Q/TOF MS High Performance Liquid Chromatography-Quadrupole/Time of Flight Mass Spectrometry, IL-6 Interleukin-6, ILT Ingestion Latency Test, MDA Malondialdehyde, NE Norepinephrine, NMR Nuclear Magnetic Resonance, PKA Protein Kinase A, SOD Superoxide Dismutase, SPT Sucrose Preference Test, TNF-α Tumor Necrosis Factor-α, TST Tail Suspension Test, UV Ultraviolet-Visible.

In this study, we first conducted an experiment on the antidepressant activities of different fractions (35% and 50% HCW) of the water extract from blooming *H. citrina* Baroni, and 35% HCW display significant antidepressant activity. Subsequently, the main components of 35% HCW (3-*O*-p-coumaroylquinic acid (**1**) and 4-*O*-p-coumaroylquinic acid (**2**)) were isolated, identified, and synthesized. The antidepressant activity and mechanisms of 3-*O*-p-coumaroylquinic acid and 4-*O*-p-coumaroylquinic acid were further studied via the CA/Dopa/DA/NE and PKA/CREB/BDNF signaling pathways, inflammatory, and gut microbiota theory. The flowchart of the whole experiment see Fig. 1.

## Results

### The 35% HCW exhibits significant antidepressant activity

One-way ANOVA showed no significant overall group effect on sucrose preference, F(12, 117) = 1.74, *p* = 0.067. One-way ANOVA showed a significant overall group effect on feeding latency, F(12, 117) = 4.91, *p* < 0.0001. Dunnett’s multiple comparisons test showed that the 35% HCW-H group had a significantly shorter feeding latency than the MOD group (*p* < 0.01). One-way ANOVA showed no significant overall group effect on the activity time in the tail suspension test, F(12, 117) = 1.64, *p* = 0.091. One-way ANOVA showed no significant overall group effect on immobility time in the tail suspension test, F(12, 117) = 1.69, *p* = 0.078.

**Fig. 2:**
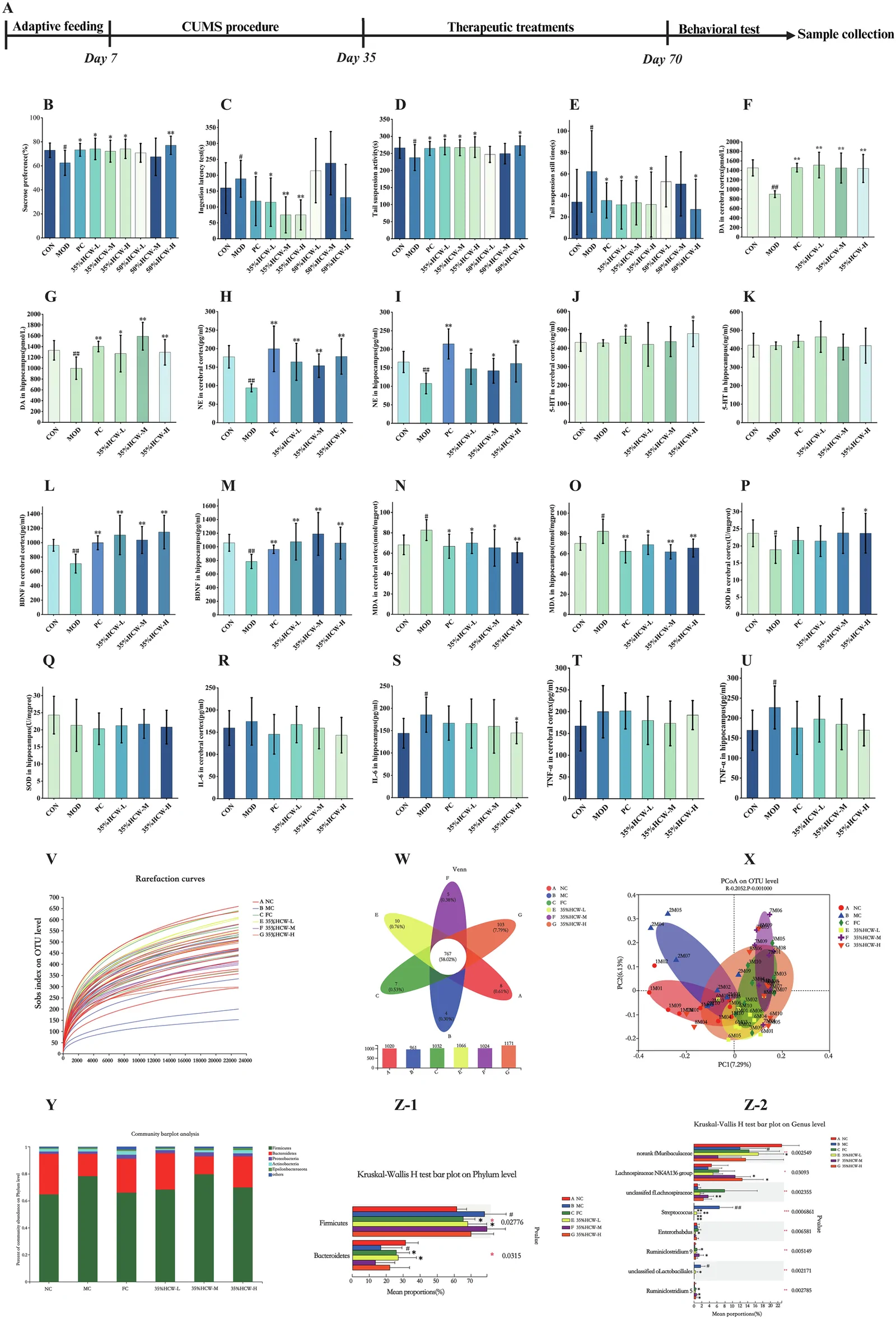
Antidepressant activity and mechanisms of 35% HCW. Timeline for evaluating the antidepressant activity of 35% HCW (**A**); Effects of 35% HCW on sucrose preference test (SPT (**B**)), ingestion latency test (ILT (**C**)), tail suspension activity time (**D**), and tail suspension still time (**E**) in CUMS-induced depressed mice; Effects of 35% HCW on cerebral cortex and hippocampal levels of Dopamine (DA (**F**, **G**)), Norepinephrine (NE (**H**, **I**)), 5-Hydroxytryptamine (5-HT (**J**, **K**)), Brain-Derived Neurotrophic Factor (BDNF (**L**, **M**)), Malondialdehyde (MDA (**N**, **O**)), Superoxide Dismutase (SOD (**P**, **Q**)), Interleukin-6 (IL-6 (**R**, **S**)), and Tumor Necrosis Factor-α (TNF-α, (**T**, **U**)) in depressed mice; Effects of 35% HCW on gut microbiota in depressed mice: Rarefaction curve (**V**), Microbial richness indices on Operational Taxonomic Unit (OTU) level (**W**, **X**)); Microbial abundance at the phylum level (**Y**); Firmicutes and Bacteroidetes relative abundance at the phylum level (**Z-1**) and variations of the eight principal intestinal microorganisms at genus level (**Z-2**). Data are expressed as mean ± SD (*n* = 10). Statistical analysis was performed by one-way ANOVA followed by Dunnett’s test (post hoc test). ^#^(*p* < 0.05) and ^##^(*p* < 0.01) compared with the normal control group; ^*^(*p* < 0.05) and ^**^(*p* < 0.01) compared with the model control group. CON control group, MOD model group, PC positive group (Fluoxetine hydrochloride). 35% HCW-L, 7.5 mg/Kg; 35% HCW-M, 15 mg/Kg; 35% HCW-H, 30 mg/Kg.

Previous research has shown that the water extract of flowering *H. citrina* (HCW) possesses the strongest antidepressant activity^12^. In this study, HCW was fractionated into two core components (35% and 50% HCW) using AB-8 macroporous adsorption resin, and the antidepressant activities of the two fractions were evaluated based on the Chronic Unpredictable Mild Stress (CUMS)-induced mouse depression model (Fig. 2A). As shown in Fig. 2, the 35% HCW (both low-, medium-, and high-doses) displays significantly activity by significantly increasing the sucrose preference level (Fig. 2B), reduced the ingestion latency (Fig. 2C), increased the tail movement time (Fig. 2D), and decreased the immobility time in the tail suspension test (Fig. 2F) of depressed mice. Furthermore, the effects of the 35% HCW displayed a dose-dependent trend from low to high doses. In contrast, the 50% HCW fraction did not exhibit significant antidepressant activity. These results indicate that the 35% HCW possesses significant antidepressant activity and was the active component group responsible for the antidepressant effect of *H. citrina*.

### 35% HCW significantly increases the levels of DA and NE in the brain of depressed mice

One-way ANOVA revealed a significant overall group effect on DA levels in the cerebral cortex, F(5, 54) = 10.69, *p* < 0.0001. Dunnett’s multiple comparisons test showed that the 35% HCW-L, 35% HCW-M, and 35% HCW-H groups had significantly higher DA levels than the MOD group (all *p* < 0.01). One-way ANOVA revealed a significant overall group effect on DA levels in the hippocampus, *F*(5, 54) = 6.92, *p* < 0.0001. Dunnett’s multiple comparisons test showed that the 35% HCW-M and 35% HCW-H groups had significantly higher DA levels than the MOD group (all *p* < 0.01).

One-way ANOVA revealed a significant overall group effect on NE levels in the cerebral cortex, *F*(5, 54) = 13.38, *p* < 0.0001. Dunnett’s multiple comparisons test showed that the 35% HCW-L, 35% HCW-M, and 35% HCW-H groups had significantly higher NE levels than the MOD group (all *p* < 0.01). One-way ANOVA revealed a significant overall group effect on NE levels in the hippocampus, *F*(5, 54) = 8.59, *p* < 0.0001. Dunnett’s multiple comparisons test showed that the 35% HCW-H groups had significantly higher NE levels than the MOD group (*p* < 0.01).

One-way ANOVA showed that there was no significant difference in 5-HT levels in the cerebral cortex among groups, *F*(5, 54) = 1.09, *p* = 0.376. One-way ANOVA showed no significant overall group effect on 5-HT levels in the hippocampus, *F*(5, 54) = 0.98, *p* = 0.439.

As shown in Fig. 2, after CUMS induced depression in normal mice, the levels of DA (Fig. 2F, G) and NE (Fig. 2H, I) in the cerebral cortex and hippocampus were significantly reduced (*p* < 0.01). Treatment with the positive control drug significantly increased the content of DA and NE (*p* < 0.01), indicating the successful establishment of the CUMS-induced mouse depression model and the appropriate selection of the positive control. Administration of low-, medium-, and high-doses of 35% HCW significantly increased DA and NE levels in the cerebral cortex and hippocampus of depressed mice (*p* < 0.05 or 0.01). However, only the high-dose of 50% HCW significantly elevated 5-HT levels in the cerebral cortex (*p* < 0.05), while the low- and medium-dose showed no significant effect (Fig. 2J, K).

### 35% HCW significantly increases the BDNF levels in the brain of depressed mice

One-way ANOVA revealed a significant overall group effect on BDNF levels in the cerebral cortex, *F*(5, 54) = 7.31, *p* < 0.0001. Dunnett’s multiple comparisons test showed that the 35% HCW-L, 35% HCW-M, and 35% HCW-H groups had significantly higher BDNF levels than the MOD group (all *p* < 0.01). One-way ANOVA revealed a significant overall group effect on BDNF levels in the hippocampus, *F*(5, 54) = 4.35, *p* = 0.0021. Dunnett’s multiple comparisons test showed that the 35% HCW-L, 35% HCW-M, and 35% HCW-H groups had significantly higher BDNF levels than the MOD group (all *p* < 0.01).

As shown in Fig. 2L, M, after depression was induced in normal mice by CUMS, the BDNF levels in the cerebral cortex and hippocampus decreased significantly (*p* < 0.01). Administration of low-, medium-, and high-doses of 35% HCW significantly increased BDNF levels in both the cerebral cortex and hippocampus of the depressed mice (*p* < 0.01). Compared with the model group, BDNF levels in the cerebral cortex increased by 62.6% in the high-dose 35% HCW group, while BDNF levels in the hippocampus increased by 51.5% in the medium-dose 35% HCW group. The effect of 35% HCW on elevating BDNF levels in depressed mice was stronger than that of fluoxetine hydrochloride.

### 35% HCW reduces brain oxidative stress indicators and inflammatory factors

One-way ANOVA revealed a significant overall group effect on MDA levels in the cerebral cortex, *F*(5, 54) = 3.84, *p* = 0.0047. Dunnett’s multiple comparisons test showed that the 35% HCW-H group had significantly lower MDA levels than the MOD group (*p* < 0.01). One-way ANOVA revealed a significant overall group effect on MDA levels in the cerebral cortex, *F*(5, 54) = 6.24, *p* < 0.001. Dunnett’s multiple comparisons test showed that the 35% HCW-L and 35% HCW-H groups had significantly lower MDA levels than the MOD group (*p* < 0.01).

One-way ANOVA showed no significant overall group effect on SOD levels in the cerebral cortex, *F*(5, 54) = 1.68, *p* = 0.156. One-way ANOVA showed no significant overall group effect on SOD levels in the hippocampus, *F*(5, 54) = 0.79, *p* = 0.564.

One-way ANOVA showed no significant overall group effect on IL-6 levels in the cerebral cortex, *F*(5, 54) = 0.74, *p* = 0.594. One-way ANOVA showed no significant overall group effect on IL-6 levels in the hippocampus, *F*(5, 54) = 1.28, *p* = 0.284.

One-way ANOVA showed no significant overall group effect on TNF-α levels in the cerebral cortex, *F*(5, 54) = 0.81, *p* = 0.550. One-way ANOVA showed no significant overall group effect on TNF-α levels in the hippocampus, *F*(5, 54) = 1.55, *p* = 0.189.

As shown in Fig. 2N–Q, during the CUMS modeling process, mice exhibited a certain degree of oxidative stress response in both the cerebral cortex and hippocampus, characterized by significantly increased MDA levels (*p* < 0.05) and decreased Superoxide Dismutase (SOD) levels (*p* < 0.05). Administration of low-, medium-, and high-doses of 35% HCW significantly reduced MDA levels in the cerebral cortex and hippocampus of depressed mice (*p* < 0.05 or 0.01), showing a certain dose-effect relationship. The medium and high doses of 35% HCW significantly increased SOD levels in the hippocampus, with effects superior to those of fluoxetine hydrochloride. As shown in Fig. 2R–U, during CUMS modeling, inflammatory factors (IL-6 and TNF-α) increased in both the cerebral cortex and hippocampus of mice. After treatment with low-, medium-, and high-doses of 35% HCW, the levels of IL-6 and TNF-α in the cerebral cortex and hippocampus of depressed mice were reduced. The high dose of 35% HCW exhibited the most potent activity, significantly lowering IL-6 levels in the hippocampus of depressed mice (*p* < 0.05) and bringing them close to normal values.

### 35% HCW improves the gut microbiota of depressed mice

As shown in Fig. 2V, the dilution curve of gut bacteria in mice reflects the richness of species in the samples, and its eventual plateau indicates the adequacy of sequencing depth in this study. Figure 2W demonstrates that 35% HCW significantly increases the abundance of gut microbiota, with the high-dose 35% HCW group showing the most pronounced effect, exhibiting a 21.8% increase in gut microbial abundance compared to the model group. PCoA (Principal Coordinates Analysis) analysis (Fig. 2X) reveals clear separation among the model group, positive control group, and different doses of 35% HCW, further indicating that 35% HCW can modulate the bacterial community in depressed mice. As shown in Fig. S6, the model group exhibited reduced bacterial α-diversity indices (Sobs, Chao, ACE, and Shannon*)*, while treatment with different doses of 35% HCW increased these indices and improved the gut microbiota composition in depressed mice.

*Bacteroidetes*, a dominant phylum in the gut, participates in bile acid, steroid, and polysaccharide metabolism, and maintains normal intestinal physiology, making it a beneficial gut bacterial group. In depressed mice, the abundance of *Bacteroidetes* significantly decreased (*p* < 0.05), while treatment with low- and high-doses of 35% HCW significantly increased its abundance (*p* < 0.05)(Fig. 2Y, Z-1). Compared to the normal group, the model group showed a significant increase in *Firmicutes* abundance (*p* < 0.05), while treatment with low-dose 35% HCW significantly reduced *Firmicutes* abundance (*p* < 0.05) (Fig. 2Y, Z-1). Analysis at the genus level revealed that the abundance of norank_f__Muribaculaceae significantly decreased in depressed mice (*p* < 0.05), while low-dose 35% HCW significantly increased its abundance (*p* < 0.05)(Fig. 2Z-2). An increased in norank_f__Muribaculaceae abundance has been shown to reduce intestinal inflammatory factors^32,33^. *Streptococcus*, a common pyogenic bacterium, can cause various suppurative inflammations such as meningitis and streptococcal hypersensitivity diseases. Compared to the model group, different doses of 35% HCW significantly reduced the abundance of *Streptococcus* (*p* < 0.01), indicating that 35% HCW exerts antidepressant effects by lowering *Streptococcus* abundance and reducing intestinal inflammation.

In summary, 35% HCW exhibits significant antidepressant activity, increasing sucrose preference fraction (SPF) and reducing immobility time in Forced Swim Test (FST) and Tail Suspension Test (TST) in depressed mice, whereas 50% HCW shows no antidepressant activity. The antidepressant effects of 35% HCW are closely associated with increased levels of DA, NE, BDNF, and SOD in the cerebral cortex and hippocampus, reduced inflammatory factors and MDA, and improved gut microbiota composition in depressed mice.

### Isolation of compounds 1 and 2 from 35% HCW

**Fig. 3:**
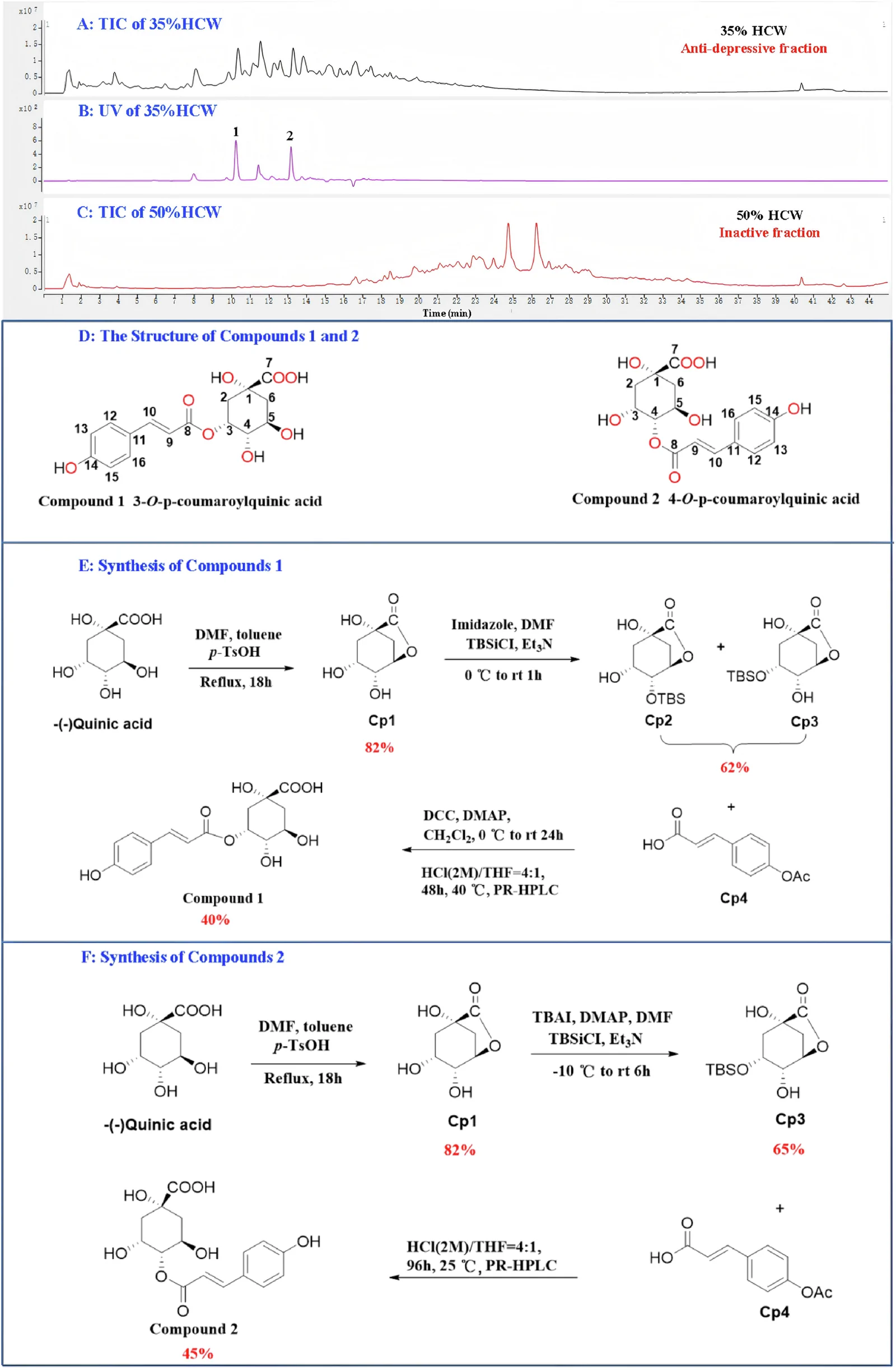
Isolation, identification, and synthesis of compounds 1 and 2. The total ion chromatogram (TIC) of 35% HCW (**A**); The ultraviolet rays (UV) spectrum of 35% HCW (300 nm, (**B)**); The TIC of 50% HCW (**C**); The structure of compounds **1** and **2** (**D**); Synthesis of compound **1** (**E**); And synthesis of compound **2** (**F**). (DCC N,N’-Dicyclohexylcarbodiimide, DMAP 4-Dimethylaminopyridine, DMF N,N-Dimethylformamide, Et_3_N Triethylamine, p-TsOH p-Toluenesulfonic acid monohydrate, TBAI Tetrabutylammonium iodide, THF tetrahydrofuran).

The 35% HCW of *H. citrina* exhibited significant antidepressant activity, while the 50% HCW did not show significant activity. Two high-content components (Compounds **1** and **2**, Fig. 3A, B) were found in the 35% HCW based on HPLC-Q/TOF-MS analysis, however, these compounds were absent in the 50% HCW (Fig. 3C) and were thus recognized as the potential antidepressant components in *H. citrina* and its 35% HCW. Guided by mass spectrometry and combined with preparative HPLC, compound **1** (12.7 mg) and compound **2** (24.6 mg) were successfully isolated. Through nuclear magnetic resonance (NMR) and high-resolution mass spectrometry (HRMS) analysis, compounds **1** and **2** were identified as 3-*O*-p-coumaroylquinic acid and 4-*O*-p-coumaroylquinic acid, respectively.

### Structural identification of compounds 1 and 2

**Compound 1:** White powder. HRMS: 337.0923 [M-H]^−^ (C₁₆H₁₇O₈). UV (MeOH) λmax: 220 and 310 nm. The theoretical mass-to-charge ratio for the deprotonated chlorogenic acid is *m/z* 353.0873 [M-H]^−^. The difference in *m/z* between compound **1** and chlorogenic acid was 15.9950, indicating that compound **1** lacks one hydroxyl group compared to the structure of chlorogenic acid. The MS/MS spectrum (Fig. S7A) shows that the parent ion (*m/z* 337.0923) undergoes ester bond cleavage to form a deprotonated fragment ion at *m/z* 163.0393, which subsequently undergoes neutral loss of a CO₂ molecule to yield a fragment ion at *m/z* 119.0500, suggesting the presence of only one hydroxyl group on the benzene ring in compound **1**. The ¹H-NMR spectrum (Fig. S7B) reveals two pairs of overlapping hydrogen signals (δ 7.51 and 6.83), and the ¹³C-NMR spectrum (Fig. S7C) also shows two pairs of overlapping carbon signals (δ 133.8 and 116.8), indicating that the hydroxyl group on the benzene ring is located at the 14-position(Fig. 3D). In the ¹H-NMR spectrum of compound **1**, the chemical shift of H-3 (δ 4.58) is greater than that of H-4 (δ 4.31) and H-5 (δ 4.22), indicating that the ester group is attached to C-3 of the quinic acid. The ¹H-¹H COSY, HMBC, and HMQC, DEPT 135 spectra (Fig. S7D–F) of compound **1** confirm that the carboxyl group of 4-hydroxycinnamic acid undergoes esterification condensation with the hydroxyl group at C-3 of quinic acid. Therefore, compound **1** was identified as 3-*O*-p-coumaroylquinic acid. The ¹H and ¹³C-NMR data of compound 1 was as follow: ^1^H-NMR (800 MHz, MeOD) *δ*_H_ 7.72(1H, *d*, *J* = 16 Hz, H-10), 7.51(2H, *d*, *J* = 16 Hz, H-12,16), 6.83(2H, *d*, *J* = 8.8 Hz, H-13,15), 6.45(1H, *d*, *J* = 15.2 Hz, H-9), 4.58(1H, *d*, H-3), 4.31(1H, *d*, H-4), 4.22(1H, *m*, H-5), 2.01 ~ 2.50 (4H, *m*, H-2,6). ^13^C-NMR(200 MHz, MeOD), *δ*_C_ 115.4(C-9), 116.8(C-13,15), 131.1(C-11), 133.8(C-12, 16), 146.7(C-10), 161.2(C-14), 168.9(C-8).

**Compound 2:** White powder. HRMS: 337.0904 [M-H]^−^ (C₁₆H₁₇O₈). UV (MeOH) *λ*max: 220 and 310 nm. Compound **2** is an isomer of compound **1** (Fig. 3D), and their primary and secondary mass spectra are nearly identical. In the ¹H-NMR spectrum of compound **2**, the chemical shift of H-4 (δ 4.81) is higher than that of H-3 (δ 4.31) and H-5 (δ 4.22), indicating that the carboxyl group of 4-hydroxycinnamic acid undergoes ester condensation with the hydroxyl group at the 4-position of quinic acid to form compound **2**. The ¹H-¹H COSY spectrum shows that H-4 only couples with H-3 and H-5, and not with H-2 or H-6. According to the HMBC spectrum, δH 4.81 (H-4) exhibits coupling with δC 168.9 (C-8), and δH 4.31 (H-3) and δH 4.22 (H-5) show couplings with δC 79.1 (C-4). This further confirms that the ester group is attached to the C-4 position of quinic acid. Therefore, compound **2** was identified as 4-*O*-p-coumaroylquinic acid (The HRMS and NMR spectra see Fig. S8A–G). The ¹H and ¹³C-NMR data of compound 2 was as follow: ^1^H-NMR (800 MHz, MeOD) *δ*_H_ 7.72(1H, *d*, *J* = 16 Hz, H-10), 7.51(2H, *d*, *J* = 16 Hz, H-12,16), 6.83(2H, *d*, *J* = 8.8 Hz, H-13,15), 6.45(1H, *d*, *J* = 15.2 Hz, H-9), 4.81(1H, *m*, H-4), 4.31(1H, *m*, H-3), 4.22(1H, *m*, H-5), 2.01 ~ 2.50 (2H, *m*, H-2,6). ^13^C-NMR(200 MHz, MeOD) *δ*_C_ 38.7(C-2), 42.3(C-6), 65.8(C-5), 69.4(C-3), 76.7(C-1), 79.1(C-4), 115.4(C-9), 116.8(C-13,15), 127.3(C-11), 131.4(C-12,16), 146.7(C-10), 168.9(C-8), 178.2(C-7).

### Synthesis of compounds 1 and 2

Large quantities of references for compounds **1** and **2** are not commercially available. Moreover, isolating and collecting these two compounds from *Hemerocallis citrina* using high-performance preparative liquid chromatography is time-consuming and labor-intensive, and also results in a substantial waste of raw *Hemerocallis citrina* materials. To further investigate their antidepressant activities and the corresponding mechanisms, it is imperative to obtain a sufficient amount of compounds **1** and **2** via a chemical synthesis method.

### Synthesis of compound 1

To further confirm the configuration of compound **1** and to provide sufficient quantities of the pure compound for subsequent antidepressant activity studies, chemical synthesis of compound **1** was performed (Fig. 3E). The synthesis started with (-)-quinic acid as the initial material. In the presence of p-toluenesulfonic acid monohydrate (p-TsOH) and a solution of *N,N*-dimethylformamide (DMF), esterification occurred between the carboxyl group at position 1 and the hydroxyl group at position 5 of (-)-quinic acid, yielding Cp**1** (yield: 82%). Cp**1** was then dissolved in DMF and cooled to 0 °C. Imidazole and tert-butyldimethyl- chlorosilane (TBDMSCl) were added, and the mixture was refluxed for 1 hour to produce a mixture of Cp**2** and Cp**3** (yield: 62%). These mixtures, along with Cp**4**, were dissolved in dichloromethane. The solution was stirred at 0 °C, then 4-dimethylaminopyridine (DMAP) and *N,N’*-dicyclohexylcarbodiimide (DCC) were added as catalysts. After stirring for 10 min, the reaction was allowed to proceed at room temperature for 24 h. The product was purified by column chromatography to obtain a white solid. This white solid was then dissolved in tetrahydrofuran, and 2 M hydrochloric acid was added. The mixture was stirred at 40 °C for 48 h. After completion, an excess of saturated sodium chloride solution was added, and the organic layer was extracted with ethyl acetate. The organic extract was dried over anhydrous sodium sulfate, and the solvent was removed under reduced pressure. Further purification by high-speed counter-current chromatography yielded a white solid. Analysis by MS and NMR spectroscopy (Fig. S9A–E) confirmed the product as 3-*O*-p-coumaroylquinic acid (yield: 40%).

### Synthesis of compound 2

The synthesis of Cp**1** followed the same procedure as described previously (Fig. 3F). Cp**1** was dissolved in DMF, followed by the sequential addition of DMAP, the tetrabutylammonium iodide, and triethylamine. Finally, tert-butyldimethylsilyl chloride was added to protect the hydroxyl group at position 3 of Cp**1**, yielding Cp**5**. Cp**4** and Cp**5** were then dissolved in dichloromethane. The solution was stirred at 0 °C until fully dissolved, after which DMAP and DCC were added as catalysts. The mixture was stirred for 10 min and then allowed to react at room temperature for 24 h. Upon completion of the reaction, the solvent was removed, and the product was preliminarily purified by column chromatography to obtain a white solid. This white solid was dissolved in tetrahydrofuran, and 2 M hydrochloric acid was added. The mixture was stirred at room temperature for 96 h. After the reaction, an excess of saturated sodium chloride solution was added to the reaction mixture, followed by extraction with ethyl acetate. The combined organic layers were dried over anhydrous sodium sulfate, and the solvent was removed under reduced pressure. Purification by high-speed counter-current chromatography yielded a white solid, which was confirmed by MS and NMR spectroscopy analysis (Fig. S10A–E) as 4-*O*-p-coumaroylquinic acid (yield: 45%).

### Compounds 1 and 2 display significant antidepressant activity

One-way ANOVA showed no significant overall group effect on sucrose preference, *F*(4, 39) = 5.258, *p* = 0.0017. Tukey’s multiple comparisons test showed that the compounds **1** and **2** groups had a significantly higher sucrose preference index than the MOD group (*p* < 0.01). One-way ANOVA showed no significant overall group effect on the still time in the tail suspension test, *F*(4, 37) = 9.259, *p* < 0.0001. Tukey’s multiple comparisons test showed that the compounds **1** and **2** groups had a significantly shorter still time than the MOD group (*p* < 0.05, *p* < 0.001). One-way ANOVA showed no significant overall group effect on immobility time in the forced swim test, *F*(4, 43) = 5.744, *p* = 0.0009. Tukey’s multiple comparisons test showed that the compounds **1** and **2** groups had a significantly shorter still time than the MOD group (*p* < 0.01)

**Fig. 4:**
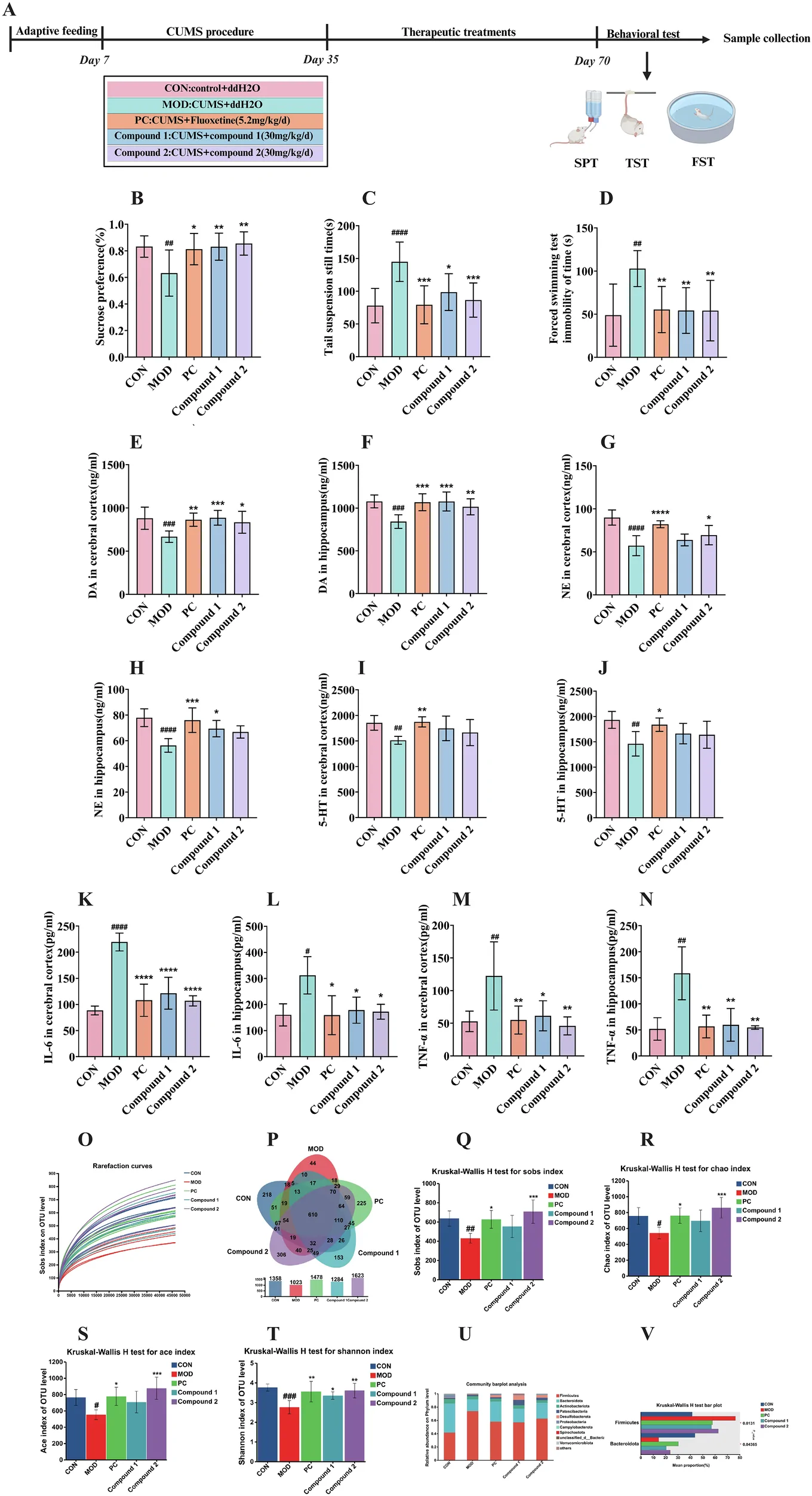
Antidepressant activity and mechanisms of compounds 1 and 2. Timeline for evaluating the antidepressant activity of compounds **1** and **2** (**A**); Effects of compounds **1** and **2** on sucrose preference test (SPT (**B**)), tail suspension still time (TST (**C**)), and forced swimming test immobility time (FST (**D**)) in CUMS-induced depressed mice; Effects of compounds **1** and **2** on cerebral cortex and hippocampal levels of Dopamine (DA (**E**, **F**), Norepinephrine (NE (**G**, **H**)), 5-Hydroxytryptamine (5-HT (**I**, **J**)), Interleukin-6 (IL-6 (**K**, **L**)), and Tumor Necrosis Factor-α (TNF-α (**M**, **N**)) in depressed mice; Effects of compounds **1** and **2** on gut microbiota in depressed mice: Rarefaction curve (**O**); Microbial richness indices on OTU level (**P**); Sobs (**Q**), Chao (**R**), ACE (**S**), and Shannon (**T**) index; Relative abundance at the phylum level (**U**); Firmicutes and Bacteroidetes relative abundance at the phylum level (**V**). Data are expressed as mean ± SD (*n* = 10). Statistical analysis was performed by one-way ANOVA followed by Tukey’s new multiple comparisons test (post hoc test). ^#^(*p* < 0.05), ^##^(*p* < 0.01), ^###^(*p* < 0.001), and ^####^(*p* < 0.0001) compared with the normal control group; ^*^(*p* < 0.05), ^**^(*p* < 0.01), ^***^(*p* < 0.001), and ^****^(*p* < 0.0001) compared with the model control grou*p*. CON, control group; MOD, model group; PC, positive group (Fluoxetine hydrochloride); compound **1** and **2** groups, 30 mg/Kg.

A mouse depression model was established using the CUMS protocol. The antidepressant effects of the positive control drug, compound **1**, and compound **2** were evaluated by observing changes in behavioral parameters (Fig. 4A). Behavioral analysis revealed that compared to the normal group, the model group exhibited a significant decrease in the SPF index (*p* < 0.05, Fig. 4B), a marked increase in immobility time in the TST (*p* < 0.01, Fig. 4C), and the FST (*p* < 0.05, Fig. 4D), indicating the successful induction of the depression model via CUMS. Compared to the model group, the fluoxetine hydrochloride group, as well as the compound **1** and **2** groups, showed a significant increase in the SPF index (*p* < 0.05 or 0.01, Fig. 4B), a significant reduction in immobility time in the TST (*p* < 0.05 or 0.001, Fig. 4C), and in the FST(*p* < 0.01, Fig. 4D). Notably, the SPF indices in the compound **1** and **2** groups were higher than that in the fluoxetine hydrochloride group. These data demonstrate that compounds **1** and **2** possess significant antidepressant activity and were the primary antidepressant components of the 35% HCW.

### Compounds 1 and 2 elevate DA and NE levels in the brain of depressed mice via the CA/Dopa/DA/NE pathway

One-way ANOVA revealed a significant overall group effect on DA levels in the cerebral cortex, *F*(4, 39) = 5.849, *p* = 0.0009. Tukey’s multiple comparisons test showed that the compound **1** group had significantly higher DA levels than the MOD group (*p* < 0.001). One-way ANOVA revealed a significant overall group effect on DA levels in the hippocampus, *F*(4, 36) = 8.442, *p* < 0.0001. Tukey’s multiple comparisons test showed that the compounds **1** and **2** groups had significantly higher DA levels than the MOD group (*p* < 0.001 and *p* < 0.01).

One-way ANOVA revealed a significant overall group effect on NE levels in the cerebral cortex, *F*(4, 43) = 18.78, *p* < 0.0001. Tukey’s multiple comparisons test showed that the compound **2** group had significantly higher NE levels than the MOD group (*p* < 0.05). One-way ANOVA revealed a significant overall group effect on NE levels in the hippocampus, *F*(4, 35) = 7.115, *p* = 0.0003. Tukey’s multiple comparisons test showed that the compound **1** group had significantly higher NE levels than the MOD group (*p* < 0.05).

One-way ANOVA revealed a significant overall group effect on 5-HT levels in the cerebral cortex, *F*(4, 38) = 5.424, *p* = 0.0015. One-way ANOVA revealed a significant overall group effect on 5-HT levels in the hippocampus, *F*(4, 34) = 5.370, *p* = 0.0018.

Compared to the normal group, the model group showed a significant decrease in DA levels in both the cerebral cortex and hippocampus (*p* < 0.001) (Fig. 4E, F). In comparison to the model group, the fluoxetine hydrochloride group exhibited significantly increased DA levels (*p* < 0.01, *p* < 0.001). Compound **1** (*p* < 0.001) and compound **2** (*p* < 0.05, 0.01) significantly elevated DA levels in the cerebral cortex and hippocampus of depressed mice. Notably, compound **1** demonstrated a more potent effect in increasing DA levels than that of fluoxetine hydrochloride. Compared to the normal group, the model group displayed a significant reduction in NE levels in both the cerebral cortex and hippocampus (*p* < 0.0001, 0.001) (Fig. 4G, H). Fluoxetine hydrochloride significantly increased NE levels compared to the model control group (*p* < 0.0001, *p* < 0.01). Compound **1** significantly elevated NE levels in the hippocampus of depressed mice (*p* < 0.05), while compound **2** significantly increased NE levels in the cerebral cortex (*p* < 0.05). The positive control drug significantly increased 5-HT levels in the cerebral cortex and hippocampus of depressed mice (*p* < 0.001, *p* < 0.005) (Fig. 4I, J). Although compounds **1** and **2** also increased 5-HT levels in the cerebral cortex and hippocampus of depressed mice, the extent of increase was not statistically significant compared to the model group.

**Fig. 5:**
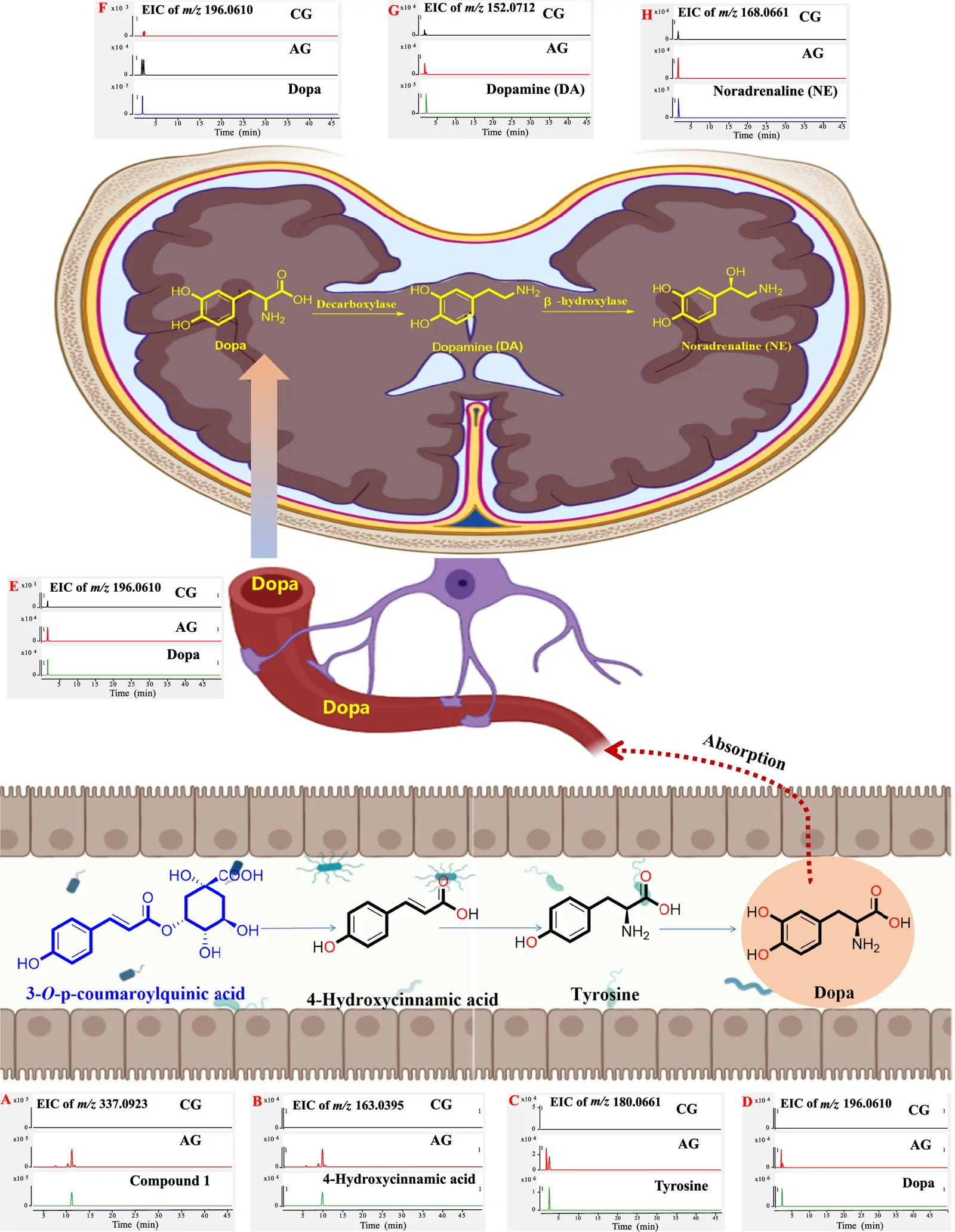
Compound **1** increases the DA and NE levels on the cerebral cortex of depressed mice via CA/Dopa/DA/NE pathway. Created in BioRender. Jiang, F. (2026) https://BioRender.com/jv9ev6l. The extracted ion chromatogram (EIC) of 3-*O*-coumaroylquinic acid (*m/z* 337.0923 (**A**)), 4-hydroxycinnamic acid (*m/z* 163.0395 (**B**)), tyrosine (*m/z* 180.0661 (**C**)), and dopa (*m/z* 196.0610 (**D**)) from the TICs of control group (CG), Administration group (AG) intestinal contents, and corresponding standard; The EIC of dopa (*m/z* 196.0610) from the TIC of serum in CG, AG, and the dopa standard (**E**); The EIC of dopa (*m/z* 196.0610 (**F**)), Dopamine (DA, *m/z* 152.0712 (**G**)), and Norepinephrine (NE, *m/z* 168.0661 (**H**)) from the TICs of cerebral cortex in CG, AG, and corresponding standard.

While 3-*O*-p-coumaroylquinic acid (**1**) and 4-*O*-p-coumaroylquinic acid (**2**) can increase the levels of DA and NE in the cerebral cortex and hippocampus of depressed mice, the specific mechanism underlying this effect remains unknown. To investigate how compounds **1** and **2** elevate DA and NE levels in the brains of depressed mice, compound **1** was selected as a representative (compounds **1** and **2** are isomers), and its in vivo metabolism in mice was studied. Using mass spectrometry, compound **1** (*m/z* 337.0923, Fig. 5A) was found to be metabolized in the intestine to 4-hydroxycinnamic acid (*m/z* 163.0395, Fig. 5B). Simultaneously, the metabolites of 4-hydroxycinnamic acid, namely tyrosine (*m/z* 180.0661, Fig. 5C) and Dopa (*m/z* 196.0610, Fig. 5D), were also detected in the intestinal contents of mice. These metabolites were scarcely detectable in the intestinal contents of the control group. Therefore, we hypothesize that 3-*O*-p-coumaroylquinic acid is metabolized in the intestine to 4-hydroxycinnamic acid, which is then converted to tyrosine under the action of intestinal transaminases, and tyrosine is subsequently hydroxylated to Dopa by intestinal hydroxylases. Dopa was also detected in mouse blood (Fig. 5E); however, the Dopa content in the group treated with 3-*O*-p-coumaroylquinic acid was significantly higher than that in the control group, indicating that Dopa produced in the intestine is absorbed into the bloodstream, leading to elevated blood Dopa levels. In the cerebral cortex of depressed mice, the levels of Dopa (Fig. 5F), DA(Fig. 5G), and NE(Fig. 5H) were higher than those of the control group. This suggests that Dopa from the blood enters the hippocampal region or cerebral cortex of the brain, where it synthesizes more DA and NE. Since 3-*O*-p-coumaroylquinic acid and 4-*O*-p-coumaroylquinic acid are isomers, it is inferred that the metabolic pathway of 4-*O*-p-coumaroylquinic acid in vivo is consistent with that of 3-*O*-p-coumaroylquinic acid. Therefore, we propose that compounds **1** and **2** are metabolized to Dopa in the intestine. This Dopa is absorbed into the bloodstream through the small intestine and is utilized in the hippocampus or cerebral cortex to synthesize DA and NE, thereby exerting an antidepressant effect (CA/Dopa/DA/NE pathway). We have discovered and elucidated that compounds **1** and **2** elevate the levels of DA and NE in depressed mice through the CA/Dopa/DA/NE pathway for the first time.

### Compounds 1 and 2 significantly reduce the levels of IL-6 and TNF-α in the cerebral cortex and hippocampus of depressed mice

One-way ANOVA showed no significant overall group effect on IL-6 levels in the cerebral cortex, *F*(4, 20) = 26.61, *p* < 0.0001. Tukey’s multiple comparisons test showed that the compounds **1** and **2** groups had significantly lower IL-6 levels than the MOD group (all *p* < 0.0001). One-way ANOVA showed no significant overall group effect on IL-6 levels in the hippocampus, *F*(4, 20) = 9.017, *p* = 0.0002. Tukey’s multiple comparisons test showed that the compounds **1** and **2** groups had significantly lower IL-6 levels than the MOD group (all *p* < 0.05).

One-way ANOVA showed no significant overall group effect on TNF-α levels in the cerebral cortex, *F*(4, 20) = 5.523, *p* = 0.0037. Tukey’s multiple comparisons test showed that the compounds **1** and **2** groups had significantly lower TNF-α levels than the MOD group (*p* < 0.05 and *p* < 0.01). One-way ANOVA showed no significant overall group effect on TNF-α levels in the hippocampus, *F*(4, 20) = 10.92, *p* < 0.0001. Tukey’s multiple comparisons test showed that the compounds **1** and **2** groups had significantly lower TNF-α levels than the MOD group (all *p* < 0.01).

Compared with the normal group, the levels of IL-6 in both the cerebral cortex and hippocampus of the model group were significantly increased (*p* < 0.0001, *p* < 0.05, Fig. 4K), indicating that the CUMS modeling process induced an inflammatory response in the mice's brain, leading to the release of inflammatory factors. Compared with the model group, compounds **1** and **2** significantly reduced the level of the inflammatory factor IL-6 in the cerebral cortex of depressed mice (*p* < 0.0001). Their efficacy in lowering IL-6 levels was comparable to that of the positive control drug, with compound **2** exhibiting stronger activity than compound **1**. Moreover, compounds **1** and **2** also significantly reduced the level of IL-6 in the hippocampus of mice (Fig. 4L).

Compared with the normal group, the levels of TNF-α in both the cerebral cortex and hippocampus of the model group were significantly elevated (*p* < 0.01). compounds **1** and **2** significantly reduced the levels of the inflammatory factor TNF-α in the cerebral cortex (*p* < 0.05, *p* < 0.01, Fig. 4M) and hippocampus (*p* < 0.01, *p* < 0.01, Fig. 3N) of the depressed mice. The reductions in TNF-α levels in the cerebral cortex and hippocampus achieved by compounds **1** and **2** were similar to those observed with the positive control drug.

### Compounds 1 and 2 improve the gut microbiota of depressed mice

The bacterial dilution curve in mouse intestines reflects the species richness within the samples, and its eventual plateau indicates the sufficiency of the sequencing data (Fig. 4O). Following the induction of depression in mice from the normal group, the number of gut microbial operational taxonomic units (OTUs) decreased by 24.6%. After treatment with compounds **1** and **2**, the OTU numbers increased by 25.5% and 58.6%, respectively. This indicates that treatment with compounds **1** and **2** enriched the gut microbiota in depressed mice, with compound **2** demonstrating a superior effect compared to compound **1** (Fig. 4P). Analysis of gut microbial α-diversity indices showed that, compared to the model group, compound **2** significantly increased the Sobs (*p* < 0.001, Fig. 4Q), Chao1 (*p* < 0.01, Fig. 4R), ACE (*p* < 0.001, Fig. 4S), and Shannon (*p* < 0.001, Fig. 4T) indices in depressed mice. Compound **1** significantly increased the Shannon index (*p* < 0.05, Fig. 4R). These data suggest that both compounds **1** and **2** can enhance the α-diversity of gut microbiota, with compound **2** showing a more pronounced effect than compound **1**.

The top five most abundant bacterial phyla were *Firmicutes*, *Bacteroidetes*, *Actinobacteria*, *Patescibacteria*, and *Desulfobacterota* (Fig. 4U). Compared to the normal group, the model group exhibited a significant increase in the abundance of the *Firmicutes* phylum (*p* < 0.01). Treatment with compounds **1** (*p* < 0.01) and **2** (*p* < 0.05) significantly reduced the *Firmicutes* abundance. Conversely, the model group showed a significant decrease in the abundance of the *Bacteroidetes* phylum compared to the normal group (*p* < 0.05). Treatment with compounds **1** and **2** increased the *Bacteroidetes* abundance. The changes in *Firmicutes* and *Bacteroidetes* observed in the compound **1** and **2** treatment groups were consistent with those of 35% HCW group (Fig. 4U, V).

### Compounds 1 and 2 elevate brain BDNF levels in the brain of depressed mice via the PKA/CREB/BDNF pathway

One-way ANOVA revealed a significant overall group effect on BDNF levels in the cerebral cortex, *F*(4, 39) = 27.36, *p* < 0.0001. Tukey’s multiple comparisons test showed that the compounds **1** and **2** groups had significantly higher BDNF levels than the MOD group (all *p* < 0.0001).

One-way ANOVA revealed a significant overall group effect on BDNF levels in the hippocampus, *F*(4, 32) = 17.64, *p* < 0.0001. Tukey’s multiple comparisons test showed that the compounds **1** and **2** groups had significantly higher BDNF levels than the MOD group (all *p* < 0.0001).

After treatment with H-89, one-way ANOVA revealed a significant overall group effect on BDNF levels in the cerebral cortex, *F*(6, 63) = 36.04, *p* < 0.0001. Tukey’s multiple comparisons test showed that the compounds **1** and **2** groups had significantly higher BDNF levels than the MOD group (all *p* < 0.0001).

After treatment with H-89, one-way ANOVA revealed a significant overall group effect on BDNF levels in the hippocampus, *F*(6, 59) = 15.43, *p* < 0.0001. Tukey’s multiple comparisons test showed that the compounds **1** and **2** groups had significantly higher BDNF levels than the MOD group (*p* < 0.0001).

**Fig. 6:**
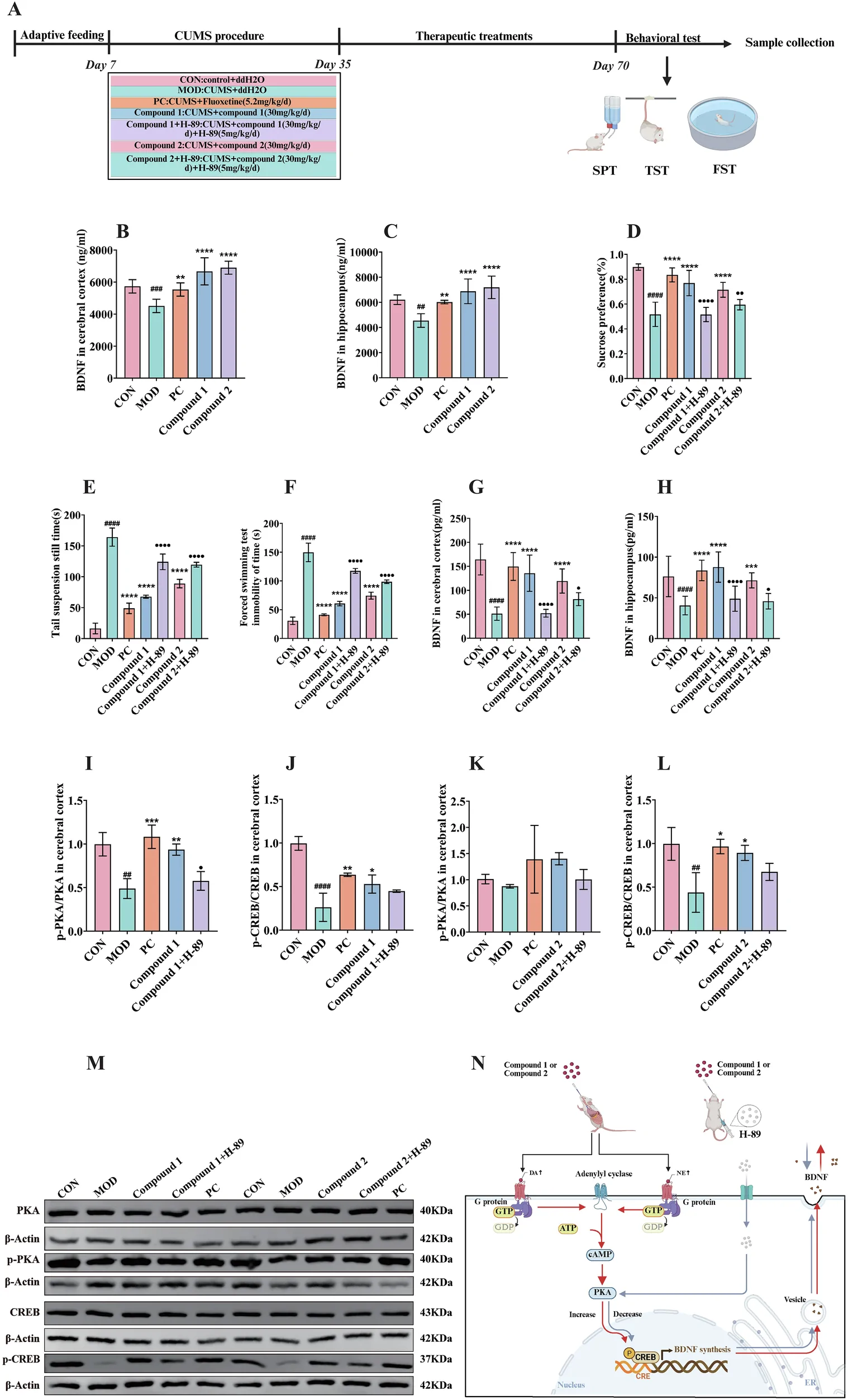
Compound 1 and 2 increase the BDNF levels on the cerebral cortex and hippocampus of depressed mice via PKA/CREB/BDNF pathway. Timeline for evaluating the antidepressant activity of compound **1**, compound **1** + H-89, and compound **2**, and compound **2** + H-89 (**A**); compounds **1** and **2** significant increase the Brain-Derived Neurotrophic Factor (BDNF) level on the cerebral cortex (**B**) and hippocampus (**C**) of depressed mice; Effects of compound **1**, compound **1** + H-89, and compound **2**, and compound **2** + H-89 on sucrose preference test (SPT (**D**)), tail suspension still time (TST (**E**)), forced swimming test immobility time (FST (**F**)), and BDNF (**G**, **H**) in CUMS-induced depressed mice; The p-PKA/PKA (Protein Kinase A) and p-CREB/CREB (cAMP Response Element-Binding Protein) level of compound **1**, compound **1** + H-89, compound **2**, and compound **2** + H-89 on the cerebral cortex (**I**, **J**, **M**) and hippocampus (**K**, **L**, **M**) of depressed mice; compound **1** and **2** increase the BDNF levels via PKA/CREB/BDNF pathway activate by the increased DA and NE in the cerebral cortex and hippocampus of depressed mice (**N**). Data are expressed as mean ± SD (*n* = 10). Statistical analysis was performed by one-way ANOVA followed by Tukey’s new multiple comparisons test (post hoc test). ^##^(*p* < 0.01), ^###^(*p* < 0.001), and ^####^(*p* < 0.0001) compared with the normal control group; ^*^(*p* < 0.05), ^**^(*p* < 0.01), ^***^(*p* < 0.001), and ^****^(*p* < 0.0001) compared with the model control group; ^**·**^(*p* < 0.05), ^**··**^(*p* < 0.01), and ^**····**^(*p* < 0.0001) compared with the compound **1** or **2** group. CON, control group; MOD, model group; PC, positive group (Fluoxetine hydrochloride); compound **1** and **2** groups, 30 mg/Kg; compound **1** + H-89, 30 + 5 mg/Kg; compound **2** + H-89, 30 + 5 mg/Kg.

Clinical observations indicate that deficiencies and metabolic dysregulation of BDNF, which is responsible for neurogenesis, trophism, and remodeling in the cerebral cortex and hippocampal regions, are significant contributors to depression^34^. Our study found that BDNF levels were significantly reduced in both the cerebral cortex and hippocampus of mice following the induction of depression (*p* < 0.001, *p* < 0.01). Treatment with compounds **1** and **2** resulted in a significant increase in BDNF levels in both brain regions (*p* < 0.0001). Notably, the elevation of BDNF levels achieved with these compounds exceeded that of the positive control drug, fluoxetine hydrochloride. Furthermore, the therapeutic effect of compound **2** was superior to that of compound **1** (Fig. 6A–C).

The PKA/CREB/BDNF pathway is closely associated with BDNF levels. To investigate whether compounds **1** and **2** increase BDNF levels in the cerebral cortex and hippocampus of depressed mice through this pathway, we utilized an inhibitor called H-89^34,35^. H-89 binds to the PKA receptor, inhibits PKA expression, thereby suppressing the PKA/CREB/BDNF pathway and reducing BDNF levels in the cerebral cortex and hippocampus. Therefore, we conducted further antidepressant experiments with compounds **1** and **2**, as well as compound **1** + H-89 and compound **2** + H-89 (Fig. 6A).

As shown in Fig. 6D, compounds **1** and **2** significantly increased the SPF index in depressed mice (*p* < 0.0001). However, when depressed mice were treated with Compound **1** + H-89 or compound **2** + H-89, their sucrose SOF index significantly decreased compared to the group treated with compounds **1** or **2** alone (*p* < 0.0001, *p* < 0.01), and the compound **1** + H-89 or compound **2** + H-89 groups no longer exhibited antidepressant activity. From Fig. 6E, compounds **1** and **2** significantly reduced the immobility time in the tail suspension test (*p* < 0.0001). In contrast, treatment with compound **1** + H-89 or compound **2** + H-89 significantly increased the immobility time (*p* < 0.0001) compared to the group treated with compounds **1** or **2** alone. Figure 6F shows that compounds **1** and **2** significantly decreased the immobility time in the forced swim test (*p* < 0.001, *p* < 0.0001). However, when treated with compound **1** + H-89 or compound **2** + H-89, the immobility time significantly increased (*p* < 0.0001) compared to the group treated with compounds **1** or **2** alone. Through the sucrose preference test, tail suspension test, and forced swim test, we have once again confirmed that compounds **1** and **2** can ameliorate depression-like behaviors in mice. However, when compounds **1** and **2** were co-administered with the inhibitor H-89, there was essentially no improvement in the depression-like behaviors of the depressed mice, indicating that the compounds lost their antidepressant activity upon the addition of H-89.

Compared to the normal group, the BDNF content in both the cerebral cortex (Fig. 6G) and hippocampus (Fig. 6H) of the model group mice was significantly decreased (*p* < 0.0001). Compounds **1** and **2** significantly increased BDNF levels in the cerebral cortex (*p* < 0.0001) and hippocampus (*p* < 0.0001, *p* < 0.001) of depressed mice. However, upon administration of H-89, the BDNF content in both the cerebral cortex and hippocampus of depressed mice showed a significant decrease (*p* < 0.0001, *p* < 0.05). Furthermore, the BDNF levels in the compound **1** + H-89 and compound **2** + H-89 groups were comparable to those in the model group (Fig. 5G). These experimental results further demonstrate that compounds **1** and **2** can significantly elevate BDNF levels in the cerebral cortex and hippocampus of depressed mice. However, after administering the inhibitor H-89, BDNF levels in these brain regions significantly decreased, reaching levels similar to the model group, indicating that H-89 markedly inhibits BDNF expression of the cerebral cortex and hippocampus in mice.

How does the inhibitor H-89 affect BDNF production via the PKA/CREB/BDNF pathway? We performed Western blot (WB) analysis on PKA, p-PKA, CREB, and p-CREB within the PKA/CREB/BDNF pathway (Figs. S11–S14). Compared to the normal group (Fig. 6I), the expression level of p-PKA/PKA in the cerebral cortex of the model group mice was significantly decreased. After treatment with compound **1**, the p-PKA/PKA expression level significantly increased (*p* < 0.01). In contrast, the p-PKA/PKA expression level in the compound **1** + H-89 group was significantly lower than that in the compound **1** group (*p* < 0.05), indicating that H-89 binds to PKA and inhibits PKA protein expression. Compared to the normal group (Fig. 6J), the expression level of p-CREB/CREB in the cerebral cortex of the model group mice was significantly decreased (*p* < 0.0001). After treatment with compound **1**, the p-CREB/CREB expression level significantly increased (*p* < 0.05). However, the p-CREB/CREB expression level in the compound **1** + H-89 group was lower than that in the compound **1** group, demonstrating that H-89, by binding to PKA, also inhibits the expression of the downstream protein CREB, ultimately affecting BDNF levels. Compound **2** exhibited effects similar to those of compound **1** (Fig. 6K–M). The above experimental data indicate that H-89, by inhibiting PKA activity, leads to a significant reduction in the expression of the downstream protein CREB, ultimately causing a significant decrease in BDNF levels (Fig. 6N). This further confirms that compounds **1** and **2** elevate BDNF levels in the brains of depressed mice by activating the PKA/CREB/BDNF pathway, thereby achieving an antidepressant effect.

Why can compounds **1** and **2** elevate BDNF levels in the brains of depressed mice by activating the PKA/CREB/BDNF pathway? As reported in the literature^34,35^, DA primarily acts on D1-class receptors (D1/D5), while NE activates β-adrenergic receptors (β1/β2). Both types of receptors couple with stimulatory G proteins, thereby activating adenylate cyclase and increasing intracellular cAMP (cyclic Adenosine Monophosphate) levels. The elevated cAMP activates protein kinase A (PKA), and phosphorylated PKA (p-PKA) further activates CREB. Phosphorylated CREB (pCREB) binds to the promoter region of the BDNF gene, driving BDNF transcription and synthesis, which ultimately raises BDNF levels in the brain. In this study, compounds **1** and **2** significantly increased DA and NE levels in the cerebral cortex and hippocampus via the CA/Dopa/DA/NE pathway. Subsequently, DA and NE can activate their respective G protein-coupled receptors, triggering the cAMP/PKA/CREB/BDNF signaling pathway and thereby promoting the expression of BDNF (Fig. 6N).

## Discussion

The present study demonstrates that the 35% HCW fraction, but not the 50% HCW fraction, is the principal antidepressant-active fraction of *Hemerocallis citrina*. In the CUMS model, 35% HCW consistently improved anhedonia- and despair-related behaviors, increased DA and NE levels in the cerebral cortex and hippocampus, elevated BDNF, attenuated oxidative stress and inflammatory responses, and partially normalized gut microbiota composition. Bioactivity-guided isolation further identified 3-*O*-p-coumaroylquinic acid and 4-*O*-p-coumaroylquinic acid as major candidate active constituents, because both compounds reproduced the key antidepressant-like effects of the active fraction. These findings are consistent with previous studies showing antidepressant-like activity of *H. citrina* extracts and phenolic-enriched fractions, and extend them by linking a defined active fraction to specific compounds and mechanisms^12,36–38^.

Why did the 50% HCW fraction fail to show antidepressant-like efficacy, whereas the 35% HCW fraction was clearly active? Our data suggest that this difference is biologically meaningful rather than incidental. HPLC-Q/TOF-MS analysis showed that 3-*O*-p-coumaroylquinic acid and 4-*O-*p-coumaroylquinic acid were present in 35% HCW but absent from 50% HCW (50%HCW mainly contains fatty acid compounds such as pinellic acid and dehydroxypinellic acid), strongly suggesting that the separation procedure enriched antidepressant-relevant constituents in the 35% fraction while excluding them from the 50% fraction. Moreover, the 50% HCW fraction produced only a limited neurochemical signal, namely a modest increase in cortical 5-HT at high dose, without consistent behavioral improvement, neurotrophic recovery, or anti-inflammatory effects. This suggests that altering a single transmitter in one brain region is insufficient to reproduce the broader antidepressant-like phenotype observed with 35% HCW. Another plausible explanation is that the 35% HCW fraction preserves a favorable multi-component phytochemical composition and potential synergistic interactions among phenolic constituents that are weakened or lost in the 50% fraction. Such fraction-specific enrichment and synergy are common features of botanical preparations and may explain why only selected fractions retain pharmacological activity after separation^12,36,37,39^.

The present findings should also be interpreted beyond a narrow monoamine-deficiency framework. Current research increasingly supports depression as a heterogeneous disorder involving interacting abnormalities in neurotransmission, synaptic plasticity, immune signaling, stress adaptation, and gut–brain communication rather than a simple serotonin deficit alone^20,40–43^. In this context, the simultaneous restoration of DA/NE, the increase in BDNF, and the reduction in inflammatory markers after 35% HCW treatment suggest a coordinated multi-system action. This interpretation is compatible with contemporary models of depression that emphasize network-level dysfunction, impaired emotional and cognitive flexibility, and convergent downstream mechanisms of antidepressant action^41–43^.

Our data indicate that catecholaminergic regulation may be especially relevant to the activity of the 35% HCW fraction and compounds **1** and **2**. In both the fraction study and the isolated-compound study, the active treatments significantly restored DA and NE in the cerebral cortex and hippocampus, whereas their influence on 5-HT was weak or statistically non-significant. This pattern suggests that the antidepressant-like effects of the active fraction may depend more on catecholaminergic restoration than on serotonergic modulation. This is pharmacologically plausible because mesocorticolimbic dopaminergic signaling and noradrenergic transmission are both critically involved in motivation, reward processing, arousal, and stress adaptation, domains that are frequently disrupted in depression^20,41,44,45^.

The proposed CA/Dopa/DA/NE pathway provides an interesting mechanistic hypothesis for these catecholaminergic effects. Our mass spectrometric data suggest that compound **1** can be metabolized in the intestine to 4-hydroxycinnamic acid and then to tyrosine- and Dopa-related intermediates, with increased Dopa in blood and elevated Dopa, DA, and NE in brain tissue. These findings support the possibility that intestinal biotransformation contributes to the neurochemical effects of compounds **1** and **2**. At the same time, this pathway should be presented cautiously as a working model rather than a fully established metabolic route. Stable-isotope tracing, enzyme inhibition, and formal pharmacokinetic studies will be needed to determine whether the detected metabolites directly contribute to catecholamine synthesis in vivo. Still, the hypothesis is strengthened by previous studies showing that *p*-coumaric acid itself has antidepressant-like and neuroprotective actions and can modulate neuroinflammation and neuroplasticity-related pathways^46–49^.

Another key finding of this study is the strong association between behavioral improvement and restoration of the PKA/CREB/BDNF pathway. BDNF is a central regulator of neuronal survival, dendritic remodeling, synaptic plasticity, and resilience to stress, and impaired BDNF signaling has been repeatedly linked to depression and antidepressant response^40,44,50–53^. In our study, 35% HCW increased BDNF in both the cerebral cortex and hippocampus, and compounds **1** and **2** produced even more pronounced elevations. Importantly, these effects were largely abolished by H-89, accompanied by reversal of behavioral improvement and suppression of p-PKA/PKA and p-CREB/CREB. These results strongly support the conclusion that activation of the PKA/CREB/BDNF cascade is an essential component of the antidepressant-like mechanism of the isolated compounds. Because DA and NE can activate D1-like and β-adrenergic receptors coupled to cAMP signaling, the observed rise in catecholamines may plausibly converge on the PKA/CREB/BDNF axis and thereby enhance neuroplasticity^40,50,52,53^.

The anti-inflammatory and antioxidant findings are likewise important for interpreting the present data. Chronic stress increased MDA and reduced SOD, while also elevating IL-6 and TNF-α in the cerebral cortex and hippocampus. Treatment with 35% HCW reversed these changes, and compounds **1** and **2** similarly reduced IL-6 and TNF-α in both brain regions. These results fit well with the current view that inflammatory dysregulation and oxidative imbalance are not merely secondary correlates of depression, but participate directly in its pathophysiology and may contribute to impaired neuroplasticity, altered neurotransmission, and treatment resistance^42,54–56^. Thus, the elevation of BDNF observed here may reflect not only direct signaling activation but also improvement of the inflammatory and oxidative microenvironment in the brain. This interpretation is supported by recent translational literature proposing immune modulation as a meaningful component of antidepressant action^42,54–56^.

The gut microbiota data further support a microbiota–gut–brain mechanism. In the fraction experiment, 35% HCW increased microbial richness and α-diversity, elevated Bacteroidetes and norank_f__Muribaculaceae, and reduced Firmicutes and Streptococcus. In the isolated-compound experiment, both compounds increased OTU richness and α-diversity, with compound **2** showing a stronger effect than compound **1**, and both compounds shifted the Firmicutes/Bacteroidetes pattern in a direction consistent with the active fraction. These findings are biologically relevant because growing evidence indicates that gut microbiota can influence depressive phenotypes through immune, endocrine, metabolic, and neural pathways, and that stress-related dysbiosis can promote peripheral inflammation and altered brain function. The convergence between our microbiota results and your reductions in IL-6 and TNF-α supports the idea that microbiota remodeling may be upstream of at least part of the anti-inflammatory and antidepressant-like effect. However, the present data remain associative. Definitive proof of causality will require fecal microbiota transplantation, microbiota depletion, or gnotobiotic approaches^57–64^.

The comparison between compound **1** and compound **2** also deserves discussion. Although both isomers showed clear antidepressant-like activity, compound **2** generally produced stronger effects on gut microbiota diversity and BDNF elevation, whereas compound **1** appeared somewhat more potent for certain catecholaminergic indices. This suggests that positional isomerism may influence intestinal metabolism, tissue exposure, target engagement, or microbiota interactions, even when the compounds share the same molecular formula. Therefore, the antidepressant activity of *H. citrina* should not be interpreted as the action of a single universal molecule, but rather as a chemically nuanced effect involving structurally related phenylpropanoid derivatives with overlapping but non-identical biological profiles^36,46–49^.

From a translational standpoint, the present study advances *H. citrina* from a traditional antidepressant-associated plant toward a better-defined pharmacological system. The 35% HCW fraction may represent a standardized antidepressant-relevant fraction, whereas 3-*O*-p-coumaroylquinic acid and 4-*O*-p-coumaroylquinic acid may serve as candidate quality markers and lead molecules. This step from crude extract to chemically and mechanistically defined actives is important for future development of food-derived neuroactive preparations and natural product-based antidepressant strategies^12,37,38,57,58^.

Several limitations should nevertheless be acknowledged. First, all experiments were performed in a CUMS model, which is highly informative but cannot capture the full biological heterogeneity of human depression^65^. Second, the CA/Dopa/DA/NE pathway remains inferential and awaits direct metabolic validation. Finally, the microbiota findings, though compelling, do not establish causality. Addressing these issues will help determine whether *H. citrina* is best developed as a defined active fraction, a multi-component botanical preparation, or a source of optimized small-molecule leads^57,58,65^.

Overall, the present work shows that 35% HCW is the principal antidepressant-active fraction of *H. citrina* and that 3-*O*-p-coumaroylquinic acid and 4-*O*-p-coumaroylquinic acid are important active constituents. Their antidepressant-like effects appear to involve coordinated regulation of catecholaminergic neurotransmission, the PKA/CREB/BDNF axis, neuroinflammation, oxidative stress, and gut microbiota. The lack of effect of 50% HCW further emphasizes the importance of fraction-specific phytochemical enrichment and possibly multi-component synergy. Taken together, these findings provide a more complete mechanistic basis for the antidepressant potential of *H. citrina* and justify further pharmacological and translational investigation^12,36–38,40,50,57^.

In this study, we identified for the first time that 35% HCW was the active antidepressant fraction of *H. citrina*. Utilizing mass spectrometry, we pinpointed 3-*O*-p-coumaroylquinic acid (**1**) and 4-*O*-p-coumaroylquinic acid (**2**) as the potential antidepressant constituents of *H. citrina* and 35% HCW. Guided by mass spectrometry, these two compounds were isolated, and their structures were elucidated via NMR and HRMS. Subsequently, both compounds were synthesized using chemical methods. Antidepressant activity evaluation revealed that compounds **1** and **2** possess significant antidepressant activity, identifying them as the primary active components responsible for the antidepressant effects of *H. citrina* and 35% HCW. Further mechanistic studies demonstrated that compounds **1** and **2** significantly elevate the levels of DA and NE in the brains of depressed mice via the CA/Dopa/DA/NE pathway and significantly increase BDNF levels through the PKA/CREB/BDNF pathway activate by the increased DA and NE. Concurrently, compounds **1** and **2** also ameliorated gut microbiota and reduced the levels of inflammatory factors IL-6 and TNF-α in the brain of depressed mice.

In summary, 35% HCW from *H. citrina*, along with compounds **1** and **2**, exert antidepressant effects by elevating the levels of DA, NE, and BDNF, reducing the levels of IL-6, TNF-α, and MDA in the brains of depressed mice, and improving gut microbiota composition. This research lays a foundation for the development of novel antidepressants based on 35% HCW, compounds **1** or **2**, and for the high-value utilization of *H. citrina* resources.

## Methods

### Materials and chemicals on the antidepressant effects of 35% HCW

An aqueous extract of 1.0 kg from flowering *H. citrina* was used to prepare different fractions. AB-8 macroporous adsorption resin and ethanol, purchased from Sinopharm Chemical Reagent Co., Ltd. (Shanghai, China), were employed for the preliminary separation of the aqueous extract. Fluoxetine hydrochloride (Eli Lilly Suzhou Pharmaceutical Co., Ltd., USA) was used as the positive control drug. Mouse ELISA (Enzyme-Linked Immunosorbent Assay) kits for 5-HT, NE, BDNF, DA, TNF-α, and IL-6 (MU30044, 100 tubes/96 wells) were purchased from Bioswamp (China) for the determination of monoamine neurotransmitters, BDNF, and inflammatory factors of the brain. Kits for malondialdehyde (MDA) and superoxide dismutase (SOD)(MS1401, 100 tubes/96 wells, Suoqiao Biology, China) were used to measure peroxidation levels in the cerebral cortex and hippocampus of the brain.

### Preparation of 35% HCW

The *H. citrina* (Mengzihua) samples were collected from Huangtupu Town, Qidong County, Hunan Province (latitude 26°53′23.75″N, longitude 111°52′22.44″E). It was identified as *Hemerocallis citrina* Baroni (Mengzihua cultivar) by Associate Professor Qing Zhixing from Hunan Agricultural University. Voucher specimen has been deposited at the Herbarium of Hunan Agricultural University with the accession number (Mengzihua-20240618). Fresh *H. citrina* weighing 300 Kg were chopped and placed in 1500 L barrels. A total of 900 L of water was added to completely submerge the *H. citrina* samples, followed by cold immersion extraction for 24 h. After filtration, the extract was collected and concentrated to 35 L using a large-scale rotary evaporator (55–60 °C). A portion of the extract was further concentrated to complete dryness. The aqueous extract of *H. citrina* was eluted through AB-8 macroporous adsorption resin, yielding the water-eluted fraction (0% HCW), the 35% ethanol-eluted fraction (35% HCW), and the 50% ethanol-eluted fraction (50% HCW).

### Animal experiments on the antidepressant effects of 35% HCW (The first antidepressant experiment)

Ninety male ICR mice (weight 18.0–22.0 g) were purchased from Hunan Silaike Jingda Experimental Animal Co., Ltd. The mice were housed at the Hunan Provincial Center for Drug Safety Evaluation [License No. SYXK (Xiang) 2015-0016]. Ten mice were randomly selected as the normal control group. The remaining mice were individually housed and subjected directly to a 28-day chronic unpredictable mild stress (CUMS) modeling protocol^12^. After modeling, based on the results of the sucrose preference test and body weight, the mice were assigned to ten groups, which included normal control, model control, fluoxetine hydrochloride(5.2 mg/kg/day), and three doses of 35% HCW and 50% HCW (7.5, 15, 30 mg/kg/day) groups. The vehicle for the intragastric administration was distilled water. All groups were administered the treatment via the same intragastric gavage method. The normal control and model control groups were orally administered distilled water (0.4 mL) daily, while the other groups received corresponding drug solutions of the same volume by oral gavage for 35 consecutive days. During the administration period, mice continued to receive CUMS modeling treatment after each gavage. After the last administration, behavioral tests were conducted on the mice, including the sucrose preference test (SPT), tail suspension test (TST), and novelty-suppressed feeding test (NSFT). All experiments and procedures were approved by the Biomedical Ethics Committee of Hunan Agricultural University (2025-216).

### Collection of the mouse cerebral cortex, hippocampus, and intestinal contents

Following the completion of behavioral tests, the mice were anesthetized using ether and euthanized by cervical dislocation. Under sterile conditions, brain tissues were immediately excised from the experimental mice. The cerebral cortex and hippocampus tissues were then carefully dissected. Once harvested, these tissues were promptly placed into sterile cryovials on ice packs for preservation. Subsequently, intestinal segments were excised, and intestinal contents were collected. The individual sampling amount per specimen was 200–500 mg per tube. The samples were placed into liquid nitrogen for rapid freezing. All samples were stored in a −80 °C freezer pending further analysis.

### Quantification of monoamine neurotransmitters, BDNF, inflammatory cytokines, oxidative stress factors in the mouse brain, and 16S rRNA sequencing of gut microbiota

The levels of monoamine neurotransmitters (including DA, NE, and 5-HT), BDNF, inflammatory cytokines (including IL-6 and TNF-α), and oxidative stress factors (including SOD enzyme and MDA) in the mouse cerebral cortex and hippocampus were measured using ELISA kits. All procedures were strictly performed in accordance with the operating instructions of the respective ELISA kits. A standard curve was plotted with the standard concentration on the *x*-axis and the OD value on the *y*-axis. The regression equation was derived, and sample concentrations were calculated by substituting the sample OD values into the equation. The 16S rRNA sequencing for intestinal microbiota analysis was conducted in collaboration with Shanghai Majorbio Bio-pharm Technology Co., Ltd., which performed the sequencing and subsequent analysis of the mouse intestinal 16S rRNA.

The linear regression equation fitted to the BDNF standard curve in the cerebral cortex was determined as *y* = 411.63*x*^2^ + 789.06*x*-19.617 (*R*^2^ = 0.9993). The fitted linear regression equation for the BDNF standard curve in the hippocampus was determined as *y* = 485.25*x*^2^ + 853.78*x*-19.601 (*R*^2^ = 0.9996).

The linear regression equation fitted to the NE standard curve in the cerebral cortex was determined as *y* = 44.241*x*^2^ + 146.06*x*-4.642 (*R*^2^ = 0.9996). The linear regression equation fitted to the NE standard curve in the hippocampus was determined as *y* = 83.783*x*^2^ + 116.83*x*-1.9422 (*R*^2^ = 0.9995).

The linear regression equation fitted to the DA standard curve in the cerebral cortex was determined as *y* = 464.07*x*^2^ + 1377.3*x*-27.651 (*R*^2^ = 0.998), and the linear regression equation fitted to the DA standard curve in the hippocampus was determined as *y* = 570*x*^2^ + 1101.6*x*-28.497 (*R*^2^ = 0.9996).

The linear regression equation fitted to the 5-HT standard curve in the cerebral cortex was determined as *y* = 77.976*x*^2^ + 512.05*x*-34.646 (*R*^2^ = 0.9998), and the linear regression equation fitted to the 5-HT standard curve in the hippocampus was determined as *y* = 194.62*x*^2^ + 332.44*x*-8.0554 (*R*^2^ = 0.9996).

The linear regression equation fitted to the IL-6 standard curve in the cerebral cortex was determined as *y* = 48.546*x*^2^ + 163.92*x*-8.3111 (*R*^2^ = 0.9994), and the linear regression equation fitted to the IL-6 standard curve in the hippocampus was determined as *y* = 38.299*x*^2^ + 168.61*x*-9.3702 (*R*^2^ = 0.9991).

The linear regression equation fitted to the TNF-α standard curve in the cerebral cortex was determined as *y* = 77.502*x*^2^ + 191.49*x*-5.9298 (*R*^2^ = 0.9999), and the linear regression equation fitted to the TNF-α standard curve in the hippocampus was determined as *y* = 116.07*x*^2^ + 157.82*x*-3.5384 (*R*^2^ = 0.9997).

### HPLC-Q/TOF-MS analysis method for 35% HCW

Chromatography was performed using an Agilent 1290 HPLC system (Agilent Technologies, USA). X-aqua C18 column (150 × 2.1 mm, 2.8 μm, Acclaim Technologies Co., Ltd., China) was used as the chromatographic column. Mobile phase consisted of aqueous phase A (0.1% formic acid in water) and organic phase B (0.1% formic acid in acetonitrile). The gradient elution program was as follows: 0–30 min, 5–45% B; 30–40 min, 45–90% B; 40–45 min, 90–90% B. Column temperature was set to 30 °C and the flow rate was set to 0.3 mL/min. The injection volume was 5 μL and the detection wavelength was 300 nm.

Mass spectrometric experiments were performed using a 6530 Q-TOF/MS accurate mass spectrometer (Agilent Technologies, USA) in negative ionization mode. The condition of Q-TOF-MS was optimized as follows: Negative ion mode (*m/z* 100–1000); sheath gas temperature: 350 °C; sheath gas flow: 11.0 L/min; drying gas temperature: 300 °C; nebulizer pressure: 45 psi; drying gas flow: 10 L/min; capillary voltage: 3500 V; fragmentor voltage: 175 V. Data acquisition software was Agilent MassHunter Acquisition Workstation Software (B.05.00). High-resolution tandem mass spectrometry (HRMS/MS) data were acquired by the target MS/MS mode with collision energies ranging from 10 to 80 eV. Reference solutions (*m/z* 112.9855 and 966.0007) were used for continuous calibration of the time-of-flight mass spectrometer to achieve high-accuracy mass measurements.

### Isolation of compounds 1 and 2 from 35% HCW

A total of 2.0 g of 35% HCW was ultrasonically dissolved in a 50% methanol solution and filtered through a 0.45 μm microporous membrane prior to use. The isolation of compounds **1** and **2** was primarily performed using an Agilent 1260 preparative liquid chromatography system. The specific separation conditions were as follows: Agilent ZORBAX SB-C18 column (4.6 × 250 mm, 5 μm); Mobile phase: 0.1% formic acid in water (A) and 0.1% formic acid in acetonitrile (B); Gradient elution: 0–10 min, 5–15%B; 10–15 min, 15–25%B; 15–20 min, 25–30%B; 20–30 min, 30–90%B; Flow rate: 1.0 mL/min; Column temperature: 30 °C; Injection volume: 60 μL; Detection wavelength: 300 nm (Fig. S1). Ultimately, compounds **1** (12.7 mg) and 2 (24.6 mg) were isolated, and their structures were determined based on nuclear magnetic resonance (NMR) spectroscopy and HRMS.

### Synthesis of compounds 1 and 2

#### Synthesis of Cp1

Quinic acid (5 g, 26 mmol) and p-toluenesulfonic acid monohydrate (495 mg, 2.6 mmol) were dissolved in 50 mL of toluene, and 12 mL of N,N-dimethylformamide (DMF) was added. The reaction mixture was heated under reflux in a 150 °C oil bath for 18 h. After cooling to room temperature, 100 mL of water was poured into the mixture. The layers were separated, and the aqueous layer was extracted multiple times with n-Butanol. The combined organic extracts were concentrated under reduced pressure. The target Cp**1** was obtained as a white solid via column chromatography, and the yield was approximately 82%. The 1H-NMR and 13C-NMR data see Fig. S2.

#### Synthesis of Cp2 and Cp3 mixture

Cp**1** (6 g, 34.50 mmol) was dissolved in N,N-dimethylformamide (65 mL). Under a 0 °C condition, imidazole (128.52 mmol) and tert-butyldimethylsilyl chloride (6 g, 40.00 mmol) were added, and the reaction proceeded for 30 min, followed by stirring at room temperature for 1 h. The reaction mixture was poured into 100 mL of water and extracted three times with 80 mL of ethyl acetate. The combined organic layers were washed three times with 200 mL of water, dried over anhydrous sodium sulfate, and concentrated under reduced pressure. Separation and purification yielded a white solid, which was determined to be a mixture of the target products Cp**2** and Cp**3** via NMR spectroscopic analysis. The yield was approximately 62%. The 1H-NMR and 13C-NMR data see Fig. S3.

#### Synthesis of Cp3

Cp**1** (3.54 g, 20.3 mmol) was dissolved in DMF (40 mL). DMAP (350 mg, 2.86 mmol), tetrabutylammonium iodide (378 mg, 1.02 mmol), and triethylamine (3.4 mL, 24.40 mmol) were added sequentially, followed by tert-butyldimethylsilyl chloride (3.8 g, 25.00 mmol). The solution was stirred at –10 °C for 1 h, then allowed to warm to room temperature and stirred for an additional 5 h. After completion of the reaction, the mixture was diluted with ethyl acetate (200 mL), yielding a yellow solution containing a large amount of white precipitate. This mixture was filtered using a suction flask. The filtrate was washed three times sequentially with 150 mL of 1 M hydrochloric acid and 150 mL of saturated sodium chloride solution. The organic layer was dried over anhydrous sodium sulfate, filtered, and concentrated under vacuum. Purification by column chromatography yielded a white solid, which was identified as the target product Cp**3** with a characteristic odor. The yield was approximately 65%. The 1H-NMR and 13C-NMR data see Fig. S4.

#### Synthesis of Cp4

Dimethylaminopyridine (DMAP) was dissolved in 20 mL of pyridine, followed by the addition of 4-hydroxycinnamic acid (10 g, 60.92 mmol) to form a suspension. After reacting at 0 °C for 30 min, acetic anhydride (9.32 g, 91.38 mmol) was added, and the mixture was stirred at room temperature for 3 h. Upon completion of the reaction, the mixture was poured into 100 mL of ice water, and white precipitate was gradually formed by the slow addition of HCl. The precipitate was collected by suction filtration and washed slowly with distilled water. The obtained white solid was concentrated under reduced pressure to afford the target product Cp**4** in approximately 90% yield. The 1H-NMR and 13C-NMR data see Fig. S5.

#### Synthesis of compound 1

Cp**4** (0.83 g, 4 mmol) and a mixture of Cp**2** and Cp**3** (576 mg, 2 mmol) were successively dissolved in dichloromethane (80 mL). The solution was stirred at 0 °C until fully dissolved. Then, DMAP (244 mg, 2 mmol) and N,N’-dicyclohexyl- carbodiimide (DCC) catalyst (1.24 g, 2 mmol) were added. After stirring for 10 minutes, the reaction mixture was warmed to room temperature and allowed to react for 24 h. Upon completion, the solvent was removed, and a crude white solid was obtained through simple purification via column chromatography. The white solid (300 mg) was dissolved in tetrahydrofuran (3 mL), and 2 M hydrochloric acid (9 mL) was added. The mixture was stirred at 40 °C for 96 h. After the reaction, an excess of saturated sodium chloride solution was added, followed by extraction with 90 mL of ethyl acetate three times. The combined organic layers were dried over anhydrous sodium sulfate, and the solvent was removed under reduced pressure. Compound **1** (3-*O*-p-coumaroylquinic acid) was obtained via high-speed counter-current chromatography with a yield of approximately 40%.

#### Synthesis of compound 2

Cp**3** (576 mg, 2 mmol) and Cp**4** (0.83 g, 4 mmol) were successively dissolved in dichloromethane (80 mL). The solution was stirred at 0 °C until fully dissolved. Subsequently, DMAP (244 mg, 2 mmol) and DCC catalyst (1.24 g, 2 mmol) were added. After stirring for 10 min, the reaction mixture was warmed to room temperature and reacted for 24 h. Upon completion of the reaction, the solvent was removed, and a white solid was obtained through simple purification via column chromatography. This white solid (300 mg) was dissolved in tetrahydrofuran (3 mL), and 2 M hydrochloric acid (9 mL) was added. The mixture was stirred at room temperature for 48 h. After the reaction, an excess of saturated sodium chloride solution was added to the mixture, which was then extracted three times with ethyl acetate (30 mL each time). The combined organic extracts were dried over anhydrous sodium sulfate, and the solvent was removed under reduced pressure. Purification using high-speed countercurrent chromatography yielded Compound **2** (4-*O*-p-coumaroylquinic acid) with a yield of approximately 45%.

### Evaluation of antidepressant activity for compounds 1 and 2 (The second antidepressant experiment)

A total of 50 male ICR mice, weighing 18.0–22.0 g, were provided by Hunan Silaike Jingda Laboratory Animal Co., Ltd. (No.: SCXK (Xiang) 2019-0004). Ten mice were assigned to the normal control group, while the remaining mice were subjected to CUMS modeling. The stress paradigms included: fasting and water deprivation (24 h), ice-water bath at 4 °C (5 min), foot shock (1 min, 2 mA), noise exposure (6 h), tail clamping (2 min), wet cage (24 h), day-night reversal, and restraint (12 h, with food and water deprivation during restraint). After 42 days of modeling, an SPT was conducted. Based on the SPT results and body weight, the mice were randomly divided into 5 groups of ten, including normal control, model control, fluoxetine hydrochloride control, 35% HCW(30 mg/kg), and compound **1** and **2** (30 mg/kg each) groups. All groups received intragastric administration via the same method, and pure water was used as the vehicle for all drug solutions. The normal and model control groups were administered 0.4 mL of distilled water daily by gavage, while the other groups received their respective drug solutions at a volume of 0.4 mL. The treatment continued for 35 consecutive days, and the CUMS was maintained after each administration. After the last administration, behavioral assessments, including SPT, TST, and FST, were performed on the mice. All experiments and procedures were approved by the Biomedical Ethics Committee of Hunan Agricultural University (2025-216).

### In vivo metabolism study of compound 1 based on the CA/Dopa/DA/NE pathway

After completing the behavioral tests in mice, the final dose was administered, and this time point was designated as 0 h. At 2.0 h, blood collection was employed via eyeball enucleation. The blood samples were allowed to stand at room temperature for 1 h, then centrifuged at 4 °C at 3000 rpm/min for 30 min. The supernatant was collected for the detection of Dopa in mouse blood. Following orbital blood collection, the mice were anesthetized with ether and sacrificed. The intestinal contents were collected and evenly divided into two portions. One portion was used for LC-MS analysis of intestinal metabolites (including 4-hydroxycinnamic acid, tyrosine, and Dopa). The other portion was used to analyze the gut microbiota based on 16S rRNA gene sequencing technology. Simultaneously, the mouse cerebral cortex and hippocampus were dissected on ice, and each was divided into two portions. One portion was used to measure the levels of inflammatory factors (including IL-6 and TNF-α), monoamine neurotransmitters (including DA, NE, and 5-HT), and BDNF using ELISA kits. The other portion of the cerebral cortex was homogenized in 20 volumes of PBS. The homogenate was centrifuged, and the supernatant was collected to determine the levels of Dopa, DA, and NE using mass spectrometry.

The mass spectrometry detection method for the CA/Dopa/DA/NE pathway is as follows: An Agilent 1290 HPLC system coupled with a 6530 Q-TOF-MS mass spectrometer was employed, using an Agilent ZORBAX SB-C18 column (4.6 × 250 mm, 5 µm). The mobile phase consisted of water phase A (0.1% formic acid in water) and organic phase B (0.1% formic acid in acetonitrile). The gradient elution program was: 0–30 min, 5–45% B; 30–40 min, 45–90% B; 40–50 min, 90–95% B. The column temperature was set at 30 °C. The flow rate was 0.3 mL/min, and the injection volume was 5 µL. The analysis was performed in negative ion mode, with a scan range of *m/z* 100–1000. The sheath gas temperature was 350 °C, with a flow rate of 11.0 L/min. The drying gas temperature was 300 °C, with a pressure of 45 psi and a flow rate of 10 L/min. The capillary voltage was 3500 V, and the fragmentor voltage was 175 V.

### Determination of biochemical parameters in the mouse brain and intestinal microbiota

The levels of monoamine neurotransmitters, including DA, NE, and 5-HT, BDNF, and inflammatory cytokines, including IL-6 and TNF-α, in the mouse cerebral cortex and hippocampus were measured using enzyme-linked immunosorbent assay (ELISA) kits. All procedures were performed strictly in accordance with the respective kit manuals. The 16S rRNA sequencing for intestinal microbiota analysis was conducted in collaboration with Shanghai Majorbio Bio-pharm Technology Co., Ltd., which performed the sequencing and subsequent analysis of the mouse intestinal 16S rRNA.

The linear regression equation fitted to the BDNF standard curve in the cerebral cortex was determined as *y* = 0.0081*x*-0.0026 (*R*^2^ = 0.9961). The fitted linear regression equation for the BDNF standard curve in the hippocampus was determined as *y* = 0.0052*x* + 0.0852 (*R*^2^ = 0.9976).

The linear regression equation fitted to the 5-HT standard curve in the cerebral cortex was determined as *y* = 0.0228*x* + 0.0702 (*R*^2^ = 0.9983). The fitted linear regression equation for the 5-HT standard curve in the hippocampus was determined as *y* = 0.1246*x* + 0.0235 (*R*^2^ = 0.9969).

The linear regression equation fitted to the DA standard curve in the cerebral cortex was determined as *y* = 0.0502*x* + 0.0564 (*R*^2^ = 0.9994). The fitted linear regression equation for the DA standard curve in the hippocampus was determined as *y* = 0.0379*x* + 0.0254 (*R*^2^ = 0.9981).

The linear regression equation fitted to the NE standard curve in the cerebral cortex was determined as *y* = 0.5241*x* + 0.0594 (*R*^2^ = 0.9962). The fitted linear regression equation for the NE standard curve in the hippocampus was determined as *y* = 0.0062*x* + 0.0429 (*R*^2^ = 0.9995).

The four-parameter logistic curve fitting equation for IL-6 was determined as *y* = (3.87597-0.07515)/[1 + (*x*/1167.27370)^(-1.17219)] + 0.07515 (*R*^2^ = 0.9991).

The four-parameter logistic curve fitting equation for TNF-α was determined as *y* = (10.34837-0.07010)/[1 + (*x*/3907.96593)^(-0.79462)] + 0.07010 (*R*^2^ = 0.9996).

### Evaluation of the antidepressant activity of compound 1 + H-89 and compound 2 + H-89 (The third antidepressant experiment)

Seventy male ICR mice (18.0-22.0 g) were purchased from Hunan Silaike Jingda Experimental Animal Co., Ltd. (License No.: SCXK (Xiang) 2019-0004). After one week of acclimatization, 10 mice were randomly selected as the normal control group. The remaining mice were individually housed to establish a depression model using CUMS protocol. Following 42 days of modeling, based on SPT data and body weight, the mice were randomly divided into seven groups (*n* = 10) including normal control, model control, fluoxetine hydrochloride (5.2 mg/kg/d), compound **1** (30 mg/kg/d), compound **1** + H-89 (30 mg/kg/d + 5 mg/kg/d), compound **2** (30 mg/kg/d), and compound **2** + H-89 group (30 mg/kg/d + 5 mg/kg/d). Pure water was used as the vehicle for the intragastric administration solutions, while the solvent for the injection solutions consisted of 10% DMSO, 40% PEG300, 5% Tween-80, and 45% saline. The intragastric gavage and injection procedures were consistent across all groups, with the same dosage administered to each group. The normal and model control groups received 0.4 mL of distilled water daily via oral gavage, while the other groups received their respective drug solutions at a volume of 0.4 mL. Drug administration continued for 35 consecutive days, during which the CUMS procedure was maintained after each administration. Finally, after the last administration, behavioral tests, including the SPT, TST, and FST, were conducted. BDNF levels in the mouse cerebral cortex and hippocampus were measured according to the instructions provided with the respective ELISA kits. All experiments and procedures were approved by the Biomedical Ethics Committee of Hunan Agricultural University (2025-216).

The four-parameter logistic curve fitting equation for BDNF in the cerebral cortex was determined as *y* = (4.03507 − 0.10733)/[1 + (*x*/1429.33572)^(−1.11672)] + 0.10733 (*R*^2^ = 0.9985).

The four-parameter logistic curve fitting equation for BDNF in the hippocampus was determined as *y* = (4.07624 − 0.12624)/[1 + (*x*/1527.33474)^(−1.182100)] + 0.12624 (*R*^2^ = 0.9983).

### Protein determination method based on the PKA/CREB/BDNF pathway

After the completion of behavioral tests, the mice were euthanized by ether anesthesia. The cerebral cortex and hippocampus were collected from each mouse. For protein content determination, three cortical cortex and three hippocampal samples were taken from each group. Protein extraction was performed according to the manufacturer’s instructions. The western blotting protocol included SDS-PAGE electrophoresis, membrane transfer, immunoreaction, chemiluminescence detection, and gel image analysis. The expression levels of PKA, p-PKA, CREB, and p-CREB in the mouse cerebral cortex and hippocampus were detected and analyzed via Western Blot.

### Statistical methods

Statistical analyses were performed using SPSS 26.0, with the level of statistical significance set at *p* ≤ 0.05. Data were expressed as mean ± SD. Statistical analysis was performed using one-way ANOVA followed by Tukey’s/Dunnett’s multiple comparison test. A value of *p* < 0.05 was considered statistically significant. All experiments were conducted with 10 biological replicates and 2 technical replicates, and the final results were calculated from the average values. Results were expressed as mean ± standard deviation. All figures were plotted using GraphPad Prism software.

## Supplementary information

Supporting Material520

## Data Availability

The data that support the findings of this study are available from the corresponding author, Zhixing Qing, upon reasonable request.

## Code availability

The underlying code for this study is not publicly available but may be made available to qualified researchers on reasonable request from the corresponding author.
