# Supplementary material for: 3-O-p-coumaroylquinic acid and 4-O-p-coumaroylquinic acid from Hemerocallis citrina Baroni exert antidepressant effects via CA/Dopa/DA/NE and PKA/CREB/BDNF signaling pathways
Source: NPJ Sci Food. 2026 Jul 1;10:323. doi: 10.1038/s41538-026-00934-0 (PMC13686965; doi:10.1038/s41538-026-00934-0)
Supplement: Supplementary file 1 [file 41538_2026_934_MOESM1_ESM.pdf]

3-*O*-p-Coumaroylquinic Acid and 4-*O*-p-Coumaroylquinic Acid from *Hemerocallis citrina* Baroni exert Antidepressant Effects via CA/Dopa/DA/NE and PKA/CREB/BDNF Signaling Pathways

Faying Jiang[a#], Juhua Zhong[a#], Tian Ye[a#], Jinghong Liu[b#], Yishuo Zhu[a], Zhengyue Qiu[a], Lewei Zhao[a], Siyu Chen[a], Hongbao Chen[a], Haobo Wang[a], Lin Kang[a], Tonghe Yang[a], Shuai Li[a], Si Liu[a], Jianguo Zeng[a,d*], Zhixing Qing[a,c,d*]

[a]College of Veterinary Medicine, Hunan Agricultural University, Changsha, 410128, China;

[b]college of tea culture, Hunan Vocational College of Commerce, Changsha, 410128, China;

[c]Changsha Changxu Biotechnology Ltd., Changsha, 410128, China;

[d]Yuelushan Laboratory, Changsha, 410128, China

[#]These authors contributed equally to this work.

*

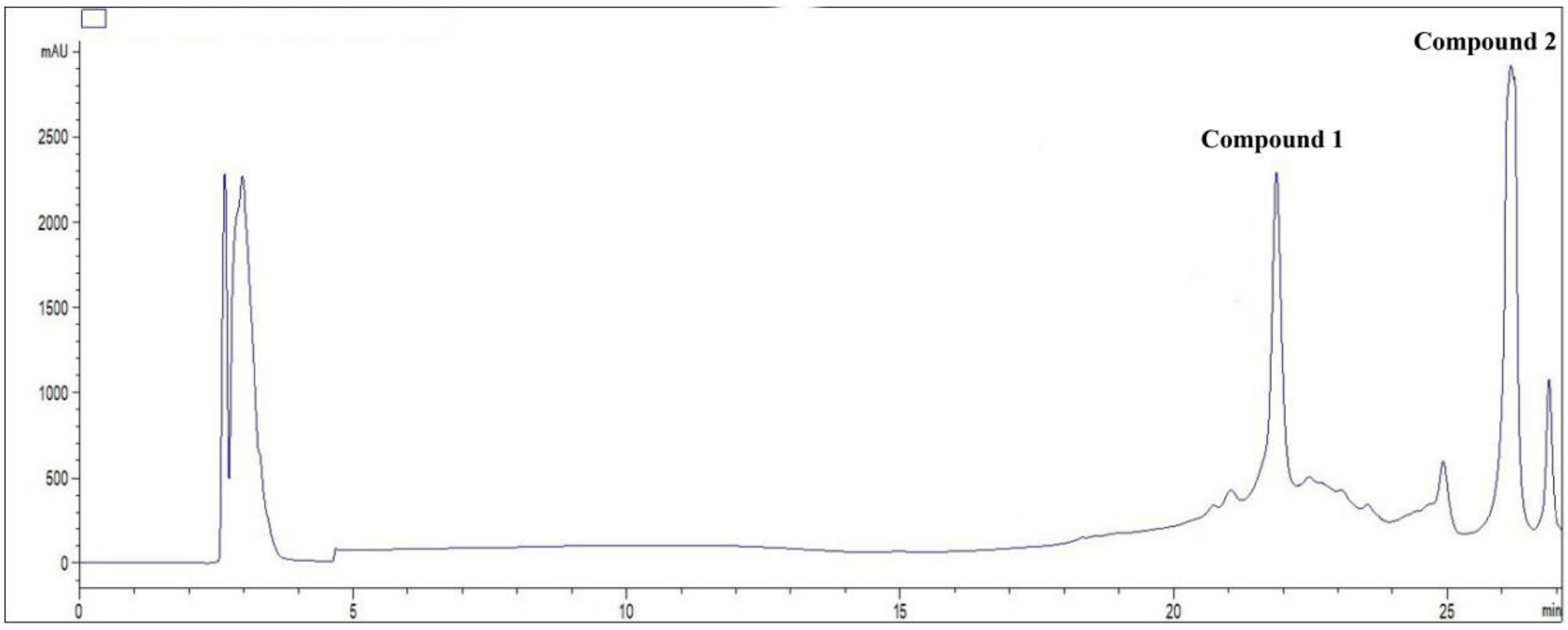

Fig. S1 The UV spectra of 35%HCW (300 nm) and the peaks of compounds **1** and **2**.

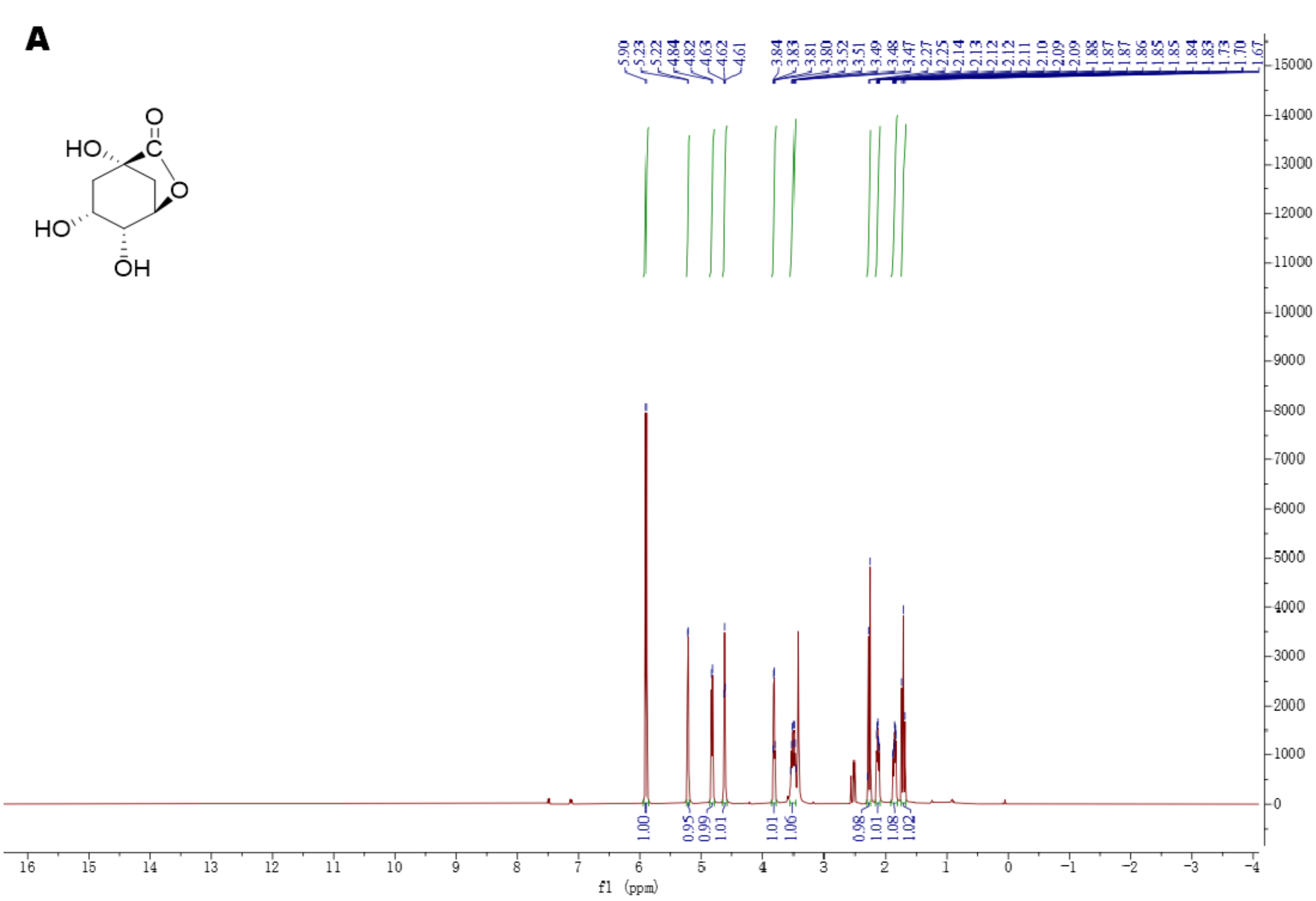

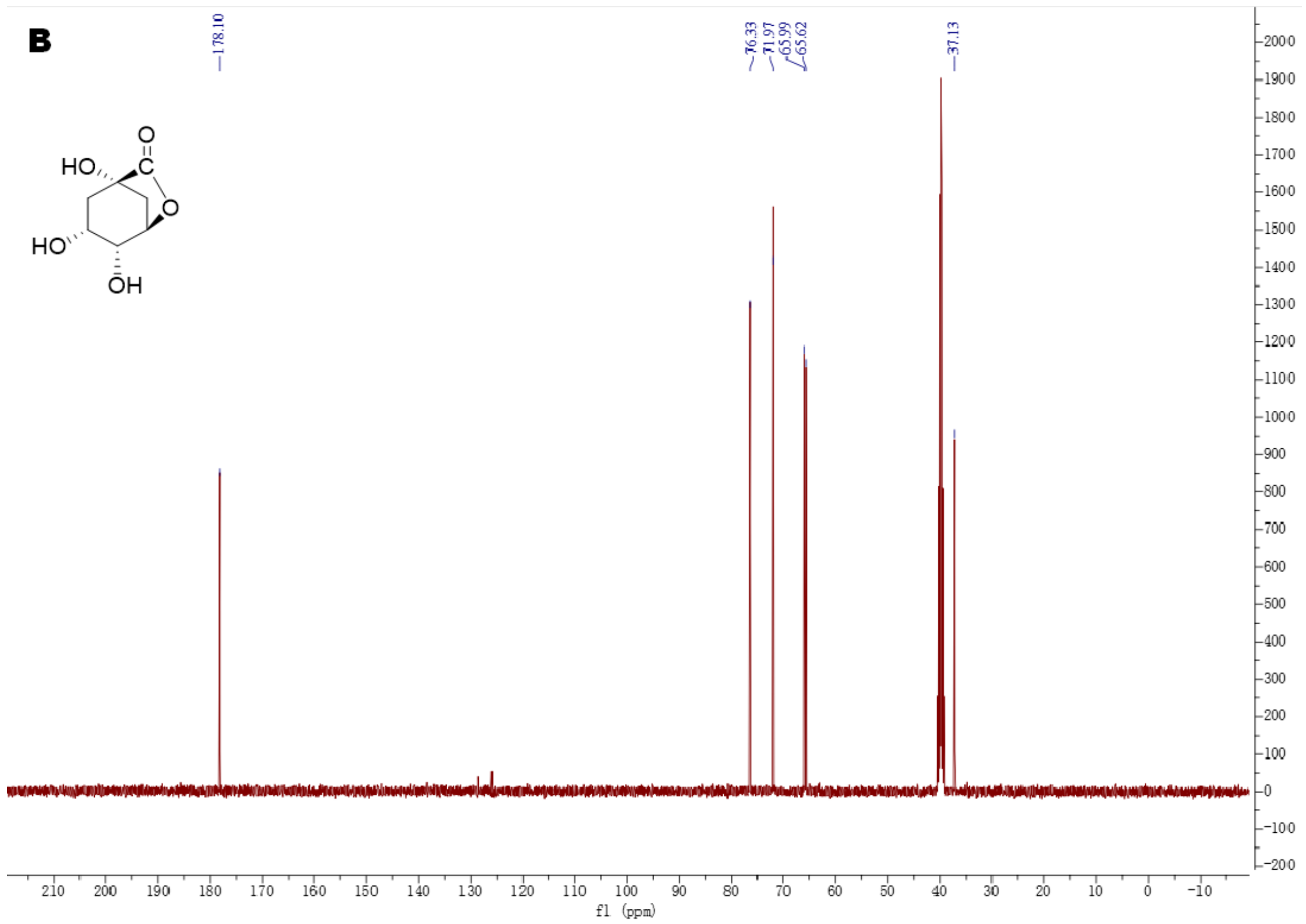

Fig. S2 The ^{1}H-NMR (A) and ^{13}C-NMR (B) data of Cp**1**.

White solid，HRMS (ESI$^-$)：calcd for $C_7H_9O_5^-$ $[M-H]^-$, theoretical *m/z* 173.0450; found *m/z* 173.0456. ^{1}H-NMR (400 MHz, DMSO-d6): δ 5.90 (s, 1H), 5.23 (d, *J*= 4 Hz, 1H), 4.84 (d, *J*= 8 Hz, 1H), 4.62 (t, *J*= 4 Hz, 1H), 3.82 (q, *J*= 4 Hz, 1H), 3.54-3.47 (m, 1H), 2.29-2.25 (m, 1H), 2.14-2.09 (m, 1H), 1.88-1.83 (m, 1H), 1.70 (t, *J*= 12 Hz, 1H). ^{13}C-NMR (100 MHz, DMSO-d6): δ 178.10, 76.33, 71.97, 65.99, 65.62, 37.13 (2×CH).

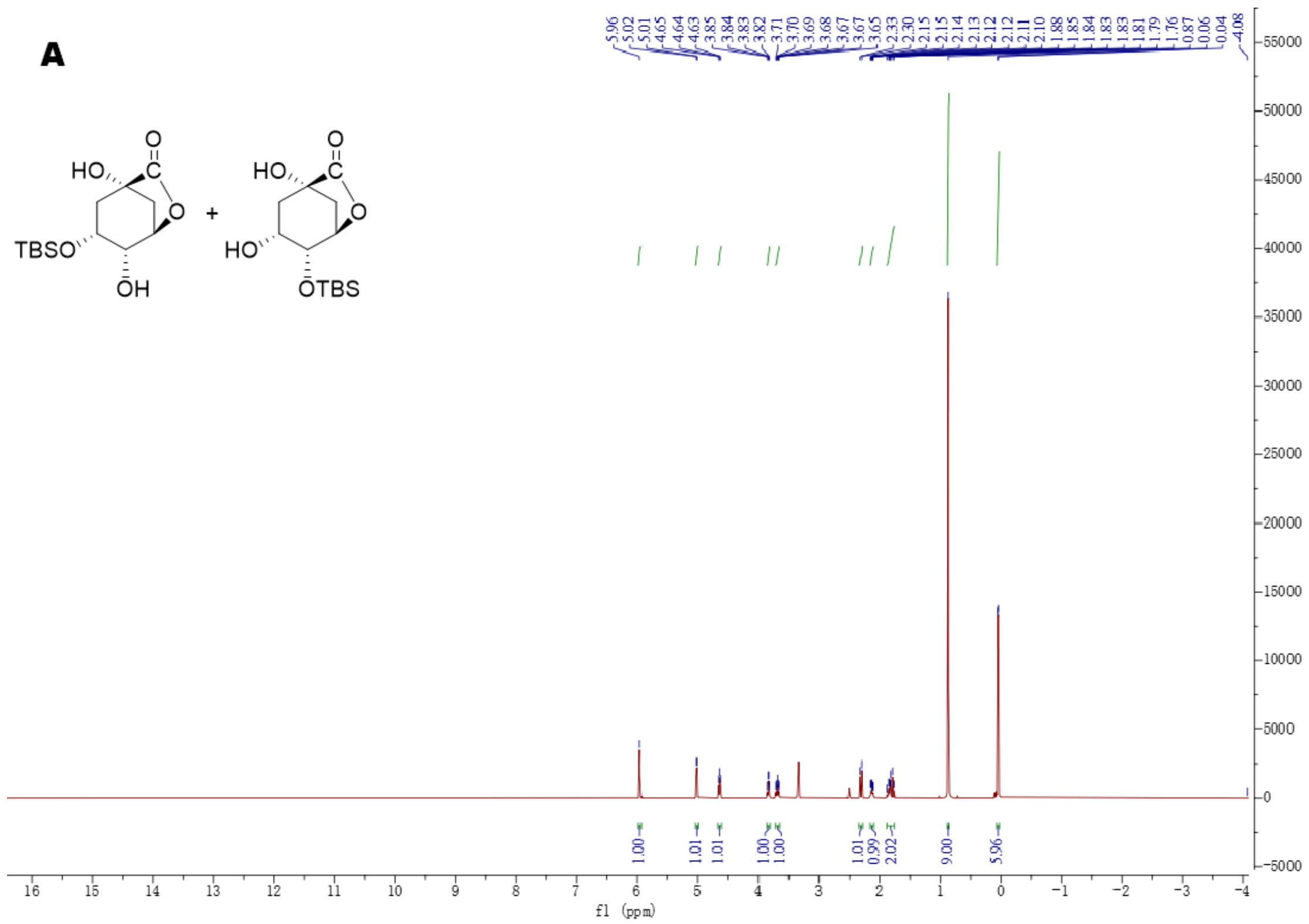

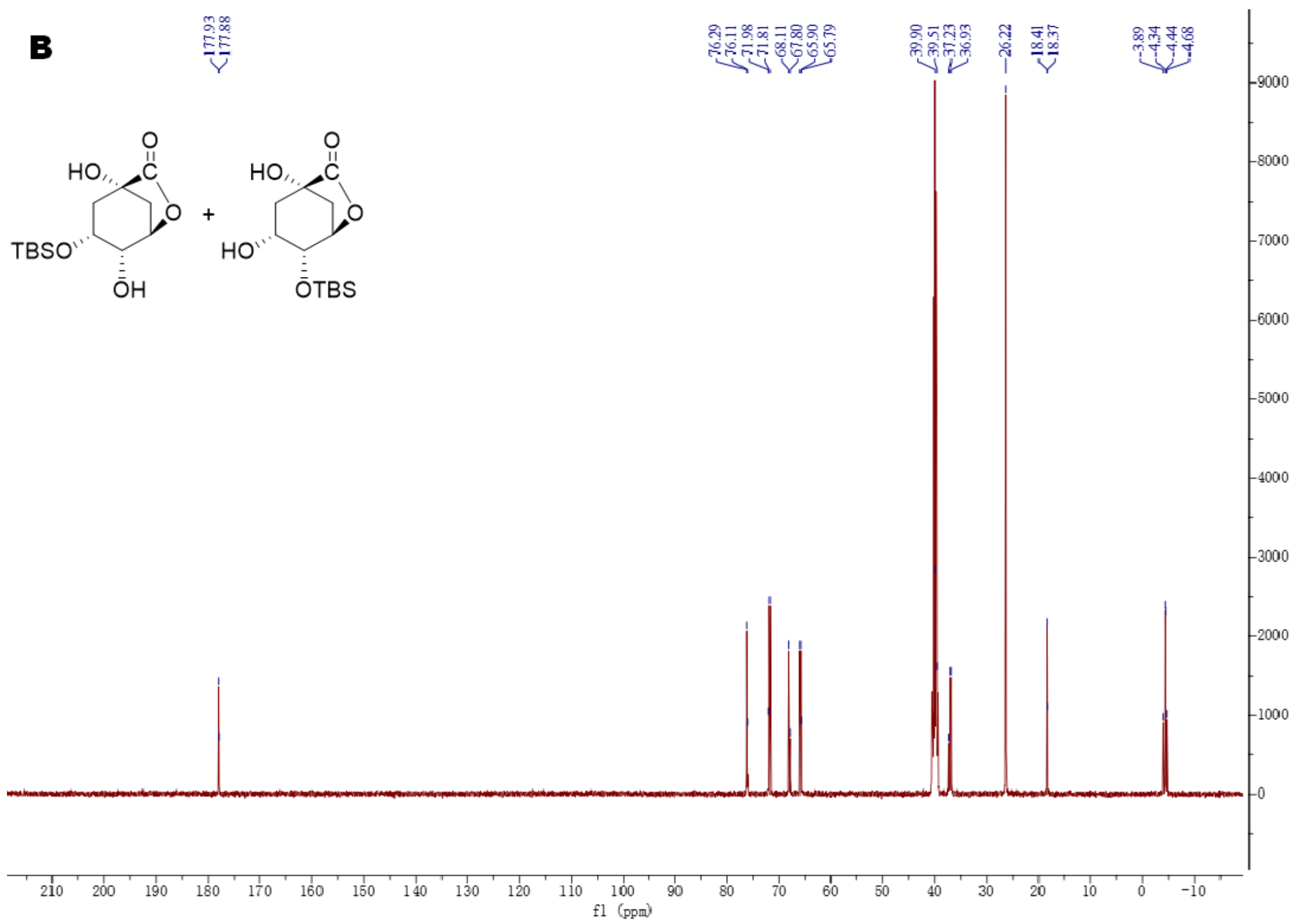

Fig. S3 The ^{1}H-NMR (A) and ^{13}C-NMR (B) data of the Cp**2**+Cp**3** mixture.

White solid，HRMS (ESI$^+$)：calcd for $C_{13}H_{25}O_5Si^+$ $[M+H]^+$,

theoretical *m/z* 289.1466; found *m/z* 289.1479. ^{1}H-NMR (400 MHz, DMSO-d6): δ 5.96 (s, 1H), 5.02 (d, *J*= 4 Hz, 1H), 4.64 (t, *J*= 4 Hz, 1H), 3.84 (q, *J*= 4 Hz, 1H), 3.71-3.65 (m, 1H), 2.32 (d, *J*= 12 Hz, 1H), 2.15-2.10 (m, 1H), 1.88-1.76 (m, 2H), 0.87 (s, 9H), 0.05 (d, *J*= 8 Hz, 6H). ^{13}C-NMR (100 MHz, DMSO-d6): δ 177.93, 177.88, 76.29, 76.11, 71.98, 71.81, 68.11, 67.80, 65.09, 65.79, 39.90, 39.51, 37.23, 36.93, 26.22(6×CH), 18.41, 18.37, -3.89, -4.34, -4.44, -4.68.

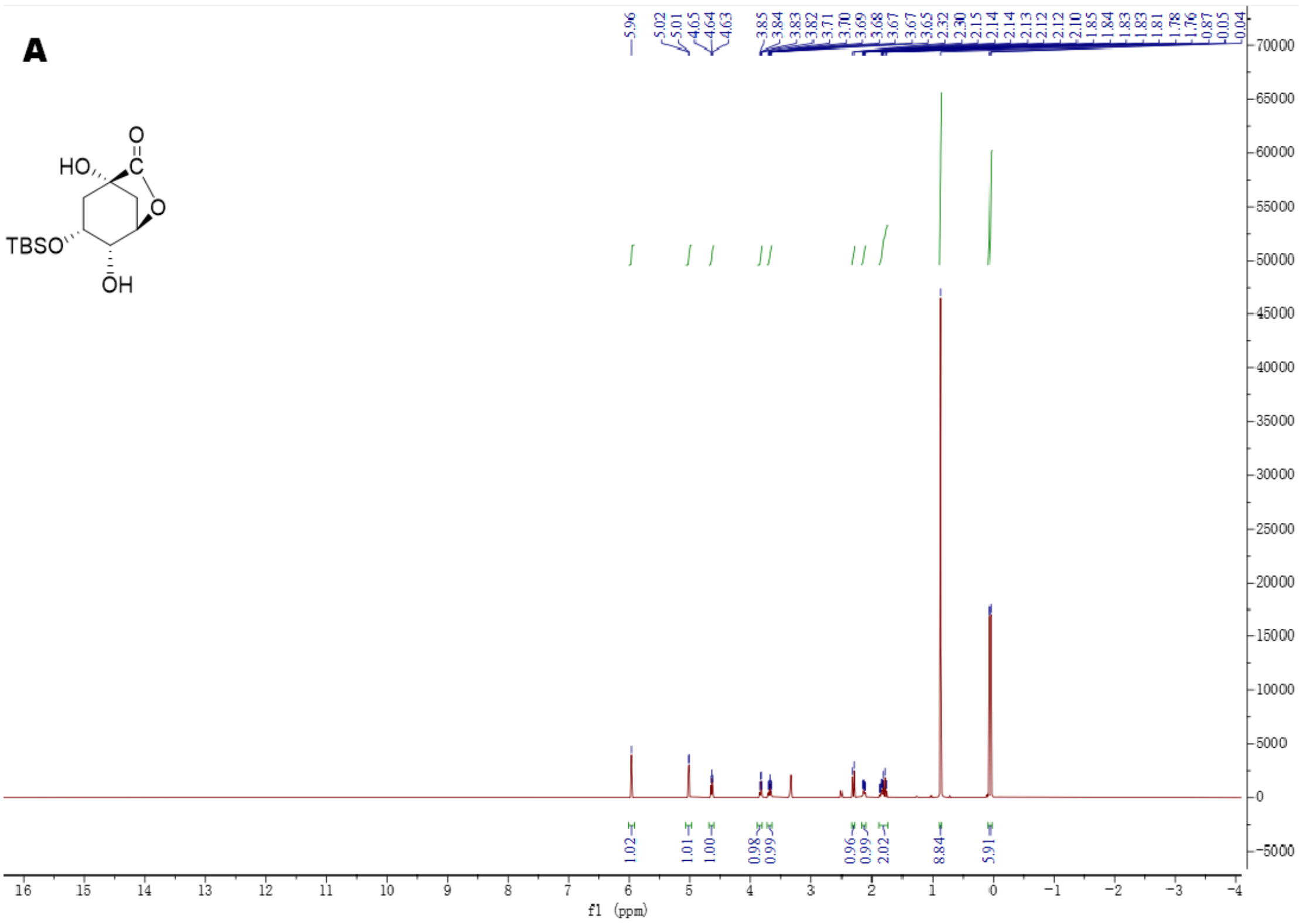

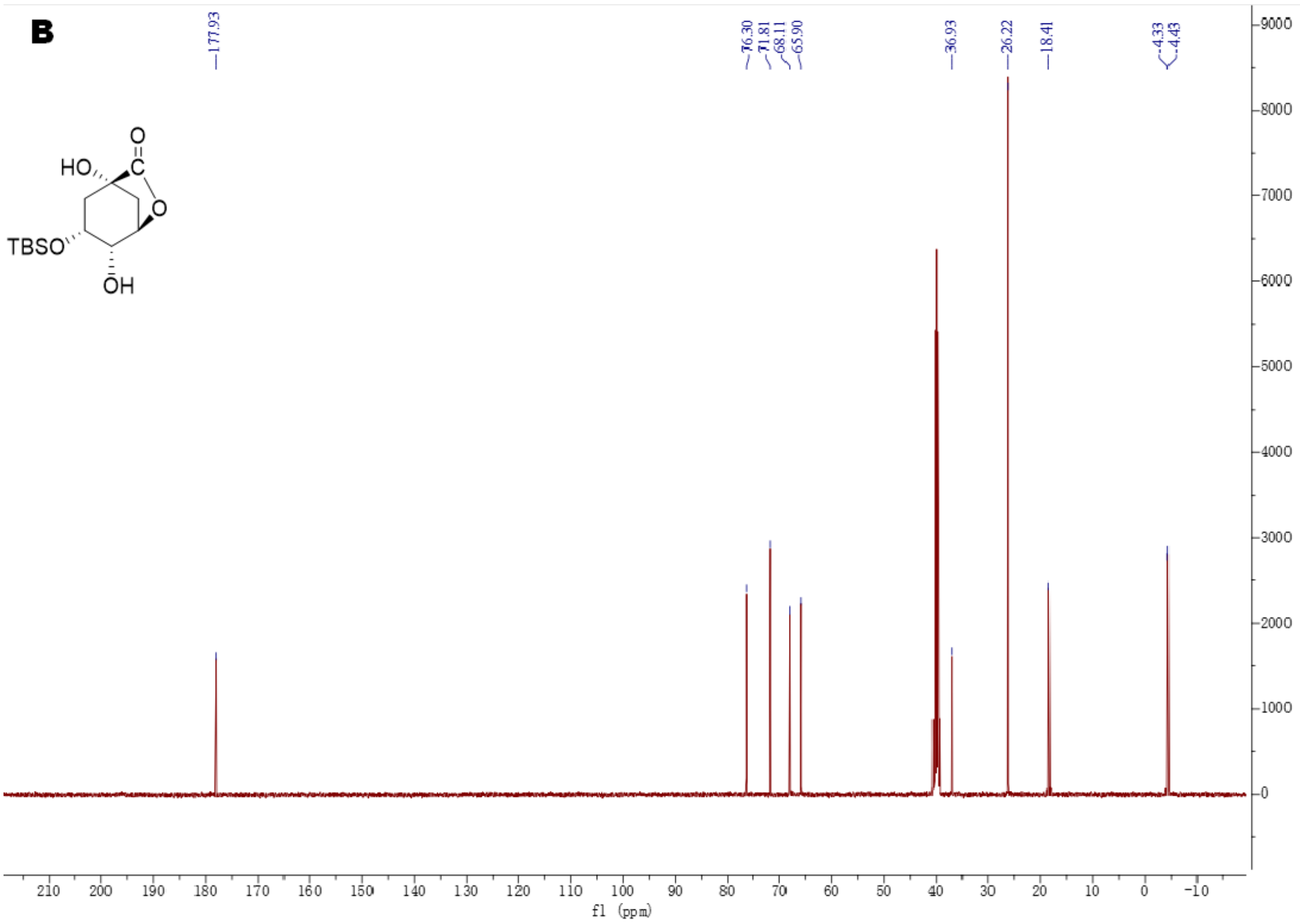

Fig. S4 The ^{1}H-NMR (A) and ^{13}C-NMR (B) data of Cp**3**.

White solid, HRMS (ESI^{+})： calcd for $C_{13}H_{25}O_{5}Si^{+}$ $[M+H]^{+}$, theoretical *m/z* 289.1466; found *m/z* 289.1479. ^{1}H-NMR (400 MHz, DMSO-d6): δ 5.96 (s, 1H), 5.02 (d, *J*= 4 Hz, 1H), 4.64 (t, *J*= 8 Hz, 1H), 3.85-3.82 (m, 1H), 3.71-3.65 (m, 1H), 2.32 (d, *J*= 8 Hz, 1H), 2.15-2.10 (m, 1H), 1.88-1.76 (m, 2H), 0.87 (s, 9H), 0.05 (d, *J*= 4 Hz, 6H). ^{13}C-NMR (100 MHz, DMSO-d6): δ 177.93, 76.30, 71.81, 68.11, 65.90, 39.55(3×CH), 36.93, 26.22(3×CH), 18.41, -4.33, -4.43.

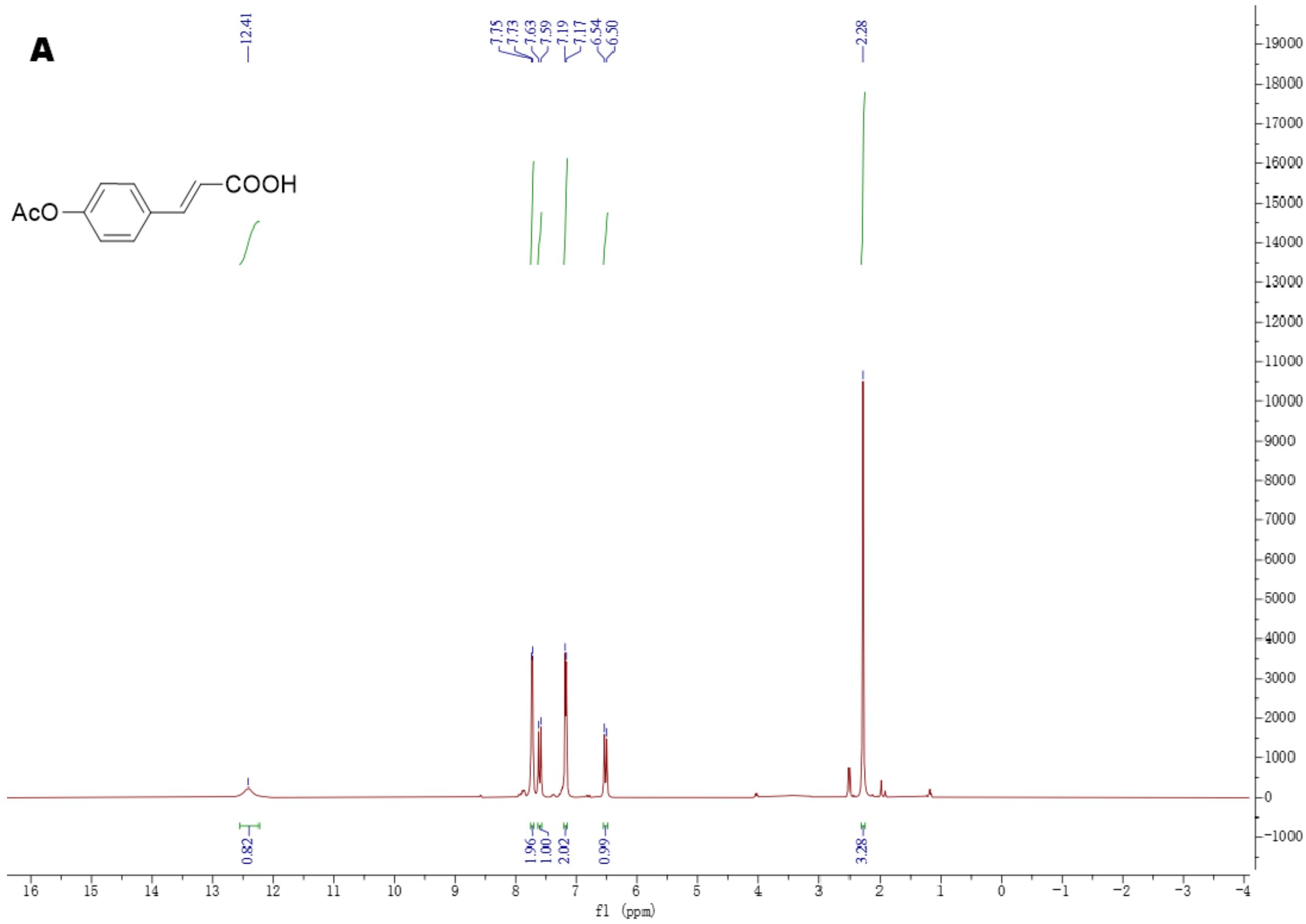

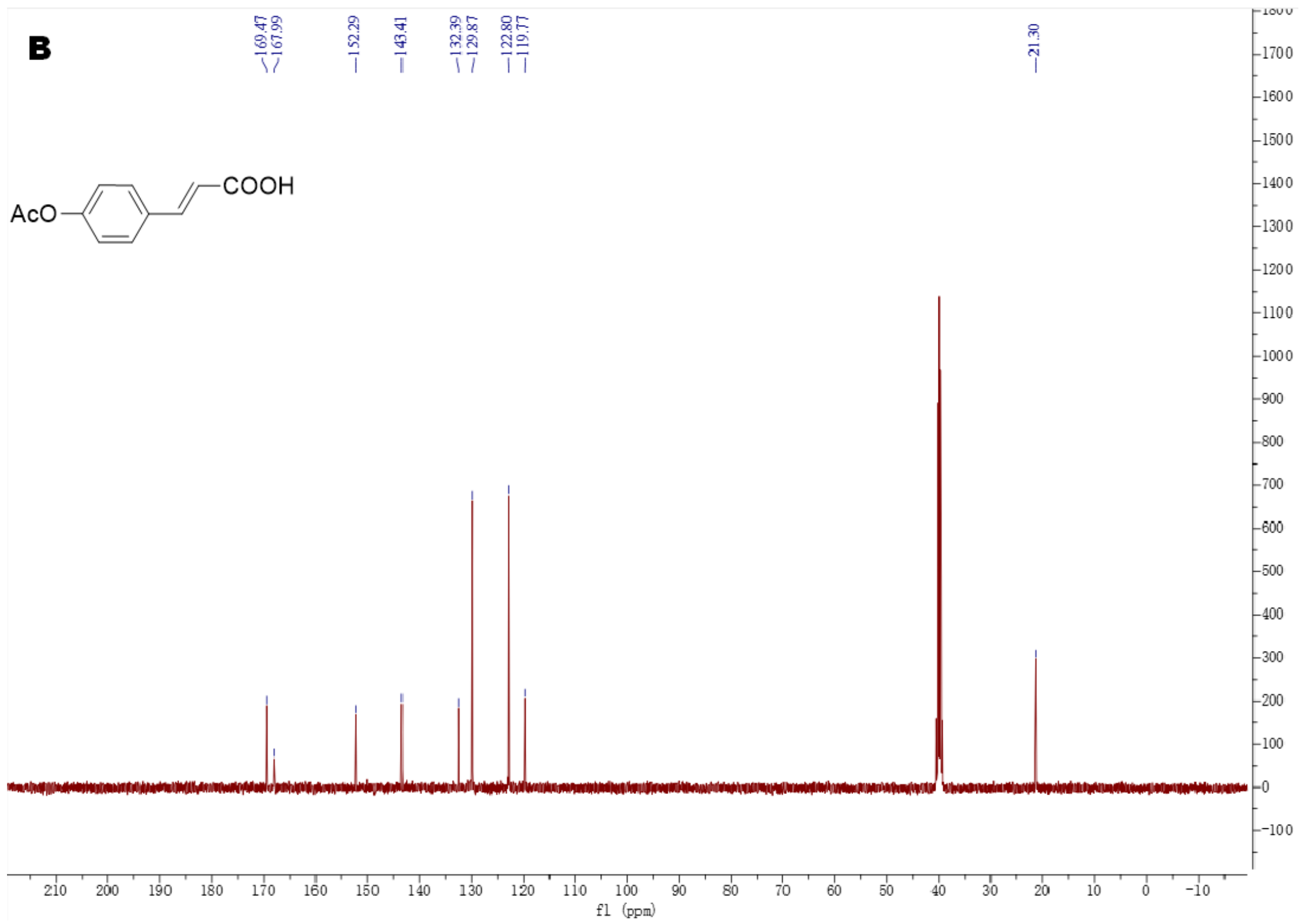

Fig. S5 The ^{1}H-NMR (A) and ^{13}C-NMR (B) data of Cp **4**.

White solid, HRMS (ESI^{+})：calcd for $C_{11}H_{11}O_4^{+}$ $[M+H]^{+}$,

theoretical *m/z* 207.0652; found *m/z* 207.0655. ^{1}H-NMR (400 MHz, DMSO-d6): δ 12.41 (s, 1H), 7.74 (d, *J*= 8 Hz, 2H), 7.63 (d, *J*= 16 Hz, 1H), 7.18 (d, *J*= 8 Hz, 2H), 6.52 (d, *J*= 16 Hz, 1H), 2.28 (s, 3H). ^{13}C-NMR (100 MHz, DMSO-d6): δ 169.47, 167.99, 152.29, 143.41, 132.39, 129.87 (2×CH), 122.80 (2×CH), 119.77, 21.30.

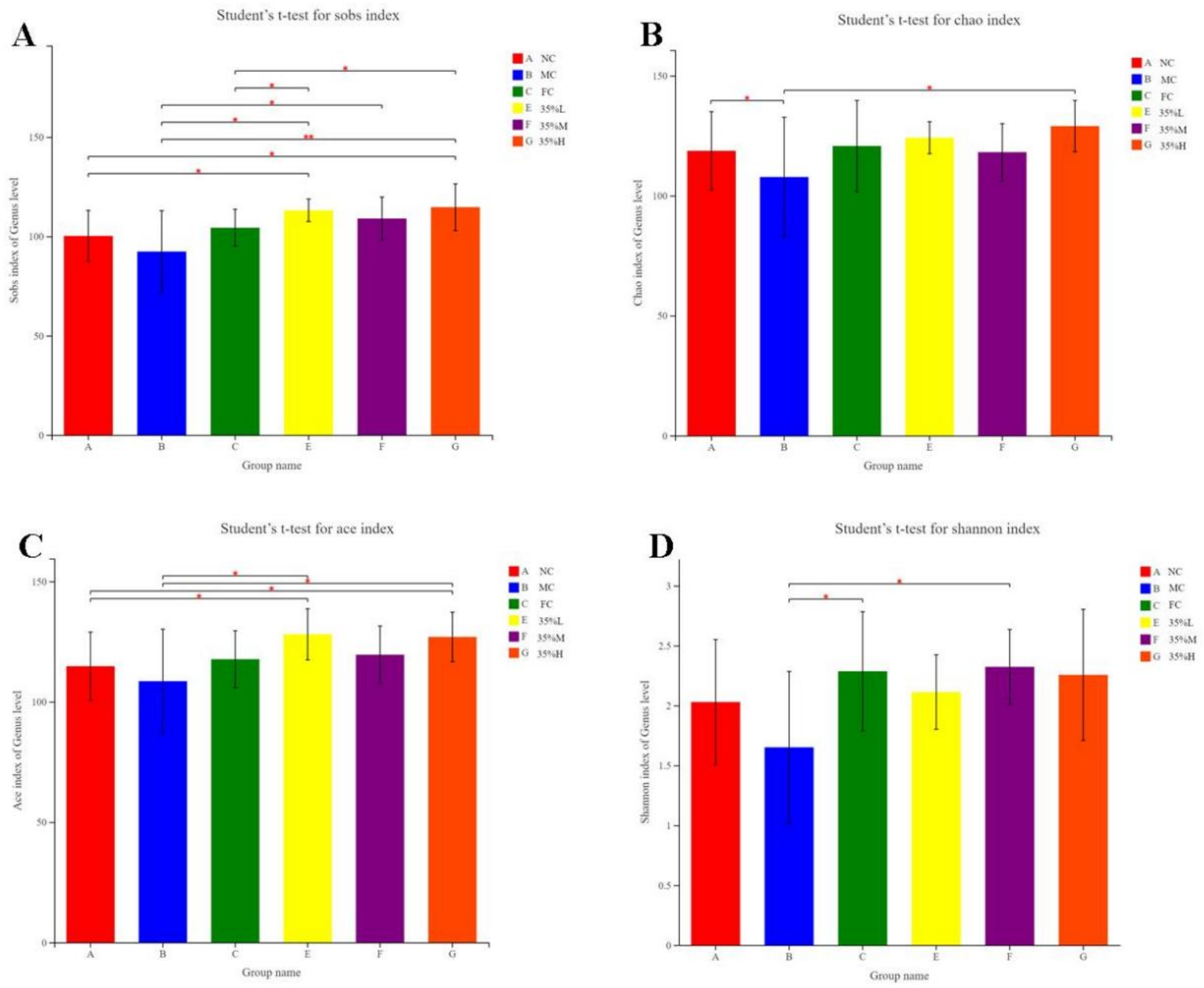

Fig. S6 The Sobs (A), Chao1(B), ACE(C), and Shannon index (D) of the gut microbiota in different groups.

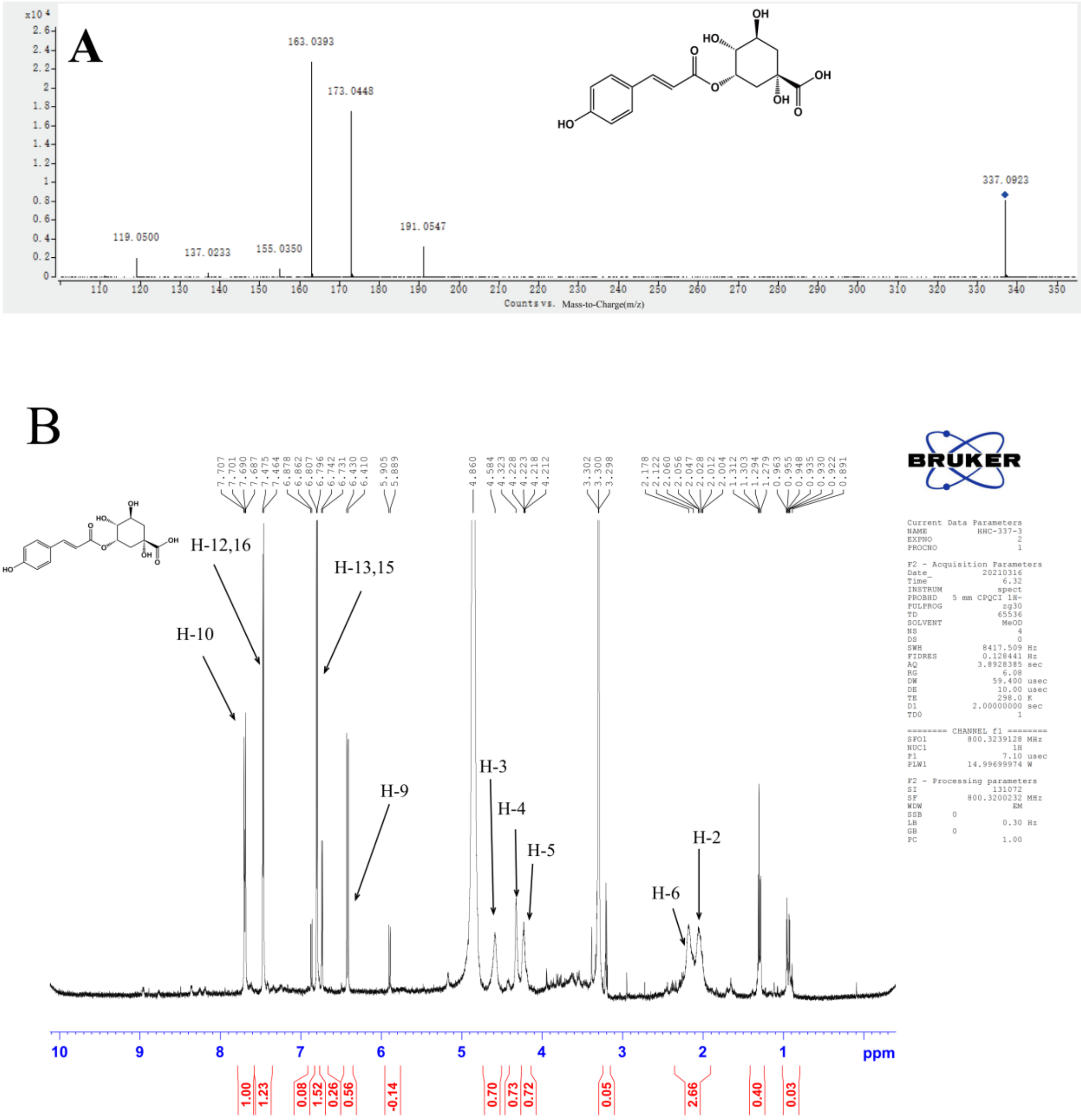

A
163.0393
173.0448
119.0500
137.0233
155.0350
191.0547
337.0923
Counts vs. Mass-to-Charge(m/z)
B
BRUKER
H-12,16
H-13,15
H-10
H-9
H-3
H-4
H-5
H-6
H-2
ppm

C

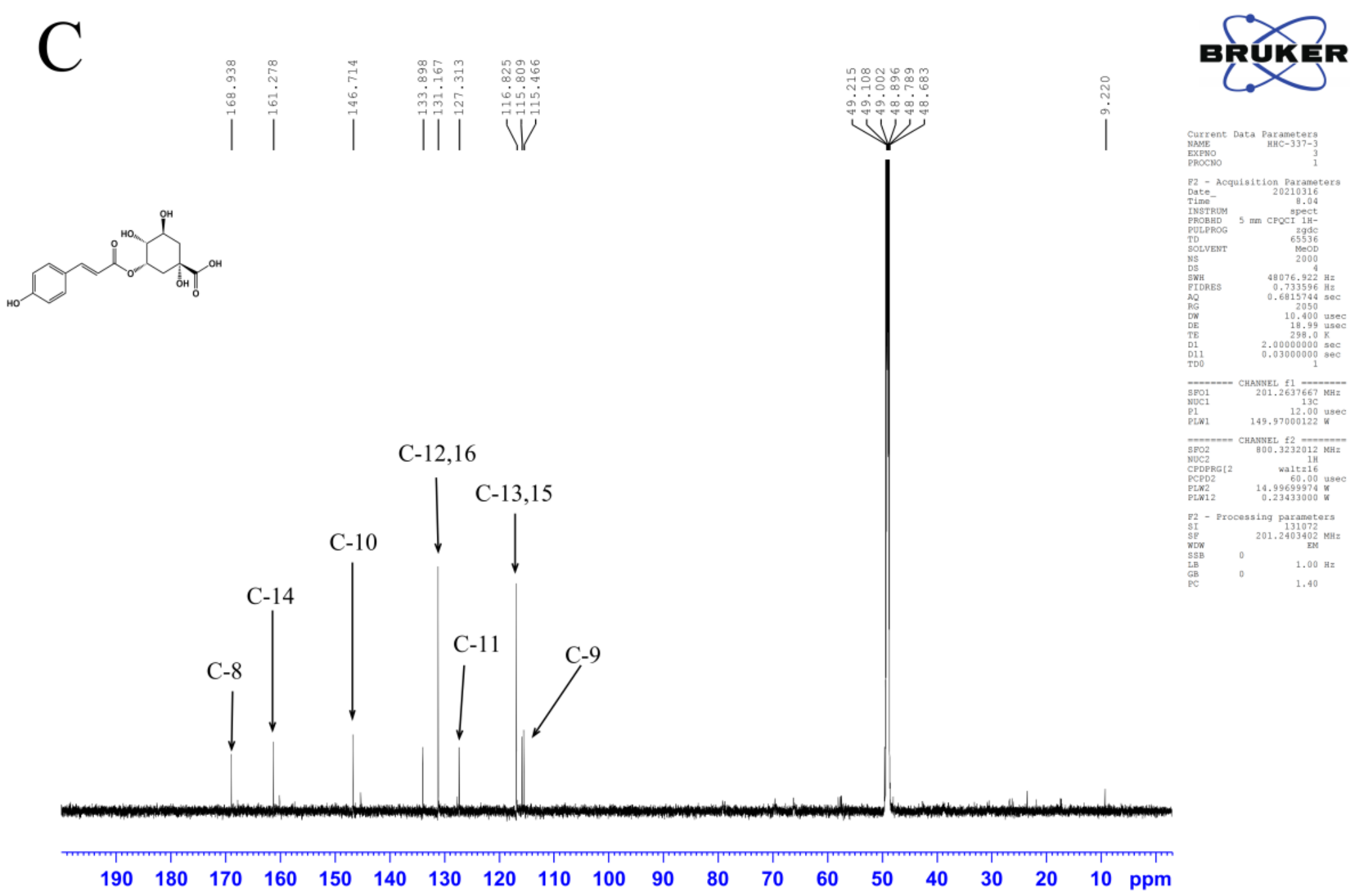
168.938
161.278
146.714
133.898
131.167
127.313
116.825
115.809
115.466
49.215
49.108
49.002
48.896
48.789
48.683
9.220
C-8
C-14
C-10
C-12,16
C-11
C-13,15
C-9
ppm
BRUKER

D

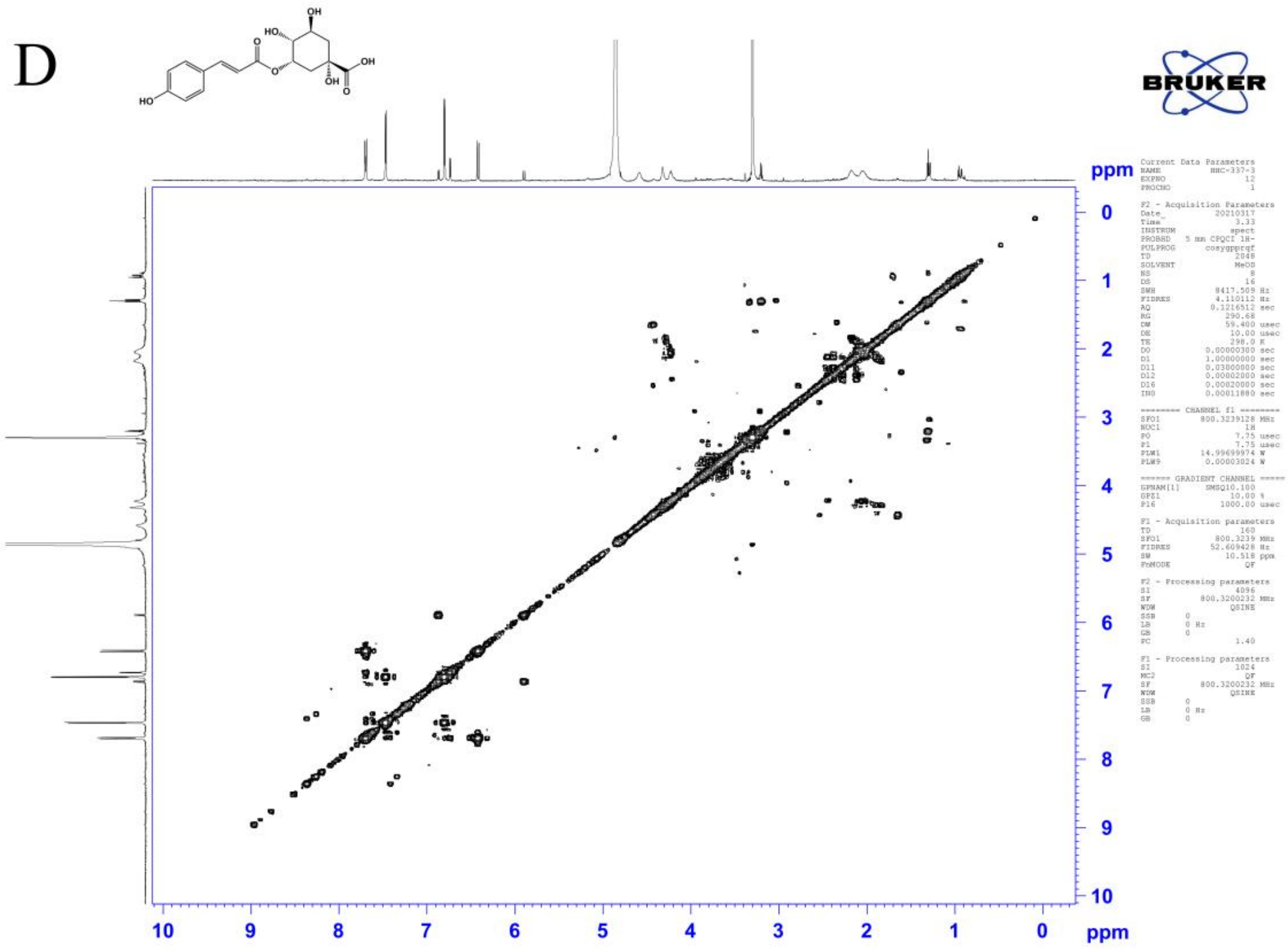
ppm
BRUKER

E

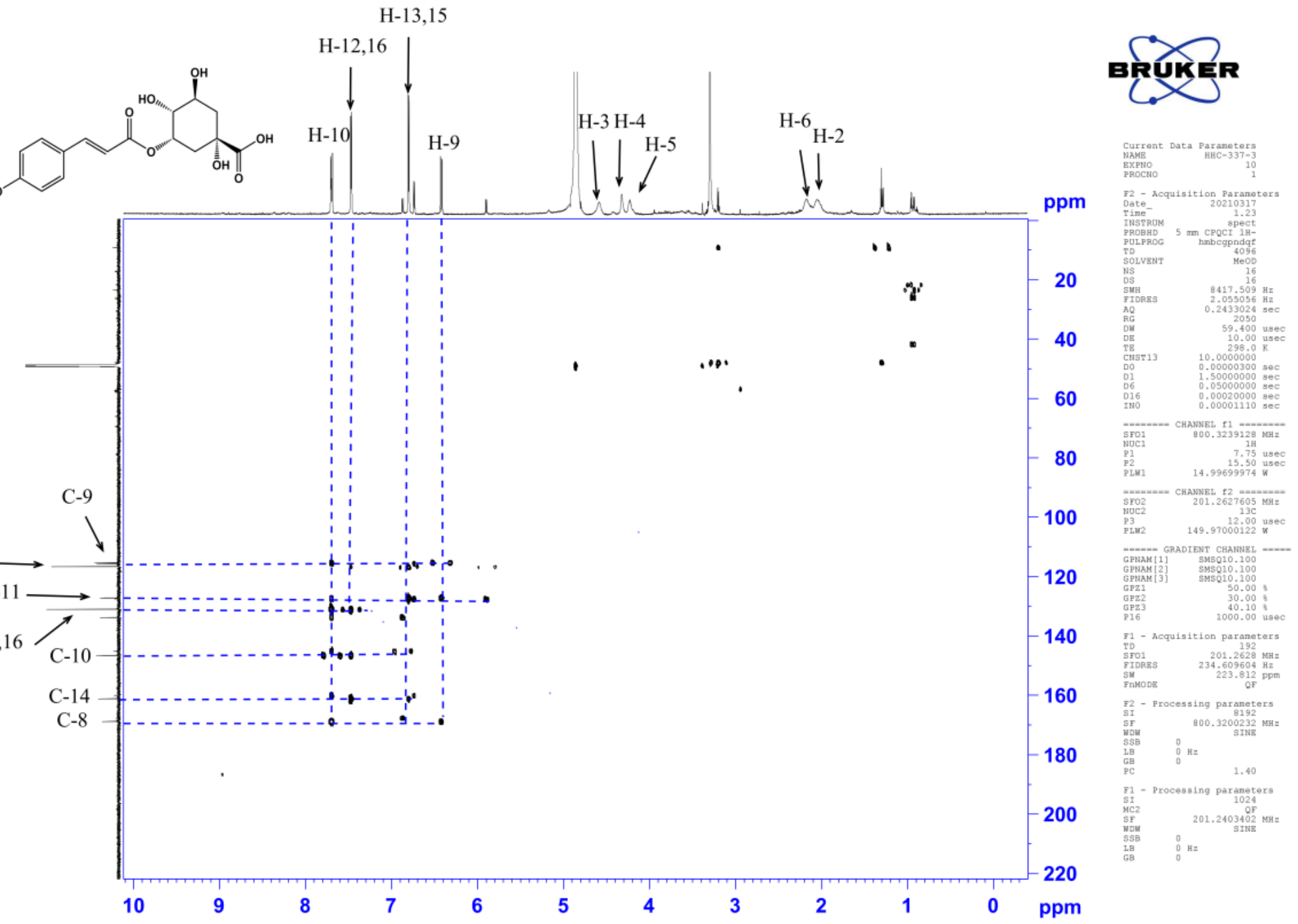

H-13,15
H-12,16
H-10
H-9
H-3
H-4
H-5
H-6
H-2
BRUKER
C-9
C-13,15
C-11
C-12,16
C-10
C-14
C-8
ppm

F

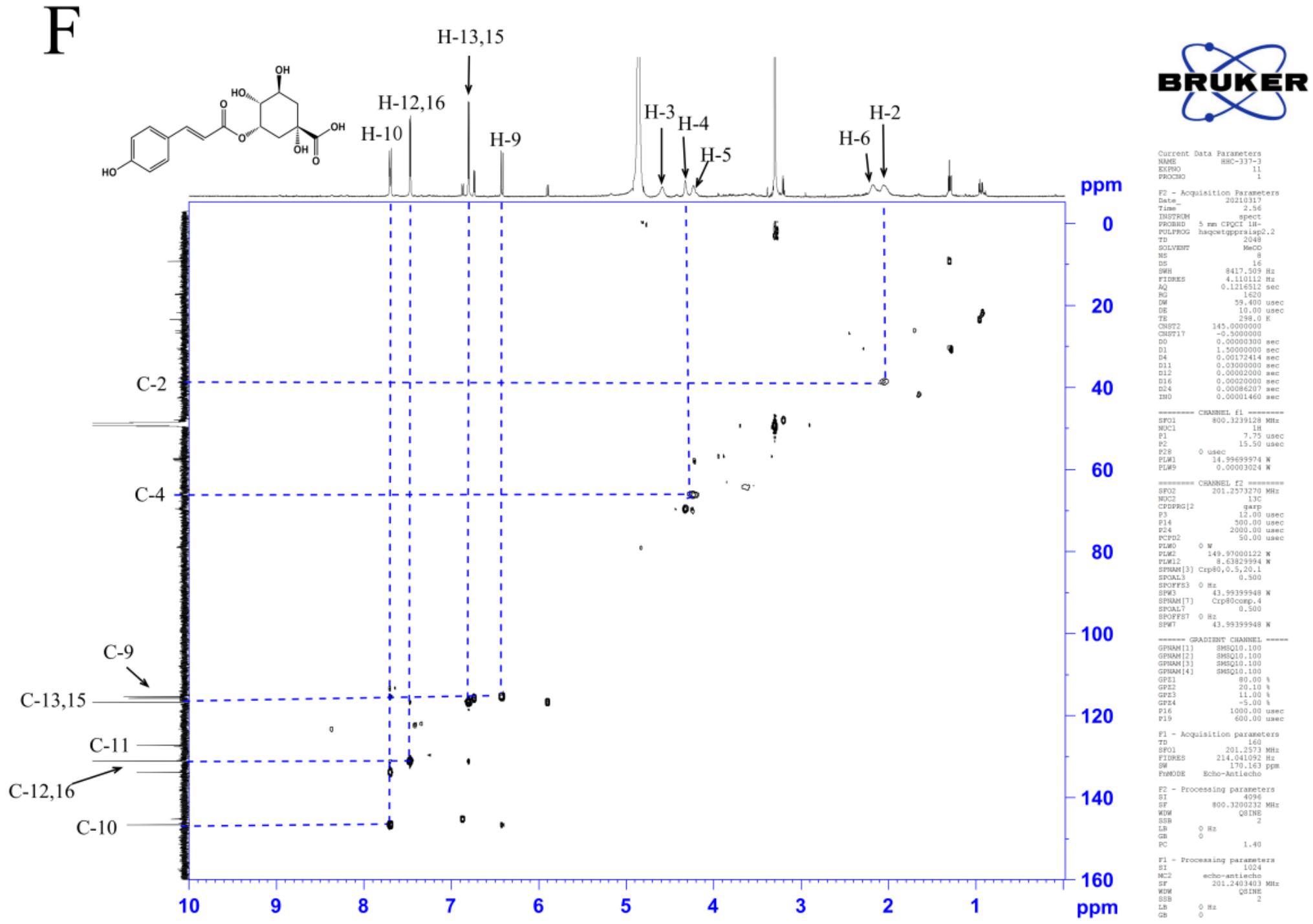

H-13,15
H-12,16
H-10
H-9
H-3
H-4
H-5
H-6
H-2
BRUKER
C-2
C-4
C-9
C-13,15
C-11
C-12,16
C-10
ppm

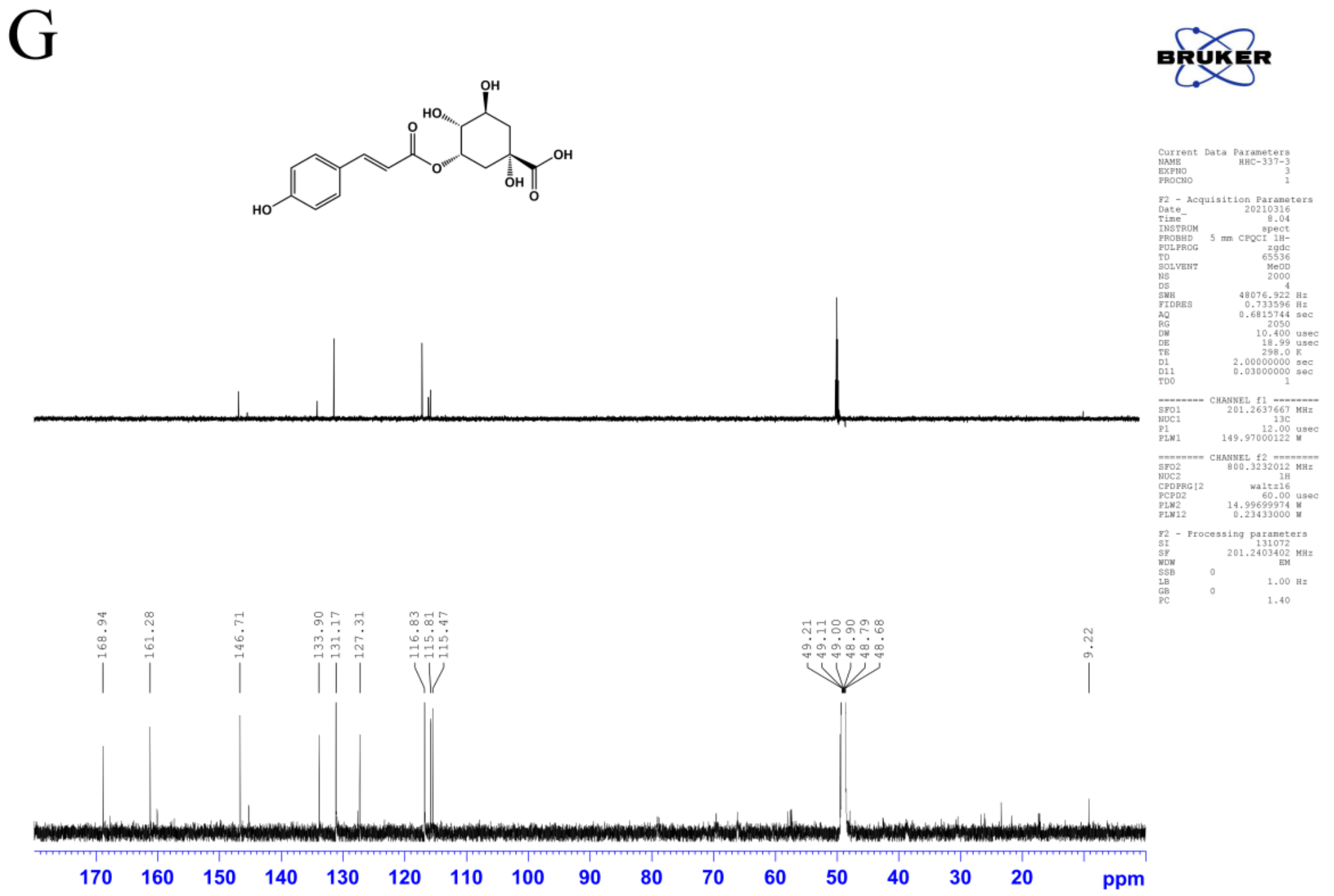

Fig. S7 The MS/MS(A), ^{1}H-NMR(B), ^{13}C-NMR(C), ^{1}H-^{1}HCOSY(D), HMBC(E), HSQC(F) and DEPT135(G) data of compound **1**.

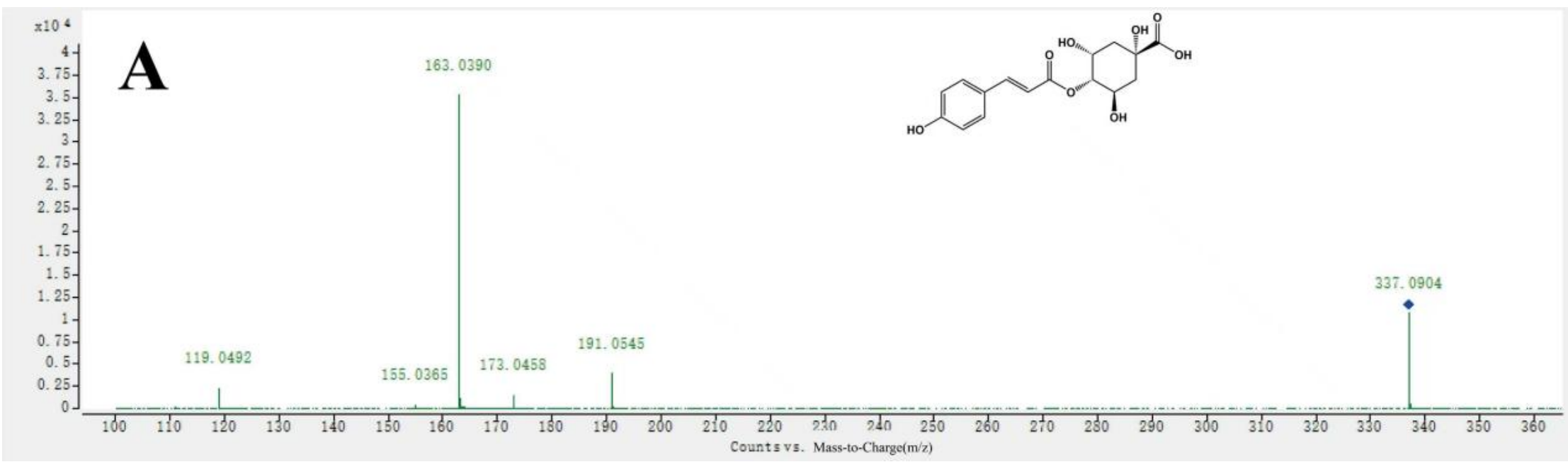

B

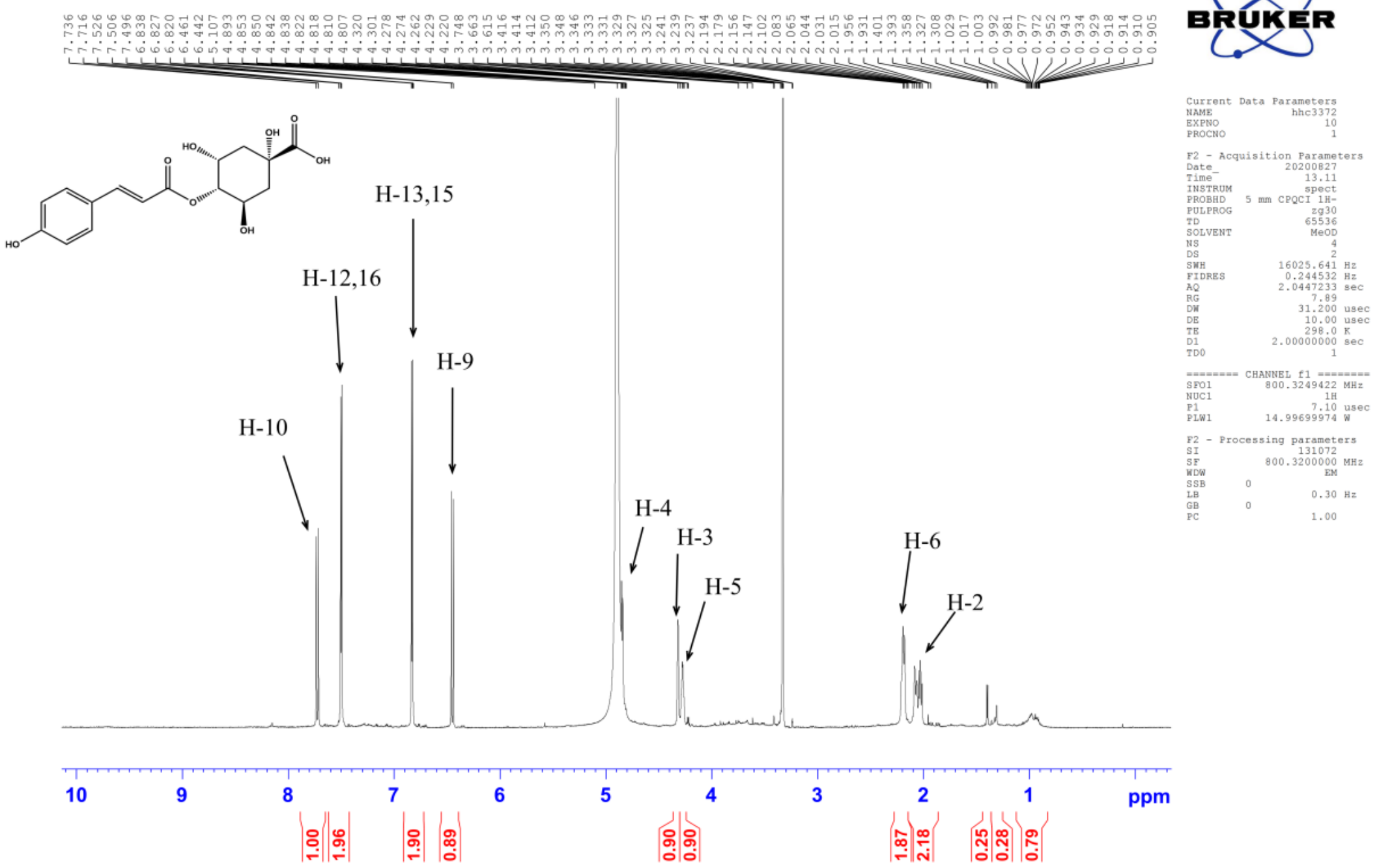
BRUKER
H-10
H-12,16
H-13,15
H-9
H-4
H-3
H-5
H-6
H-2
10
9
8
7
6
5
4
3
2
1
ppm
1.00
1.96
1.90
0.89
0.90
0.90
1.87
2.18
0.25
0.28
0.79

C

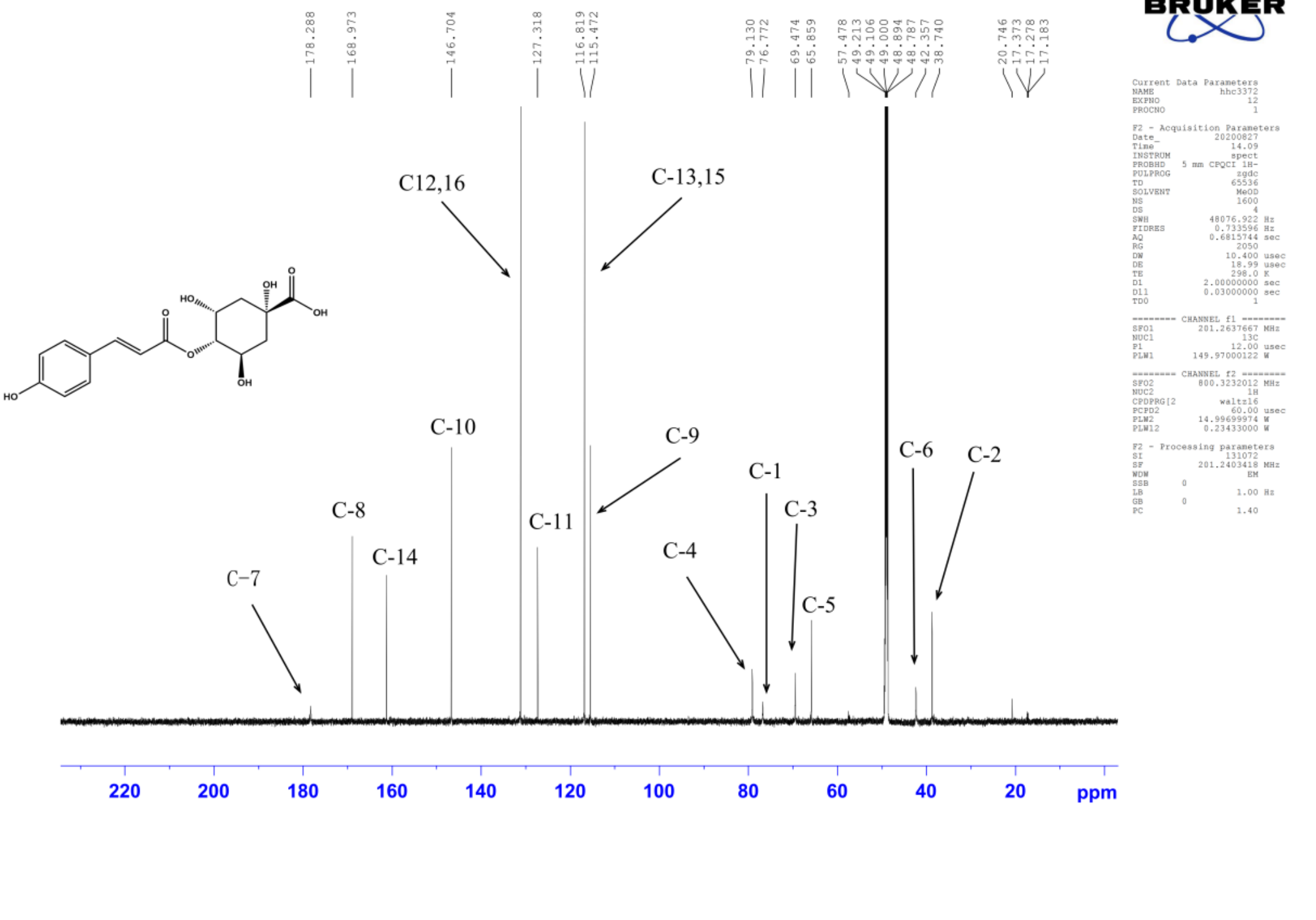
BRUKER
178.288
168.973
146.704
127.318
116.819
115.472
79.130
76.772
69.474
65.859
57.478
49.213
49.106
49.000
48.894
48.787
42.357
38.740
20.746
17.373
17.278
17.183
C12,16
C-13,15
C-10
C-9
C-8
C-11
C-1
C-3
C-14
C-4
C-7
C-5
C-6
C-2
220
200
180
160
140
120
100
80
60
40
20
ppm

D

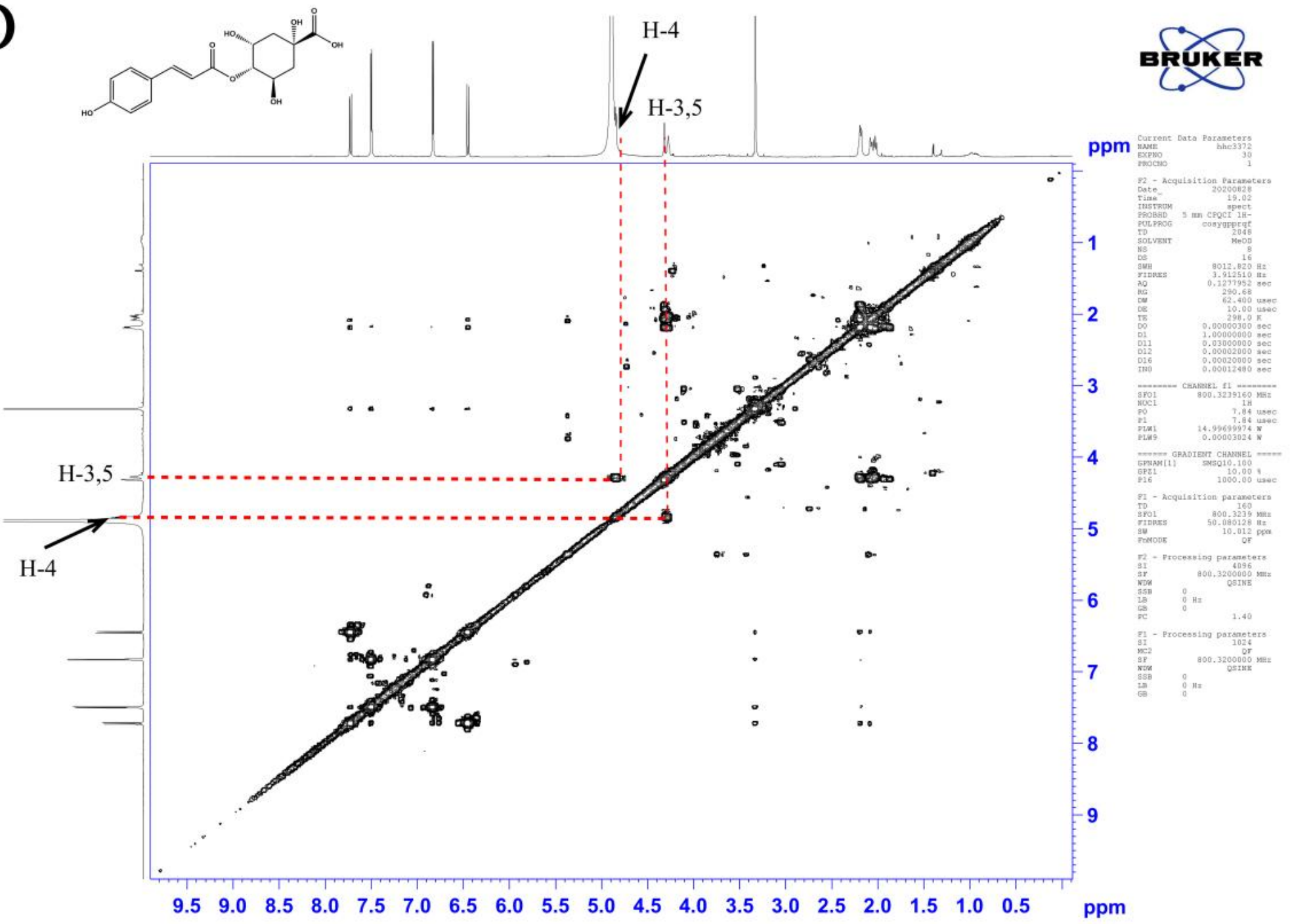

H-4
H-3,5
BRUKER
ppm
H-3,5
H-4
1
2
3
4
5
6
7
8
9
9.5 9.0 8.5 8.0 7.5 7.0 6.5 6.0 5.5 5.0 4.5 4.0 3.5 3.0 2.5 2.0 1.5 1.0 0.5
ppm

E

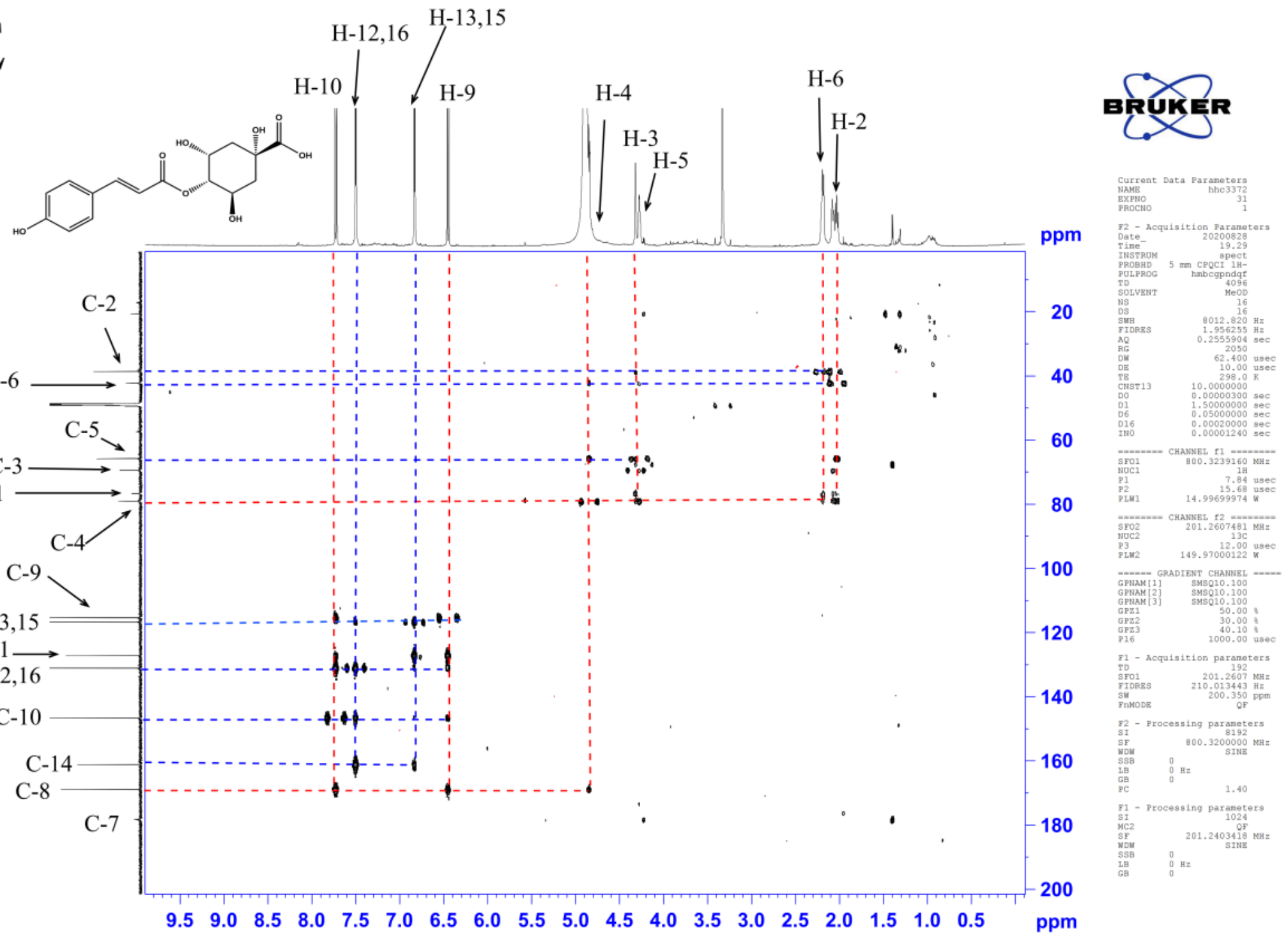

H-12,16
H-13,15
H-10
H-9
H-4
H-6
H-2
H-3
H-5
BRUKER
ppm
C-2
C-6
C-5
C-3
C-1
C-4
C-9
C-13,15
C-11
C-12,16
C-10
C-14
C-8
C-7
20
40
60
80
100
120
140
160
180
200
9.5 9.0 8.5 8.0 7.5 7.0 6.5 6.0 5.5 5.0 4.5 4.0 3.5 3.0 2.5 2.0 1.5 1.0 0.5
ppm

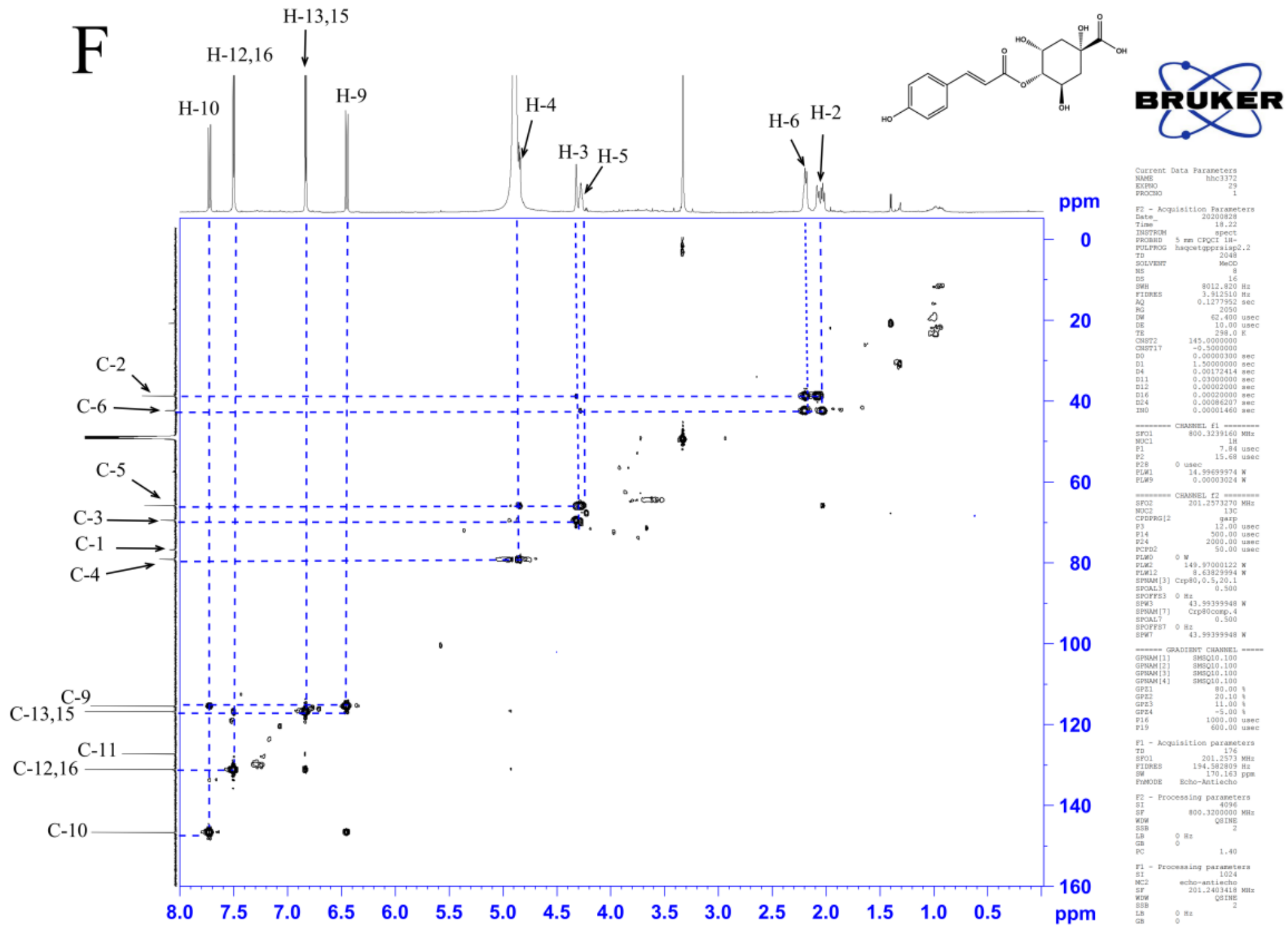

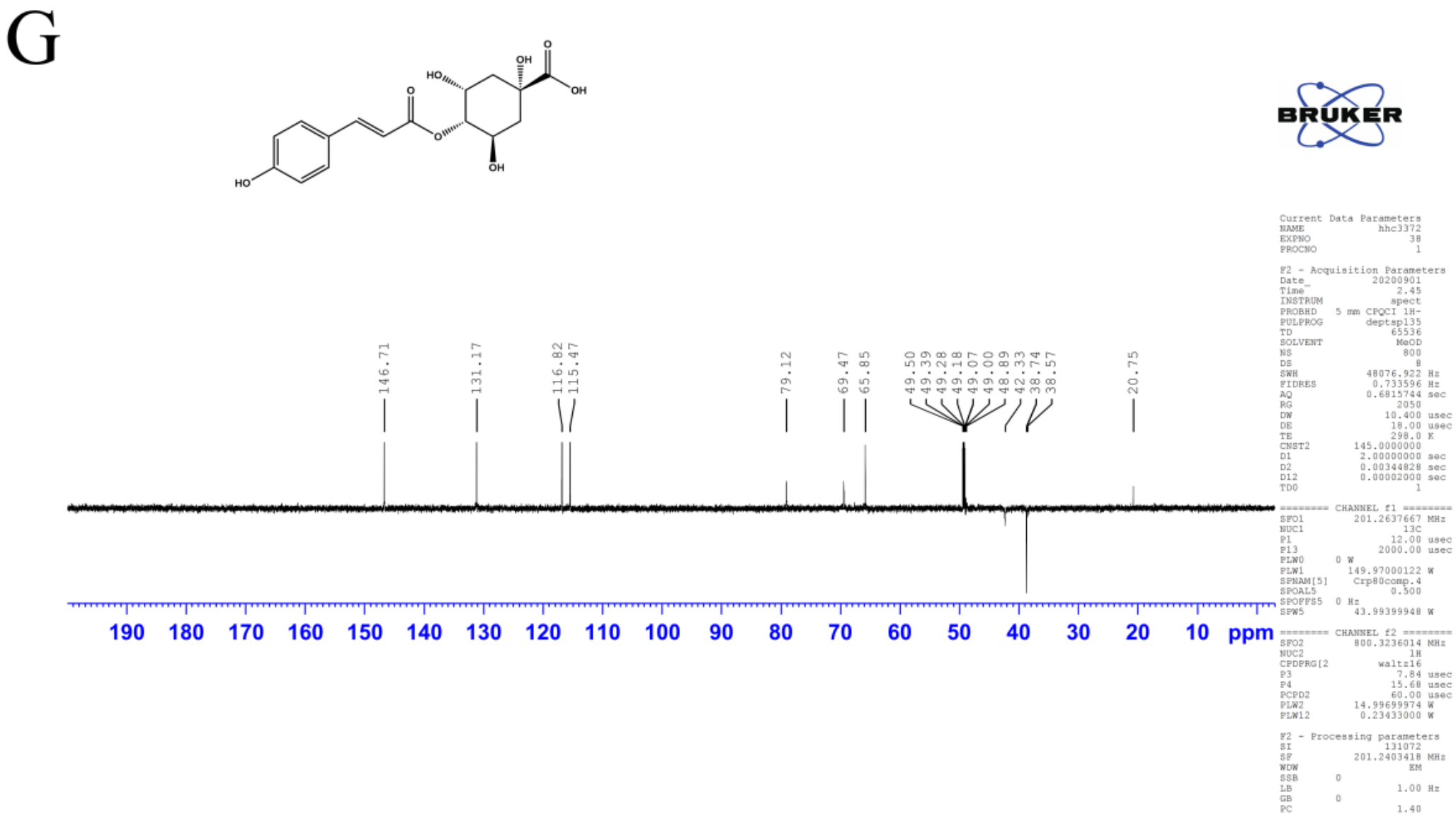

Fig. S8 The MS/MS(A), ^{1}H-NMR(B), ^{13}C-NMR(C), ^{1}H-^{1}HCOSY(D), HMBC(E), HSQC(F) and DEPT135(G) data of compound **2**.

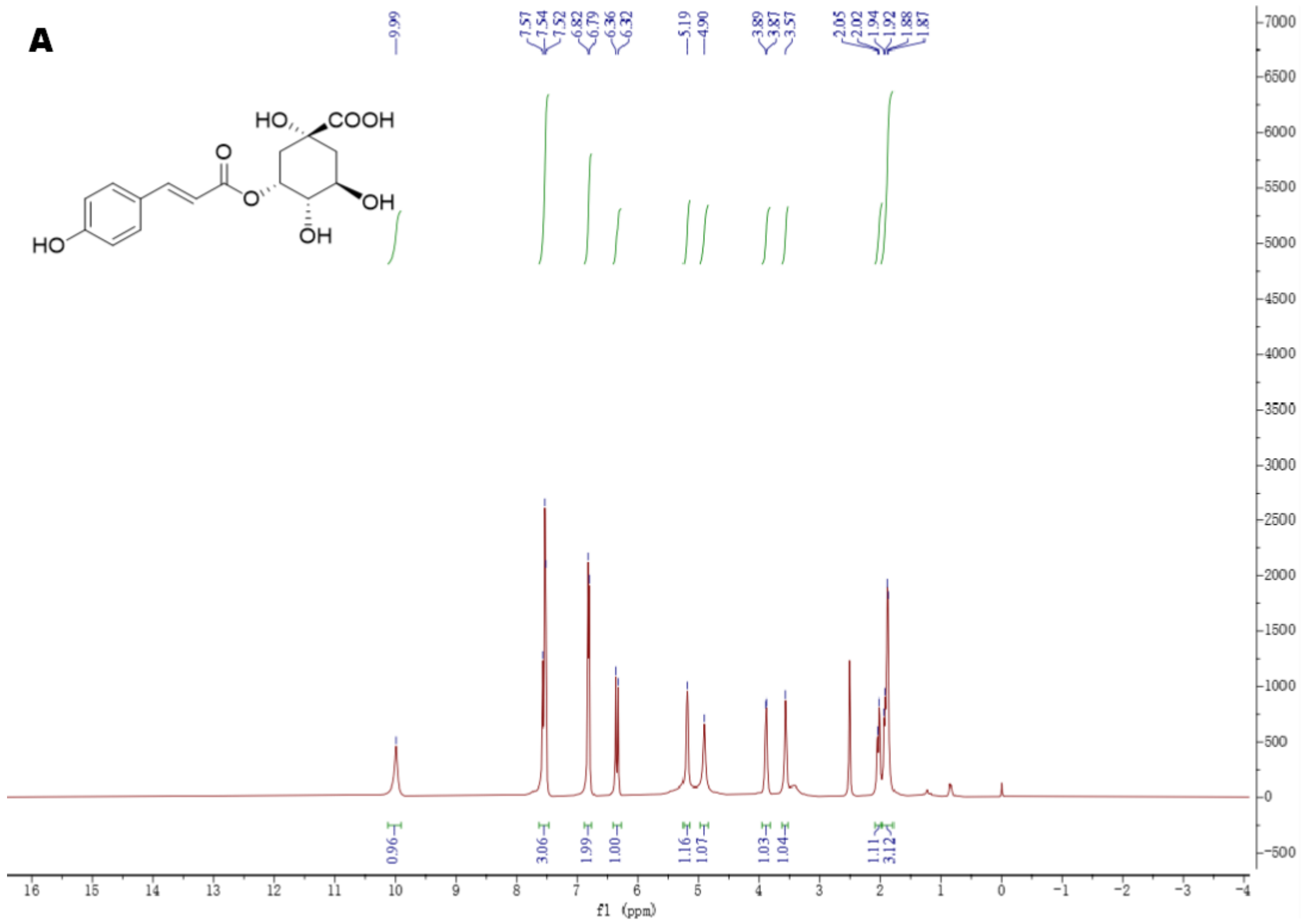
A
HO
COOH
O
OH
HO
OH
9.99
7.57
7.54
7.52
6.82
6.79
6.36
6.32
5.19
4.90
3.89
3.87
3.57
2.05
2.02
1.94
1.92
1.88
1.87
0.96
3.06
1.99
1.00
1.16
1.07
1.03
1.04
1.11
3.12
f1 (ppm)

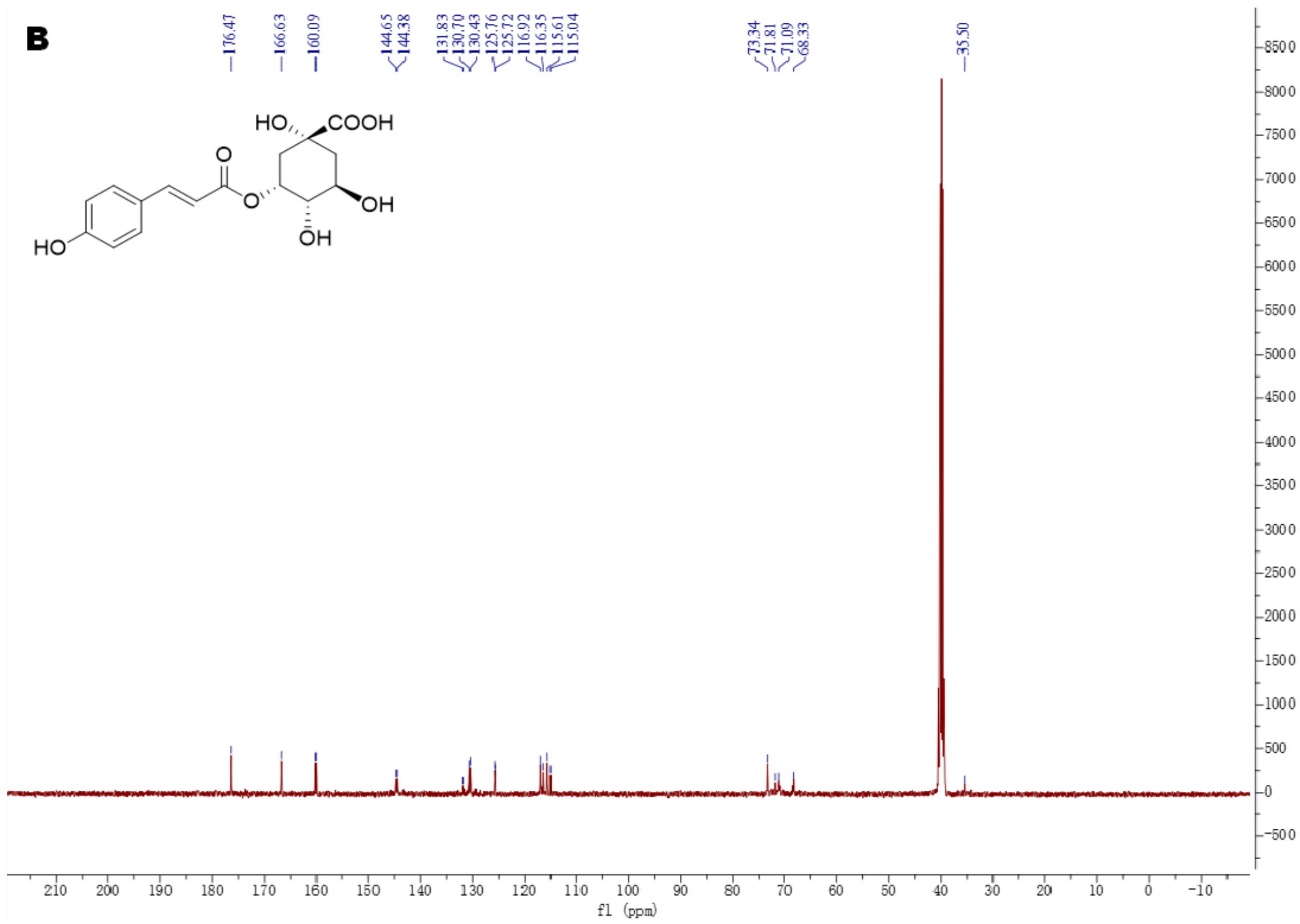
B
HO
COOH
O
OH
HO
OH
176.47
166.63
160.09
144.65
144.38
131.83
130.70
130.43
125.76
125.72
116.92
116.35
115.61
115.04
73.34
71.81
71.09
68.33
35.50
f1 (ppm)

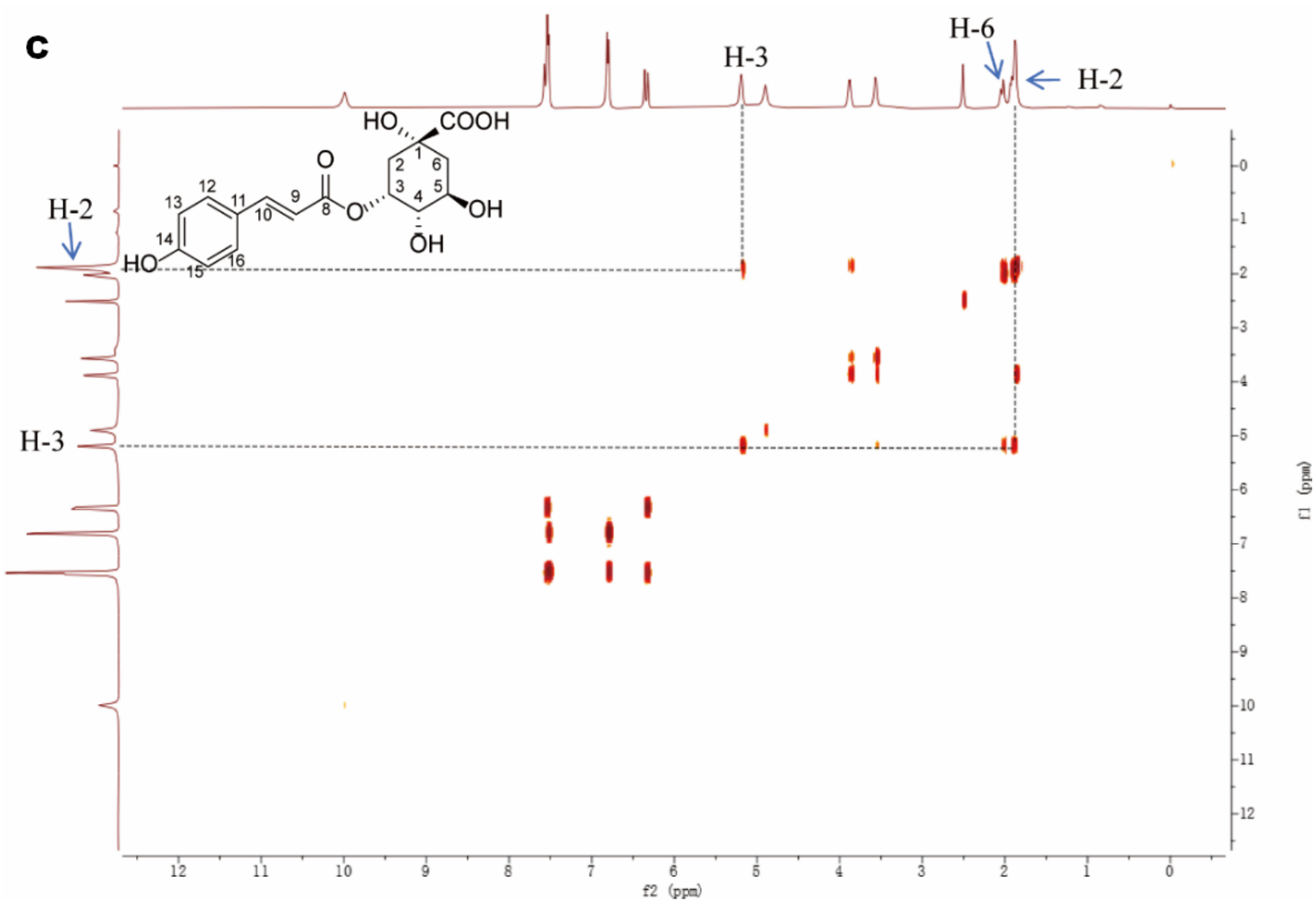
C
H-3
H-6
H-2
HO
COOH
OH
H-2
H-3
f2 (ppm)
f1 (ppm)

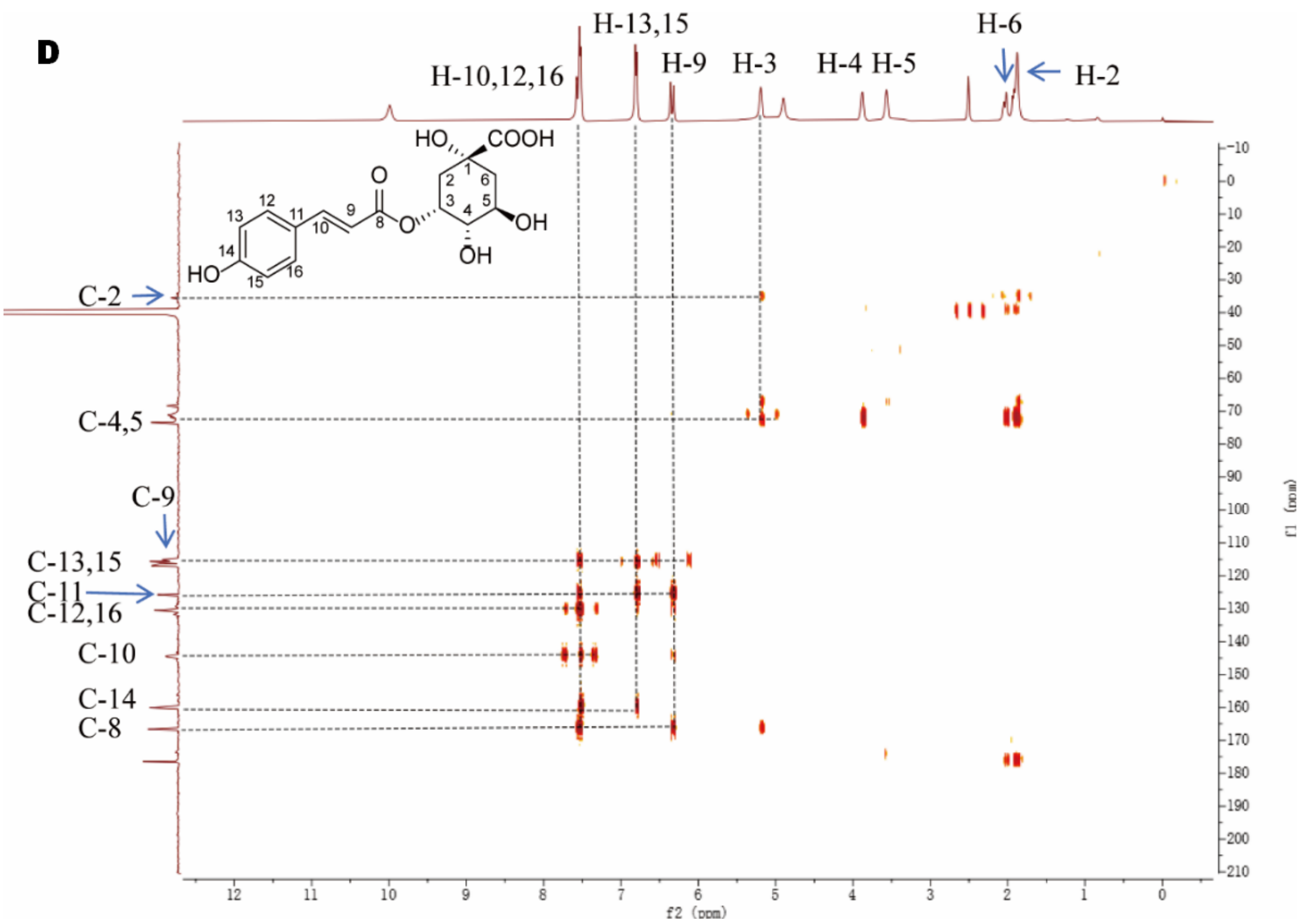
D
H-13,15
H-10,12,16
H-9
H-3
H-4
H-5
H-6
H-2
HO
COOH
OH
C-2
C-4,5
C-9
C-13,15
C-11
C-12,16
C-10
C-14
C-8
f2 (ppm)
f1 (ppm)

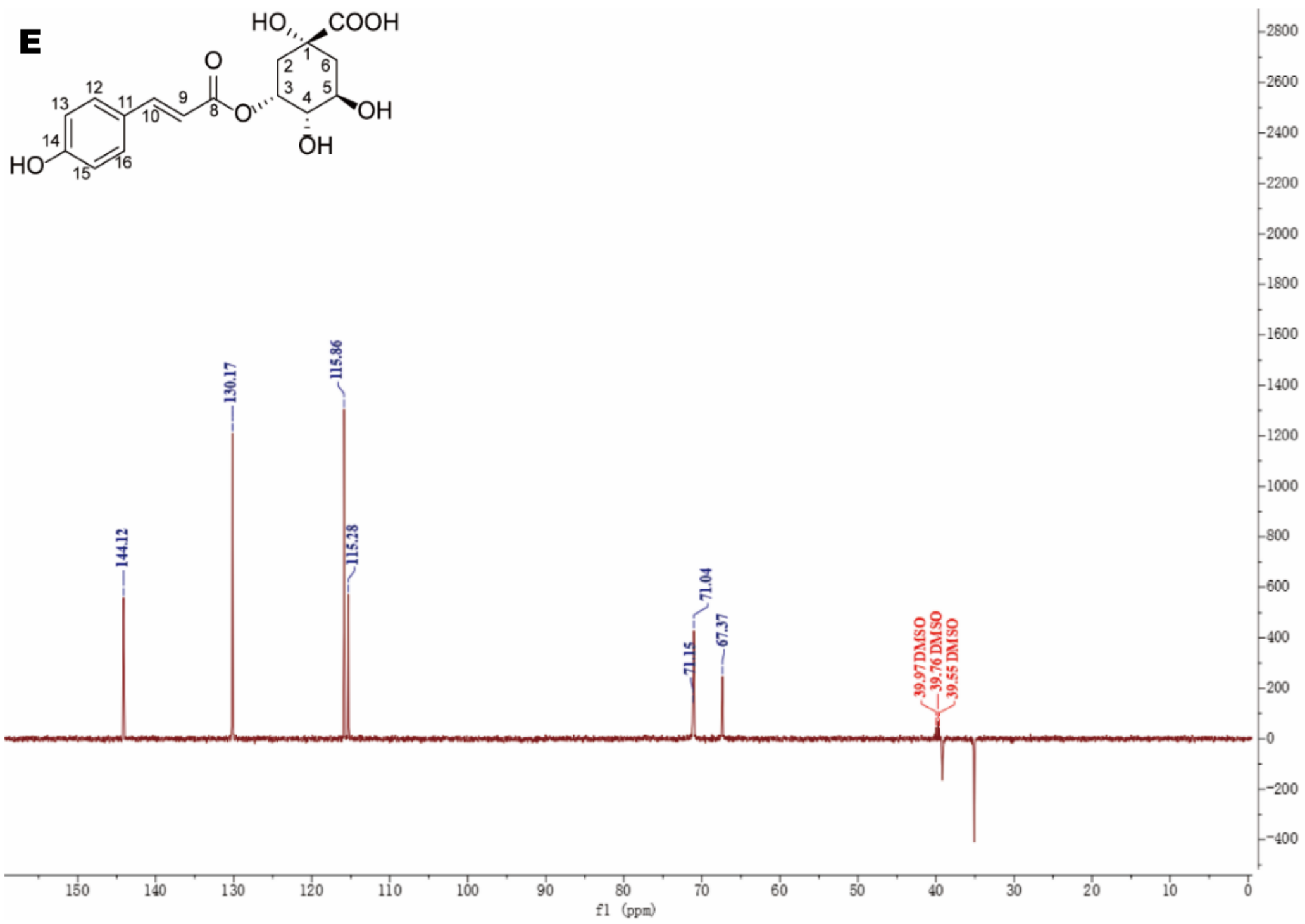

Fig. S9 The ^{1}H-NMR(A), ^{13}C-NMR(B), ^{1}H-^{1}HCOSY(C)，HMBC(D) and DEPT135(E) data of the synthesized compound **1**.

White solid, HRMS (ESI$^-$)：calcd for $C_{16}H_{17}O_8^-$ [M-H]$^-$, theoretical *m/z* 337.0923; found *m/z* 337.0925. ^{1}H-NMR (400 MHz, DMSO-d6): δ 9.99 (s, 1H), 7.57-7.52 (m, 3H), 6.81 (d, *J*= 12 Hz, 2H), 6.34 (d, *J*= 16 Hz, 1H), 5.19 (s, 1H), 4.90 (s, 1H), 3.88 (d, *J*= 8 Hz, 1H), 3.57 (s, 1H), 2.04 (d, *J*= 12 Hz, 1H), 1.94-1.87(m, 3H). ^{13}C-NMR (100 MHz, DMSO-d6): δ 175.36, 166.25, 160.24, 144.98, 130.71(2×CH), 125.64, 116.23(2×CH), 115.00, 74.01, 71.33, 70.97, 68.67, 37.66, 36.85.

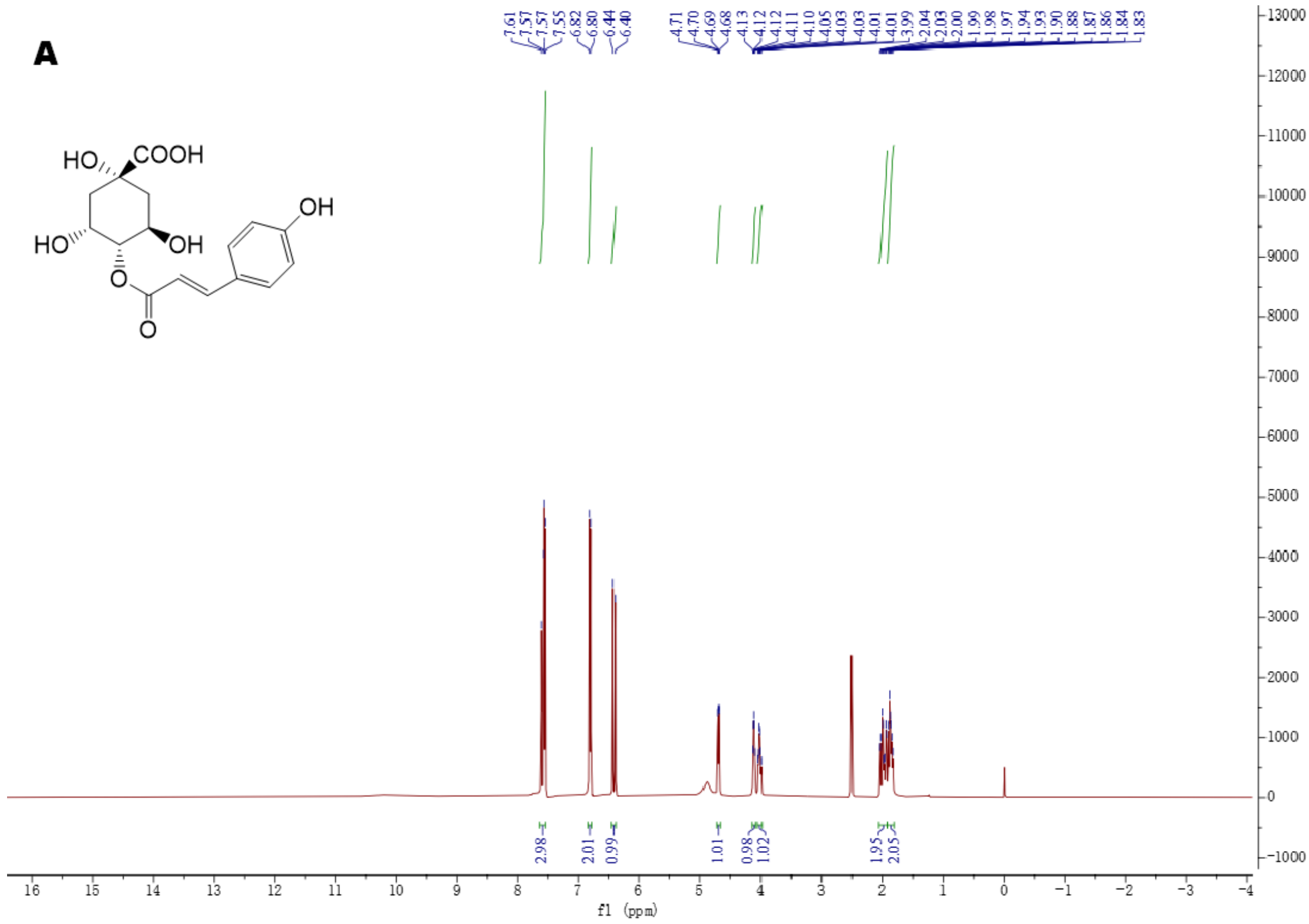
A
HO
COOH
OH
HO
OH
O
O
7.61
7.57
7.57
7.55
6.82
6.80
6.44
6.40
4.71
4.70
4.69
4.68
4.13
4.12
4.12
4.11
4.10
4.05
4.03
4.03
4.01
4.01
3.99
2.04
2.03
2.00
1.99
1.98
1.97
1.94
1.93
1.90
1.88
1.87
1.86
1.84
1.83
2.98
2.01
0.99
1.01
0.98
1.02
1.95
2.05
f1 (ppm)

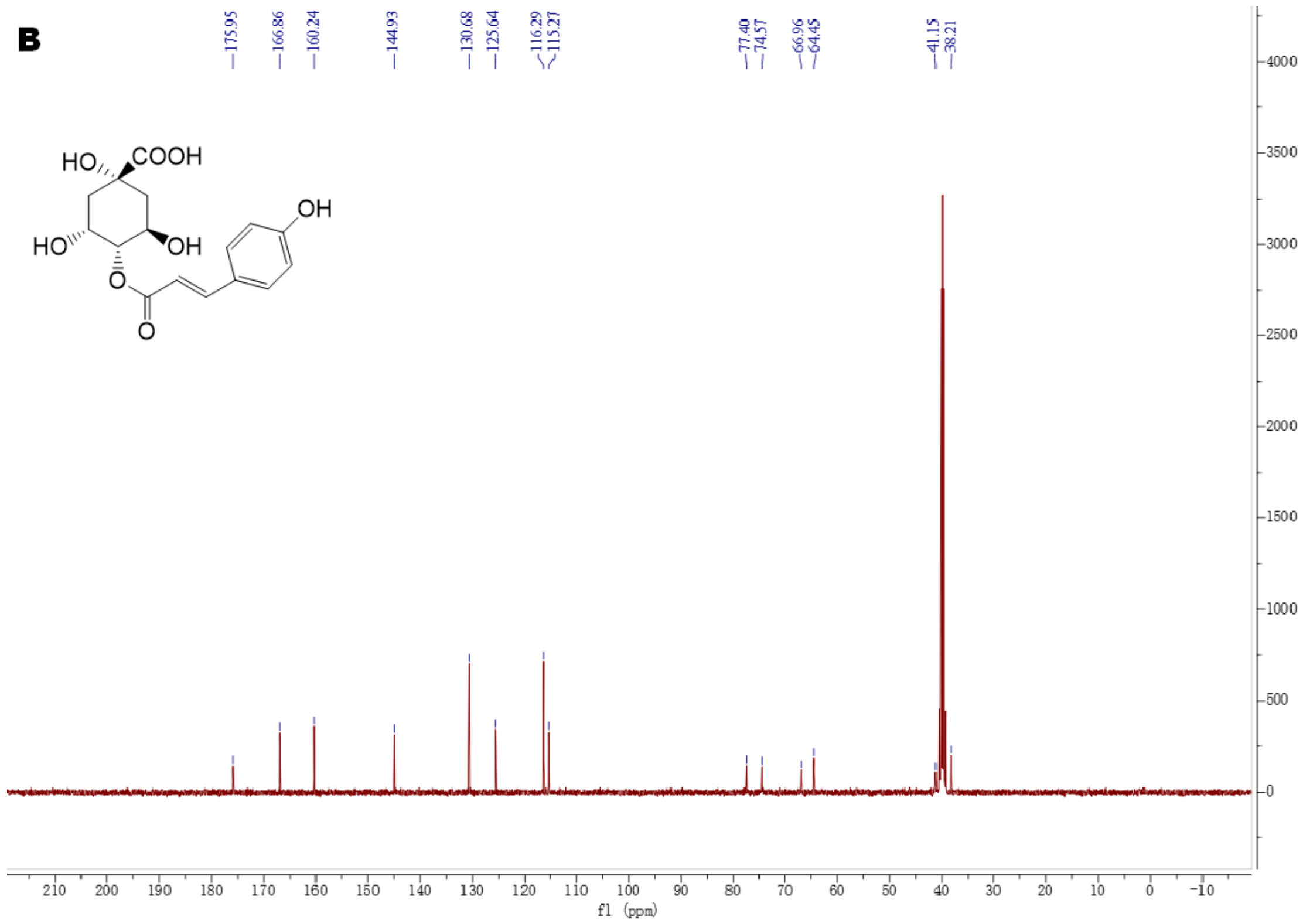
B
HO
COOH
OH
HO
OH
O
O
175.95
166.86
160.24
144.93
130.68
125.64
116.29
115.27
77.40
74.57
66.96
64.45
41.15
38.21
f1 (ppm)

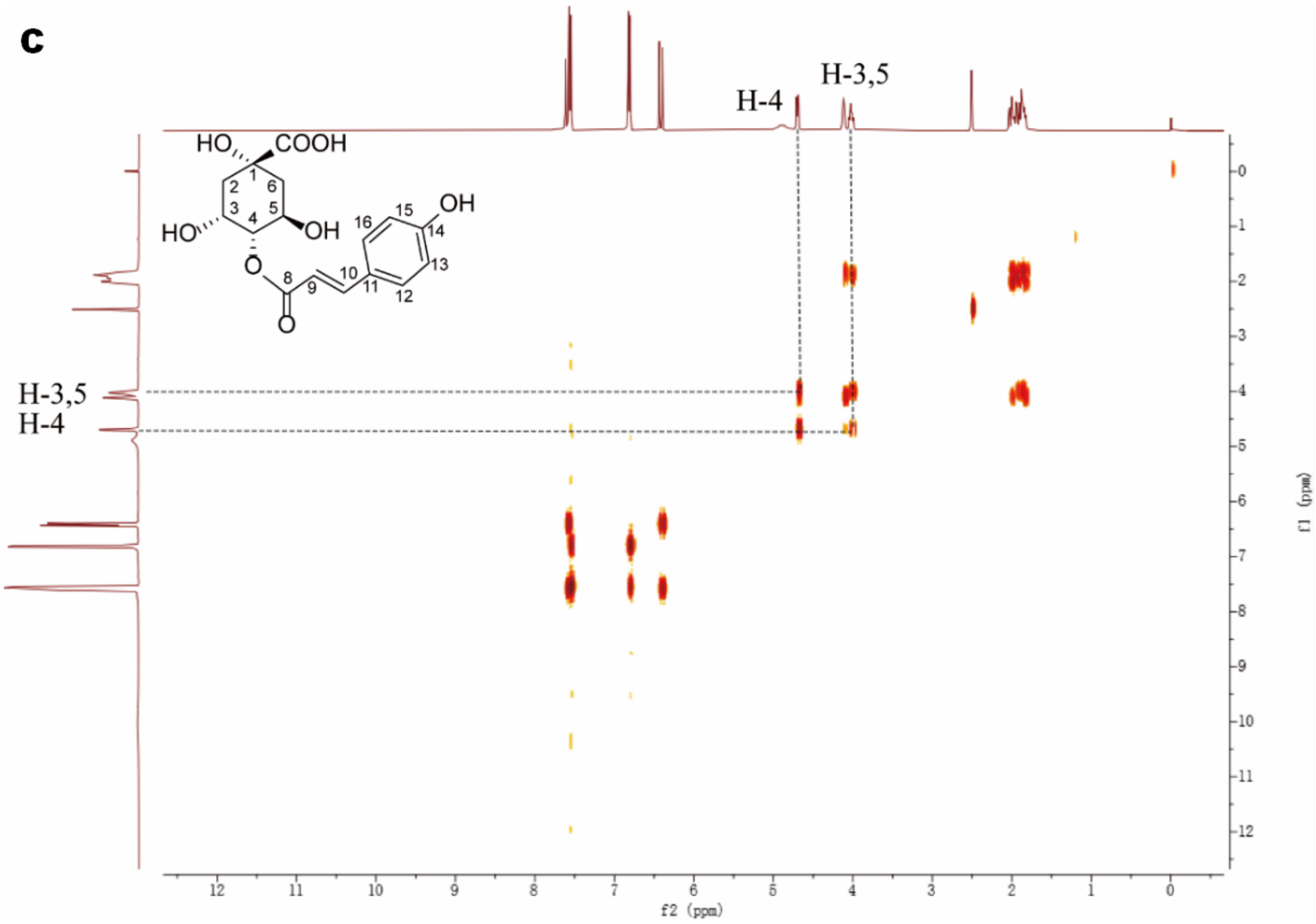
C
H-4
H-3,5
H-3,5
H-4
HO
COOH
HO
OH
OH
O
O
f2 (ppm)
f1 (ppm)

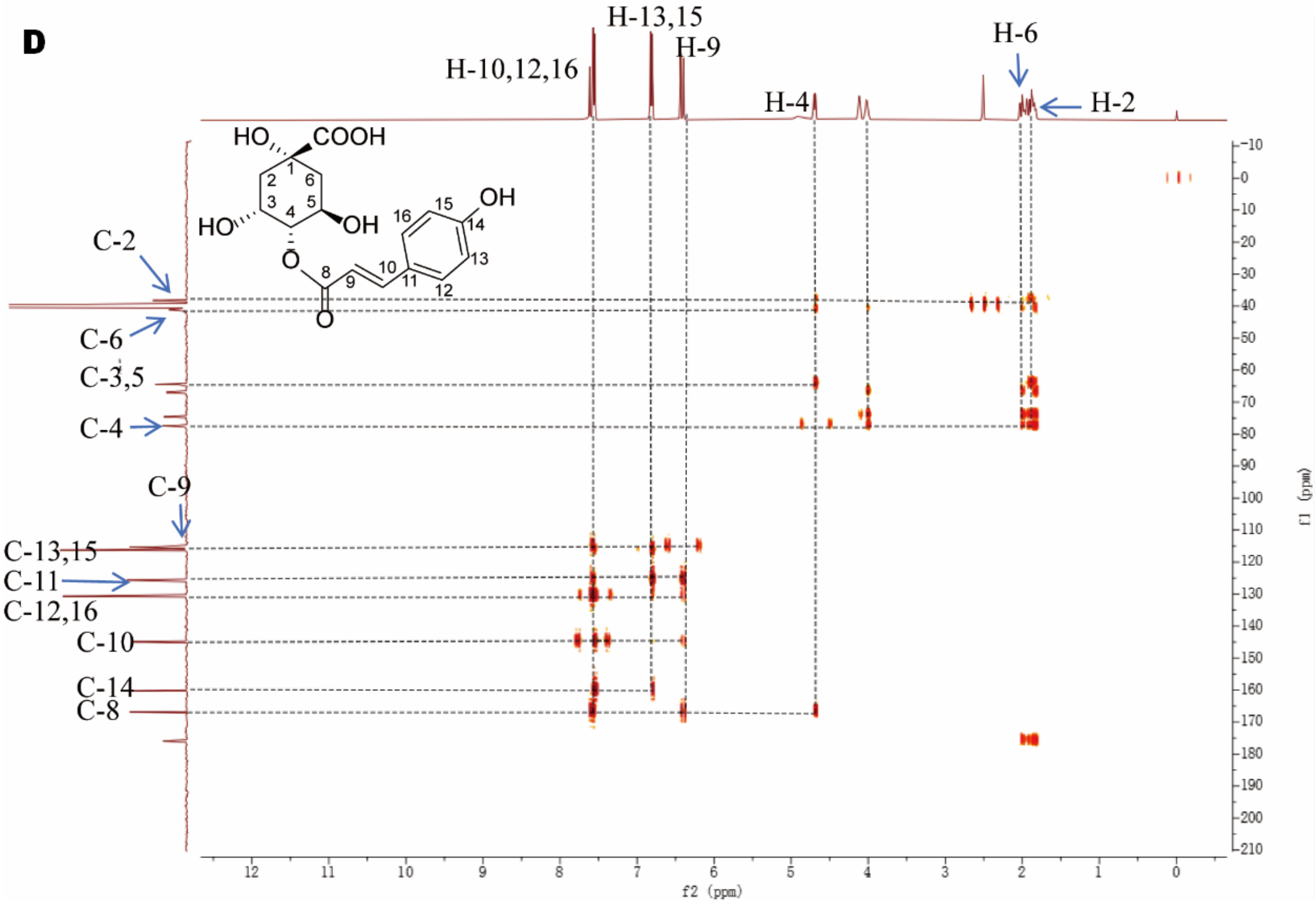
D
H-13,15
H-9
H-10,12,16
H-4
H-6
H-2
C-2
C-6
C-3,5
C-4
C-9
C-13,15
C-11
C-12,16
C-10
C-14
C-8
HO
COOH
HO
OH
OH
O
O
f2 (ppm)
f1 (ppm)

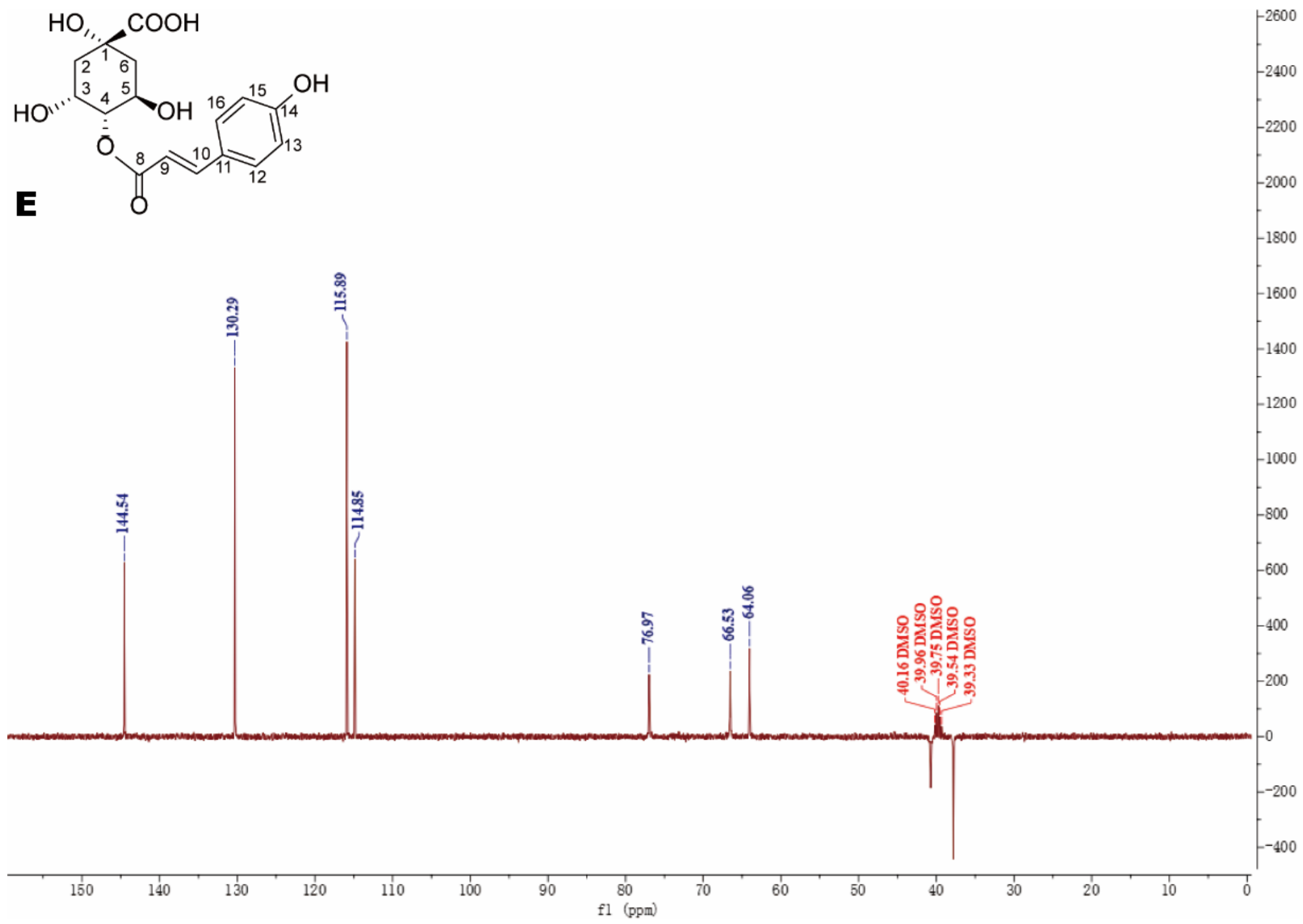

Fig. S10 The ^{1}H-NMR(A), ^{13}C-NMR(B), ^{1}H-^{1}HCOSY(C)，HMBC(D) and DEPT135(E) data of the synthesized compound **2**.

White solid, HRMS (ESI^{-})：calcd for $C_{16}H_{17}O_8^{-}$ [M-H]$^{-}$, theoretical *m/z* 337.0923; found *m/z* 337.0925.^{1}H NMR (400 MHz, DMSO-d6): δ 7.61-7.55 (m, 3H), 6.81 (d, *J*= 8 Hz, 2H), 6.42 (d, *J*= 16 Hz, 1H), 4.70 (dd, *J*= 4 Hz, *J*= 8 Hz, 1H), 4.13-4.10 (m, 1H), 4.05-3.99 (m, 1H), 2.04-1.93 (m, 2H), 1.90-1.83 (m, 2H). ^{13}C-NMR (100 MHz, DMSO-d6): δ 175.95, 166.86, 160.24, 144.93, 130.68(2×CH), 125.64, 116.29(2×CH), 115.27, 77.40, 74.57, 66.96, 64.45, 41.15, 38.21.

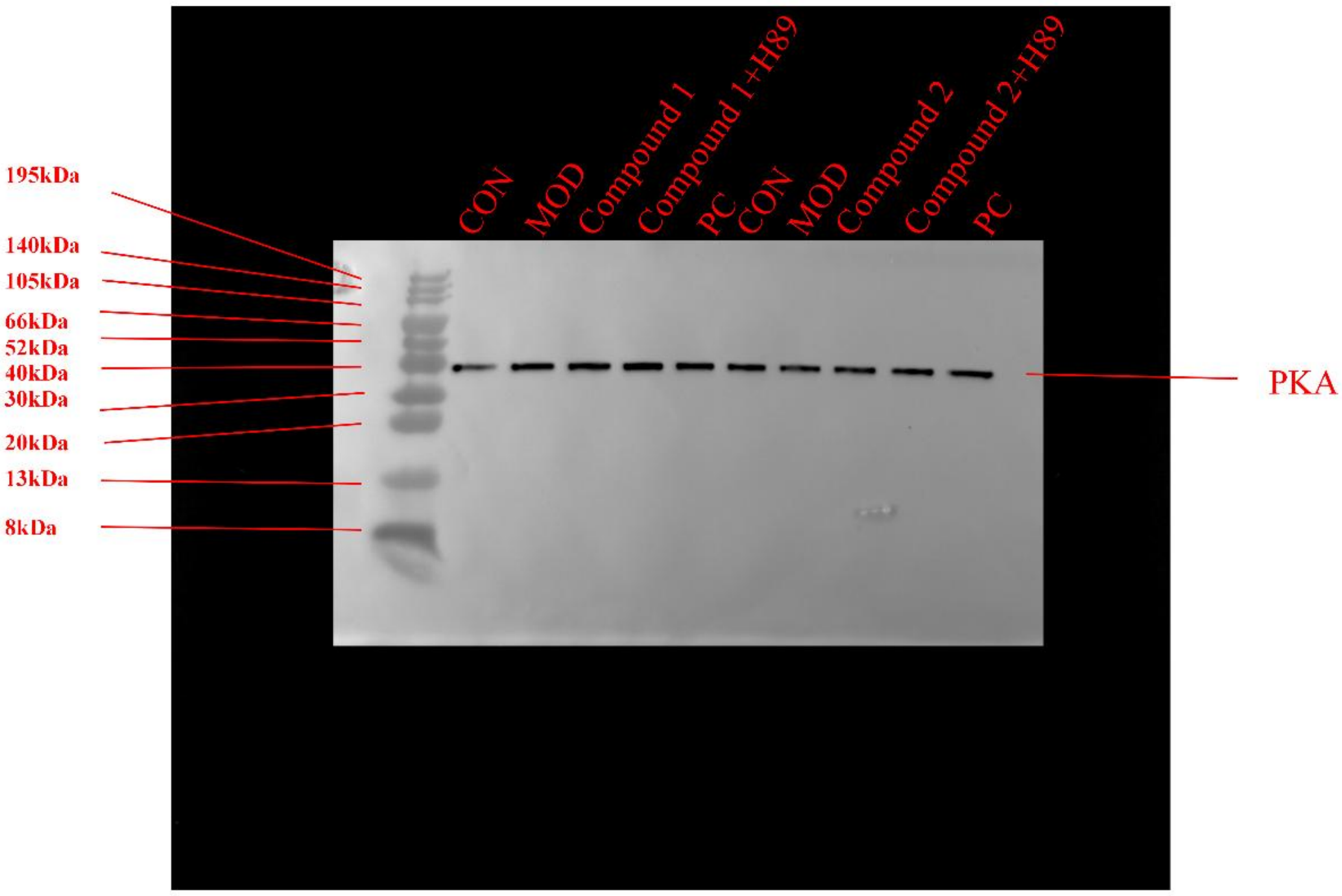

195kDa
140kDa
105kDa
66kDa
52kDa
40kDa
30kDa
20kDa
13kDa
8kDa
CON
MOD
Compound 1
Compound 1+H89
PC
CON
MOD
Compound 2
Compound 2+H89
PC
PKA

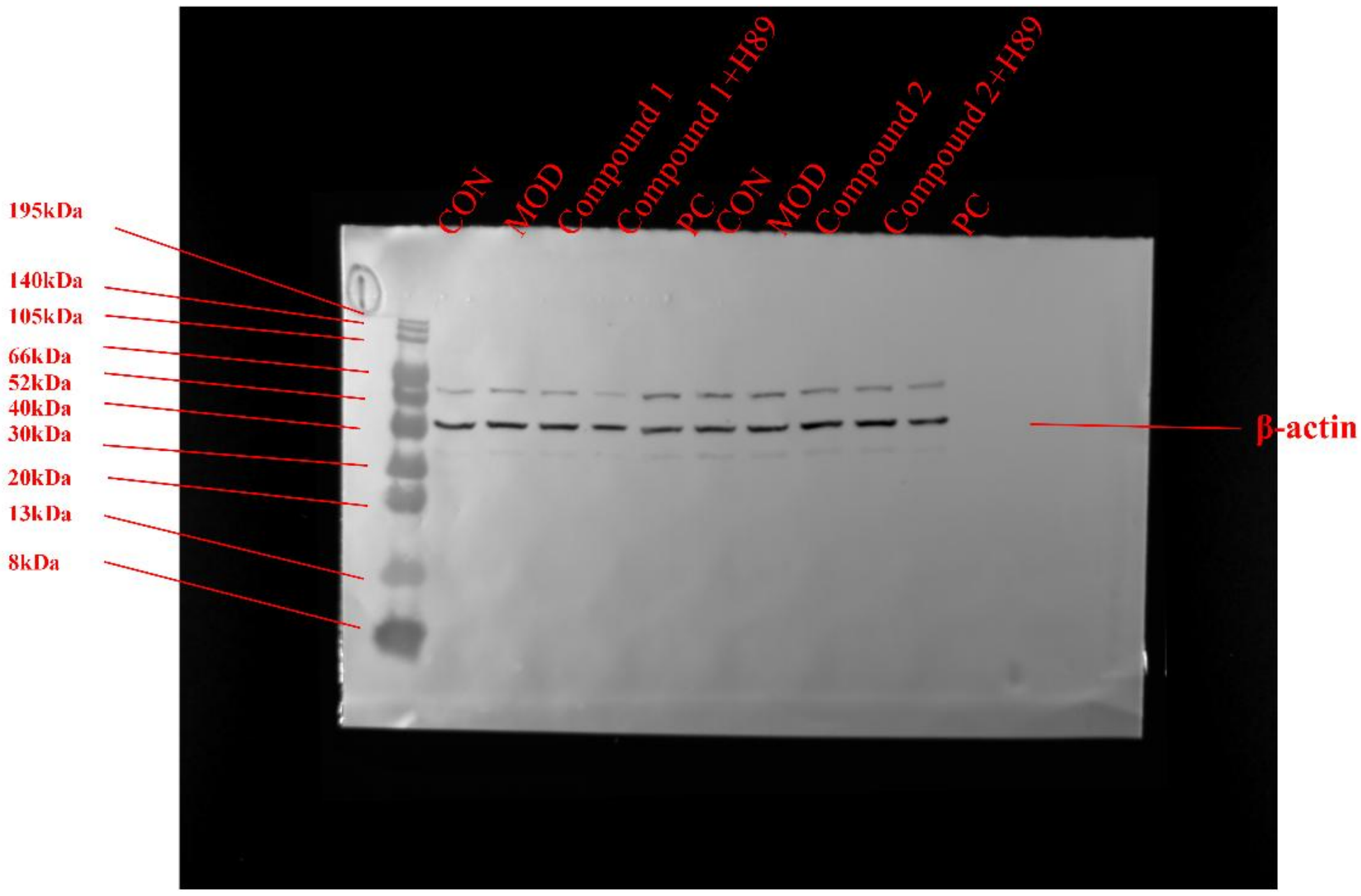

195kDa
140kDa
105kDa
66kDa
52kDa
40kDa
30kDa
20kDa
13kDa
8kDa
CON
MOD
Compound 1
Compound 1+H89
PC
CON
MOD
Compound 2
Compound 2+H89
PC
β-actin

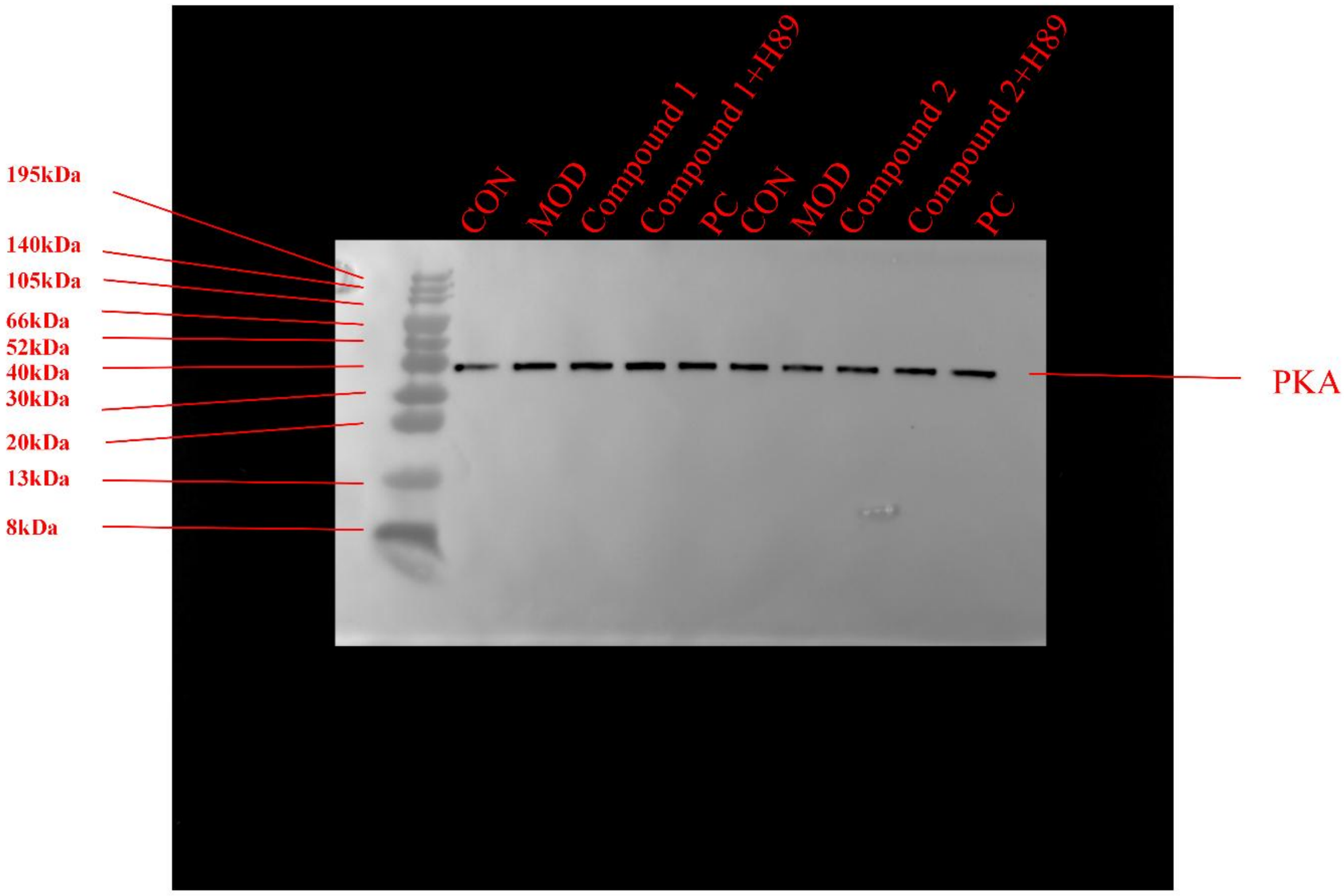

195kDa
140kDa
105kDa
66kDa
52kDa
40kDa
30kDa
20kDa
13kDa
8kDa
CON
MOD
Compound 1
Compound 1+H89
PC
CON
MOD
Compound 2
Compound 2+H89
PC
PKA

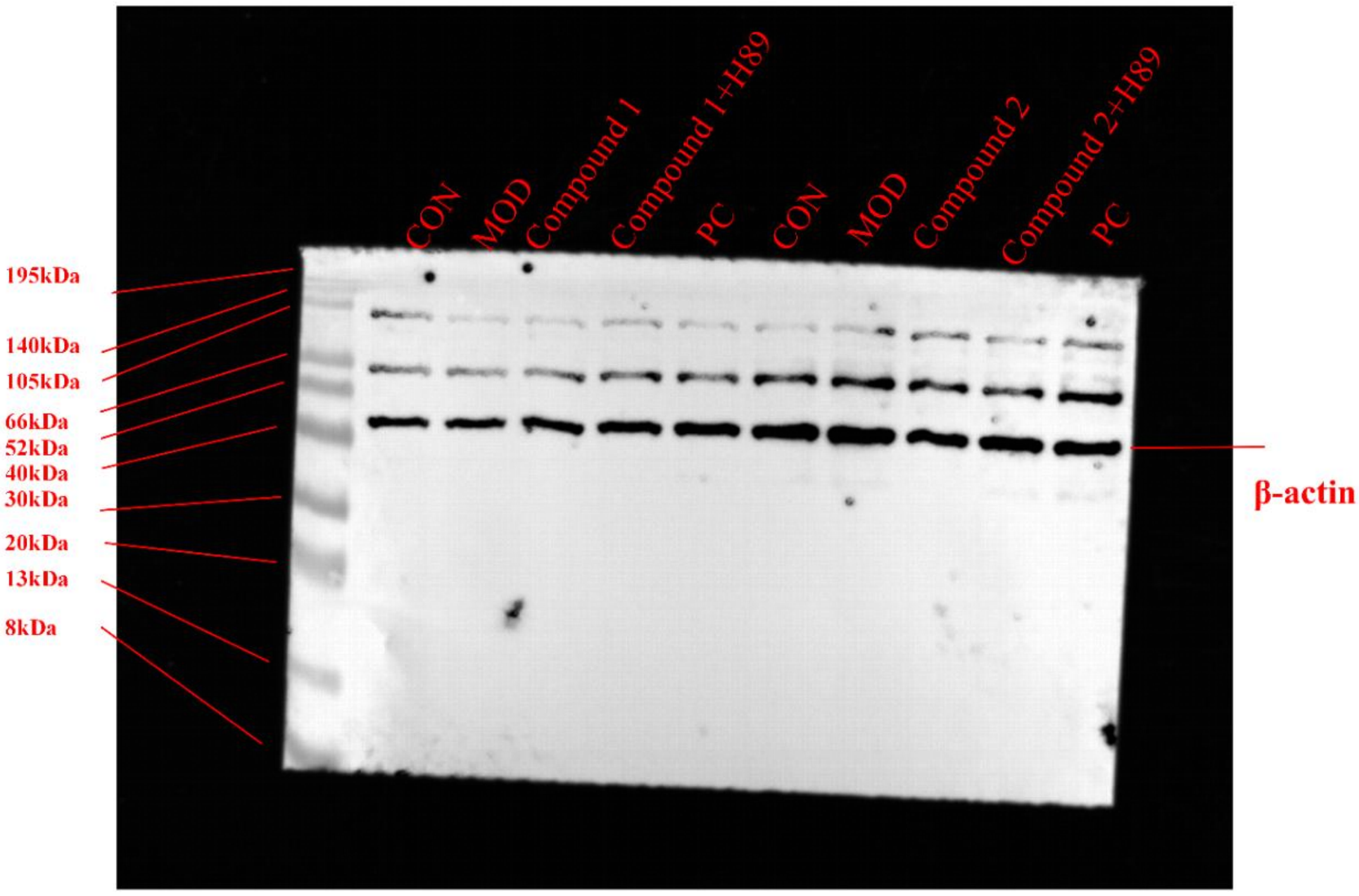

195kDa
140kDa
105kDa
66kDa
52kDa
40kDa
30kDa
20kDa
13kDa
8kDa
CON
MOD
Compound 1
Compound 1+H89
PC
CON
MOD
Compound 2
Compound 2+H89
PC
β-actin

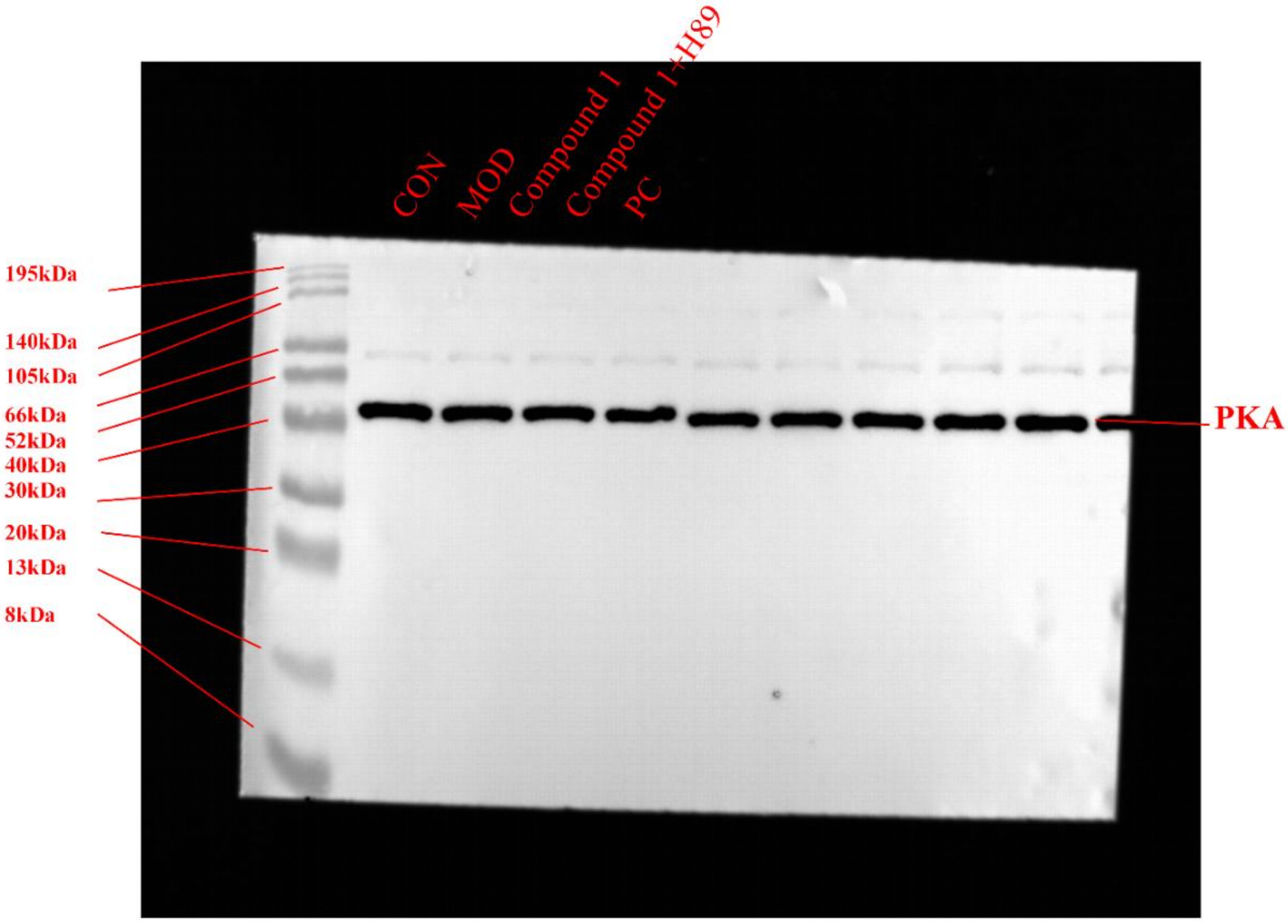
CON
MOD
Compound 1
Compound 1+H89
PC
195kDa
140kDa
105kDa
66kDa
52kDa
40kDa
30kDa
20kDa
13kDa
8kDa
PKA

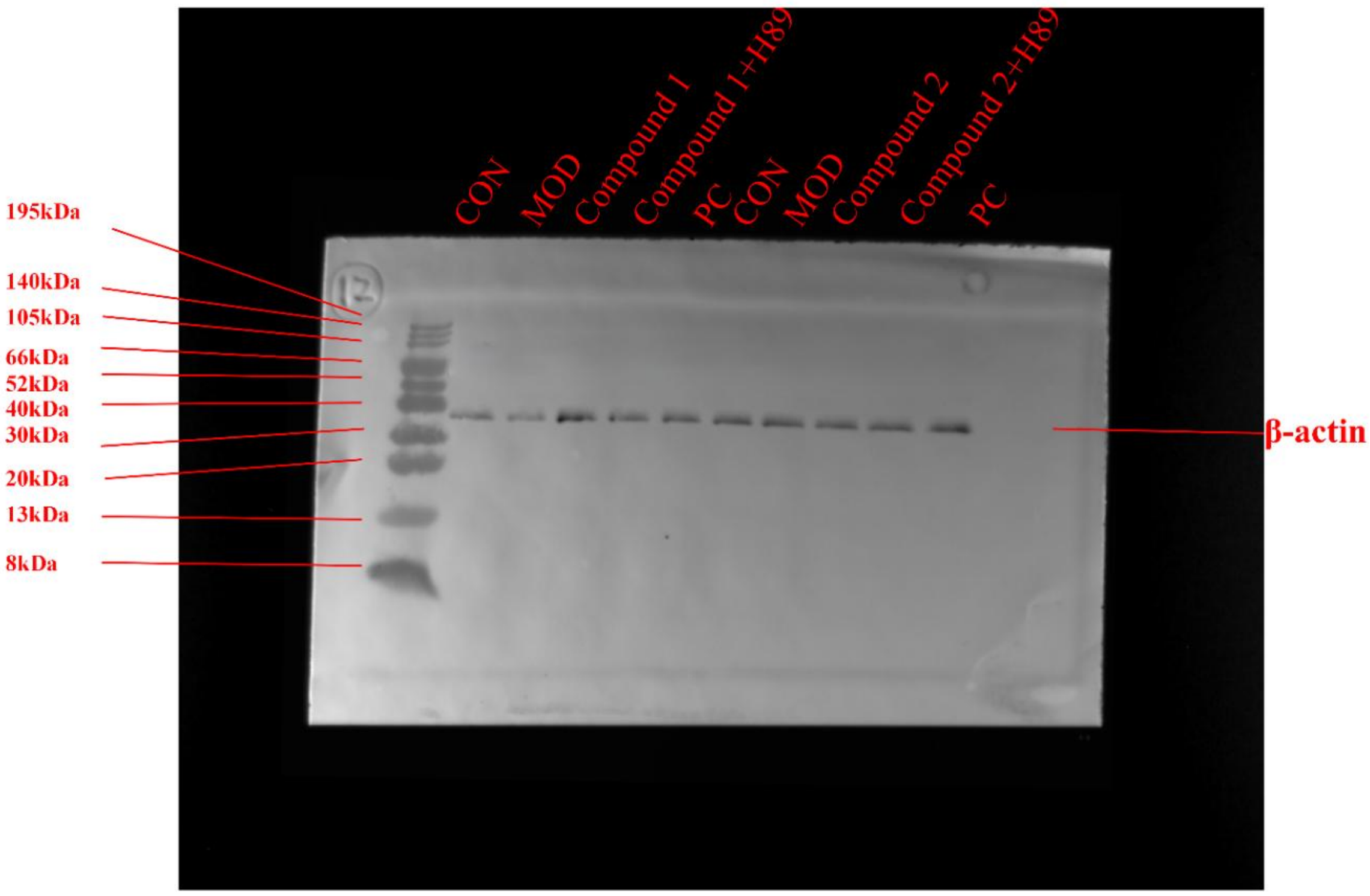
CON
MOD
Compound 1
Compound 1+H89
PC
CON
MOD
Compound 2
Compound 2+H89
PC
195kDa
140kDa
105kDa
66kDa
52kDa
40kDa
30kDa
20kDa
13kDa
8kDa
β-actin

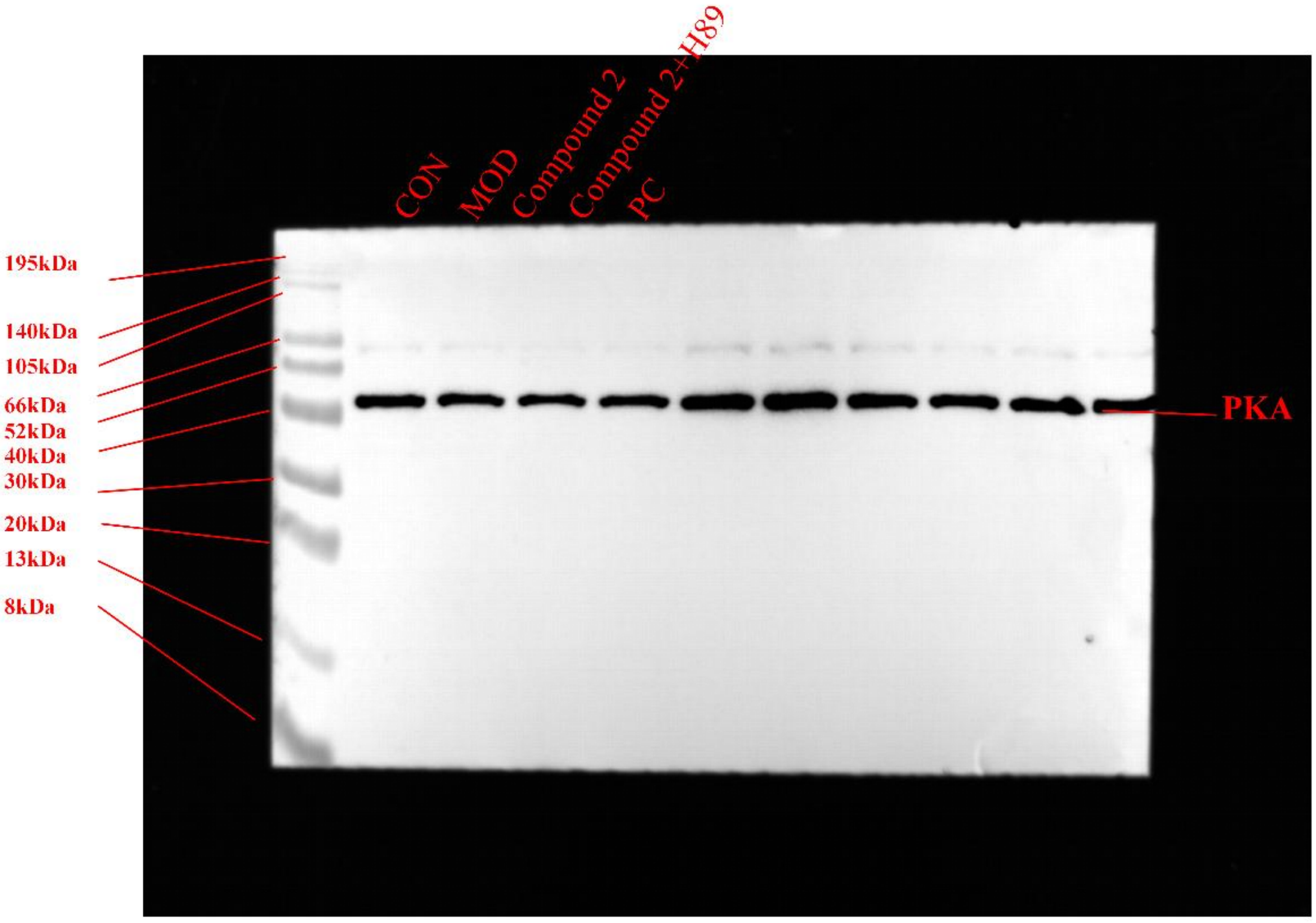

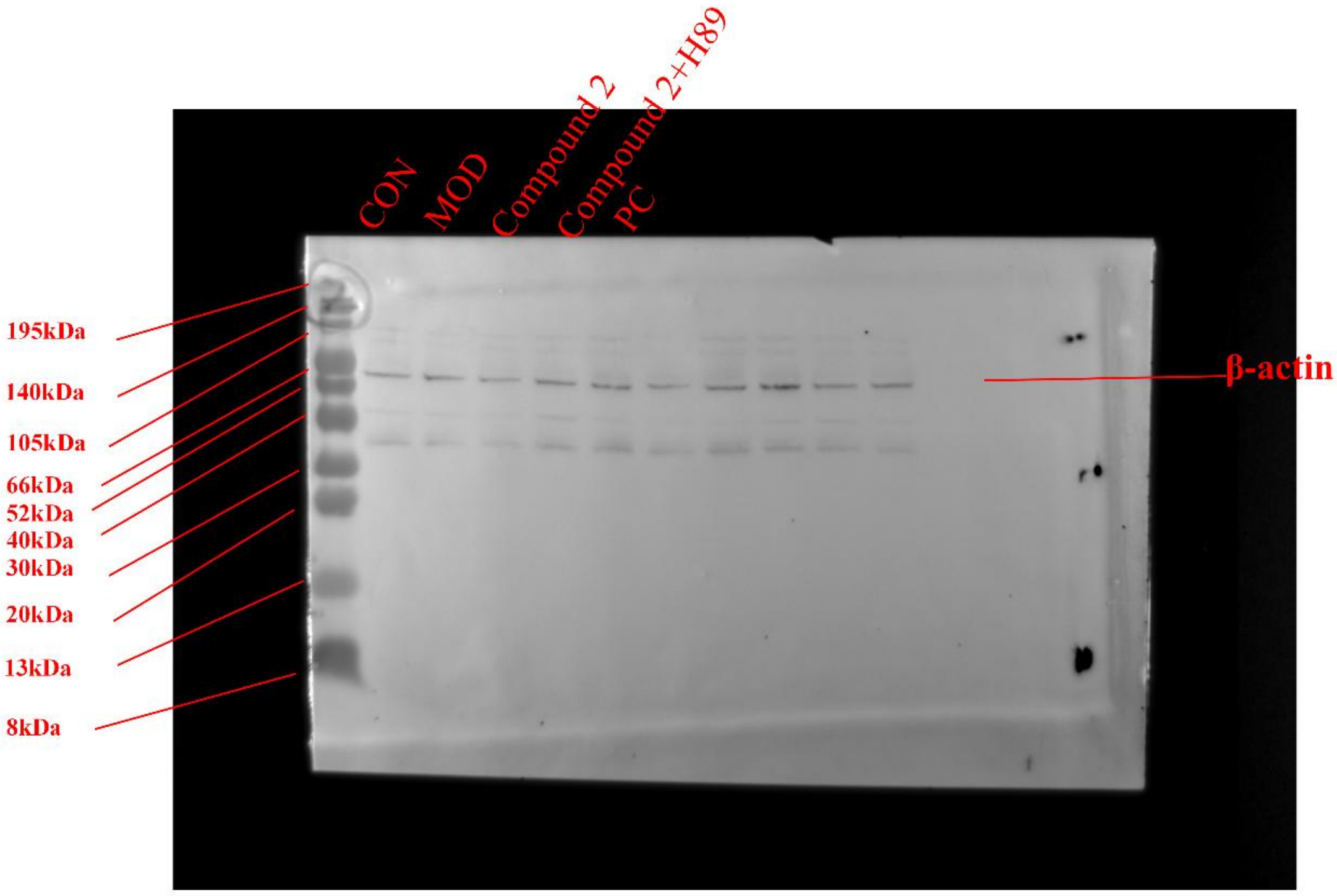

Fig. S11 The western blot images of PKA protein bands (n=3).

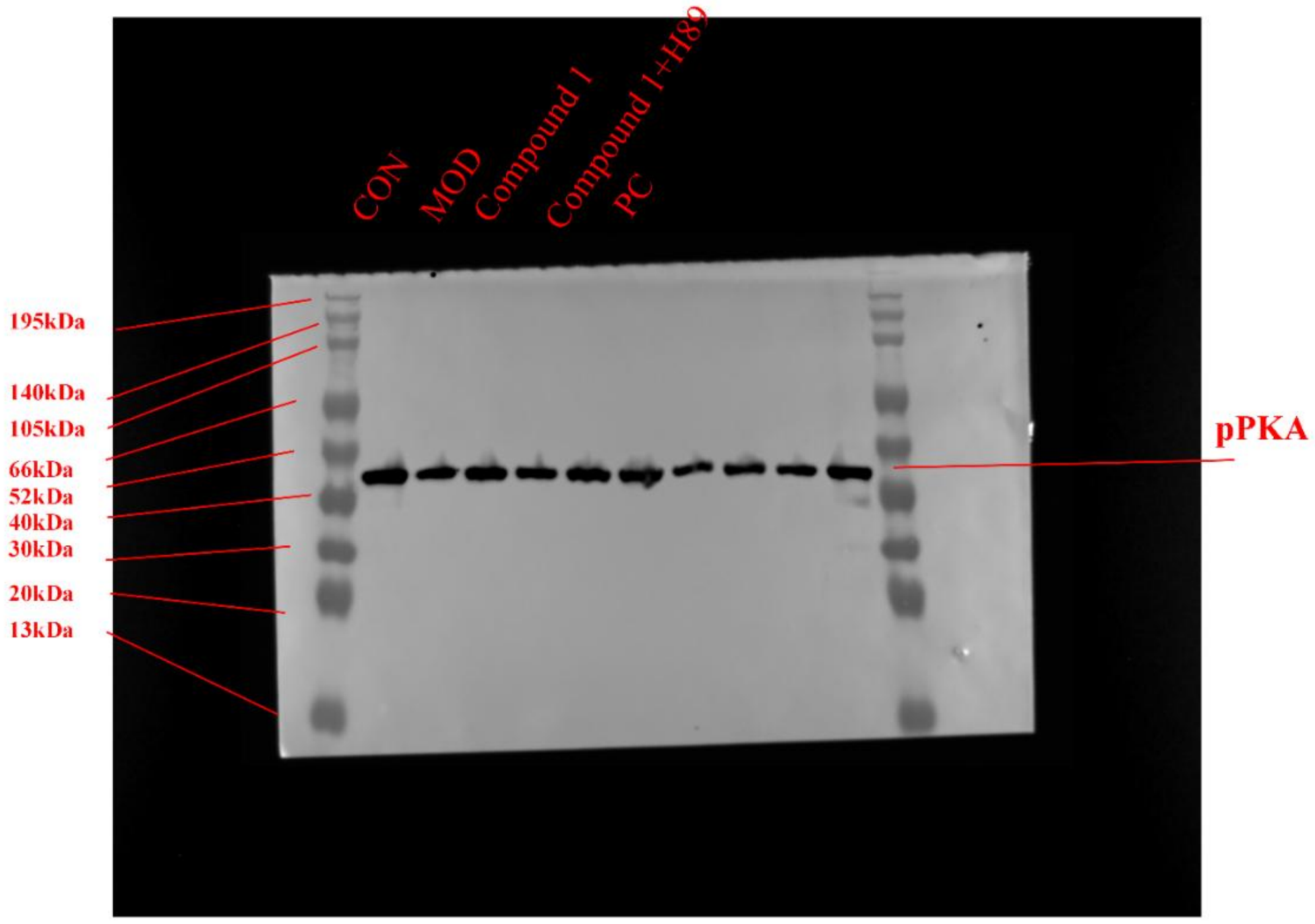
CON
MOD
Compound 1
Compound 1+H89
PC
195kDa
140kDa
105kDa
66kDa
52kDa
40kDa
30kDa
20kDa
13kDa
pPKA

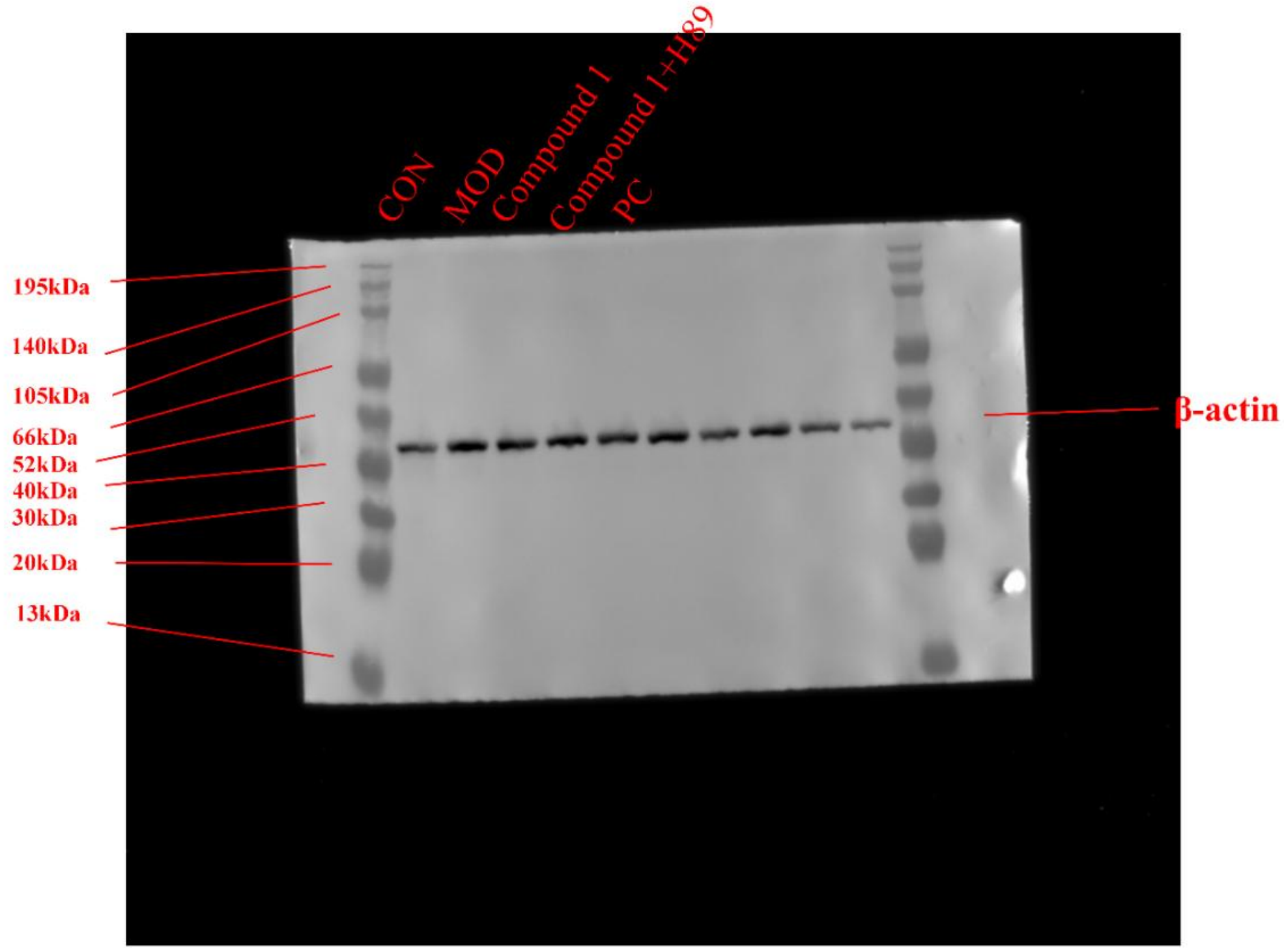
CON
MOD
Compound 1
Compound 1+H89
PC
195kDa
140kDa
105kDa
66kDa
52kDa
40kDa
30kDa
20kDa
13kDa
β-actin

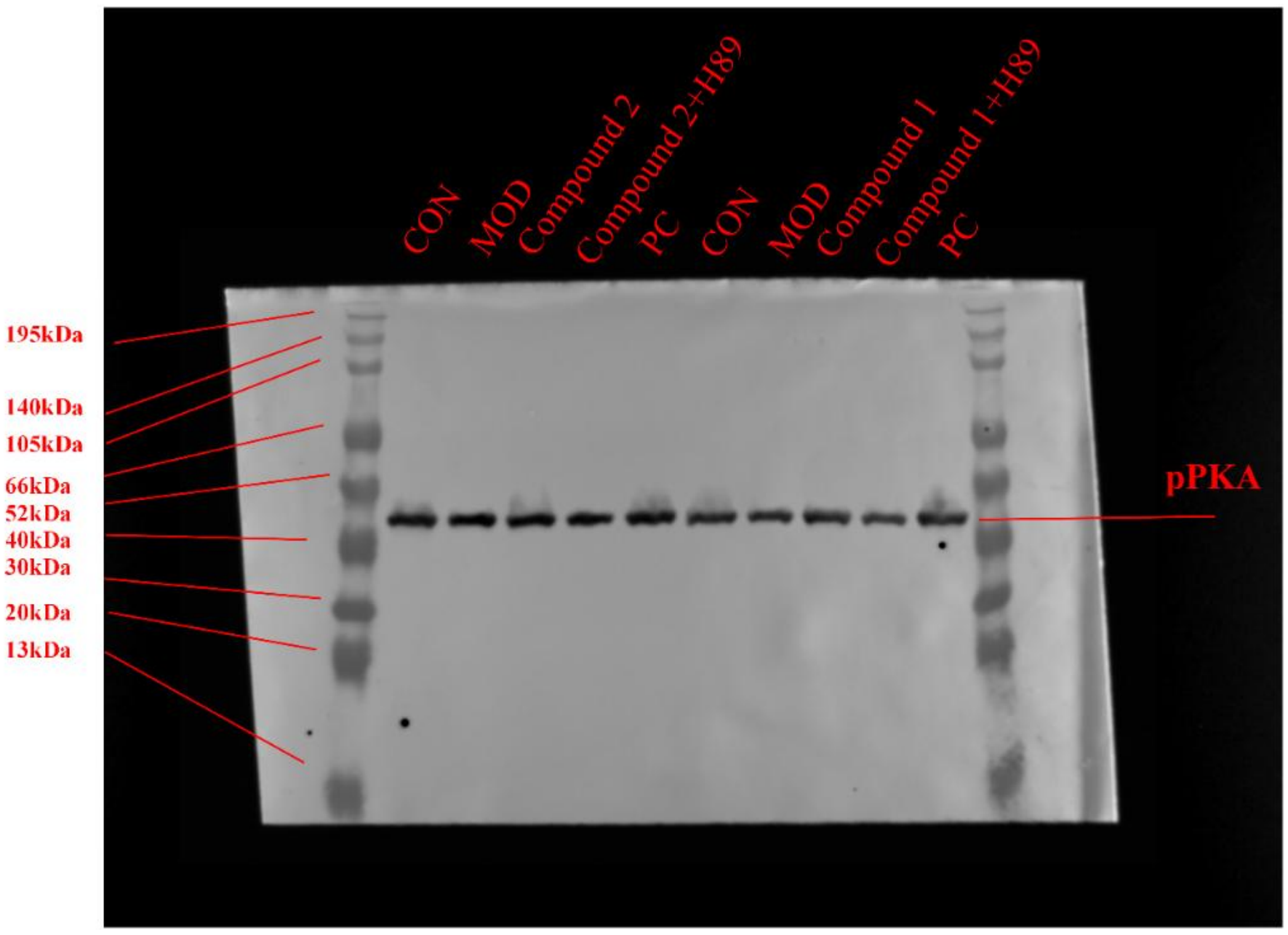
CON
MOD
Compound 2
Compound 2+H89
PC
CON
MOD
Compound 1
Compound 1+H89
PC
195kDa
140kDa
105kDa
66kDa
52kDa
40kDa
30kDa
20kDa
13kDa
pPKA

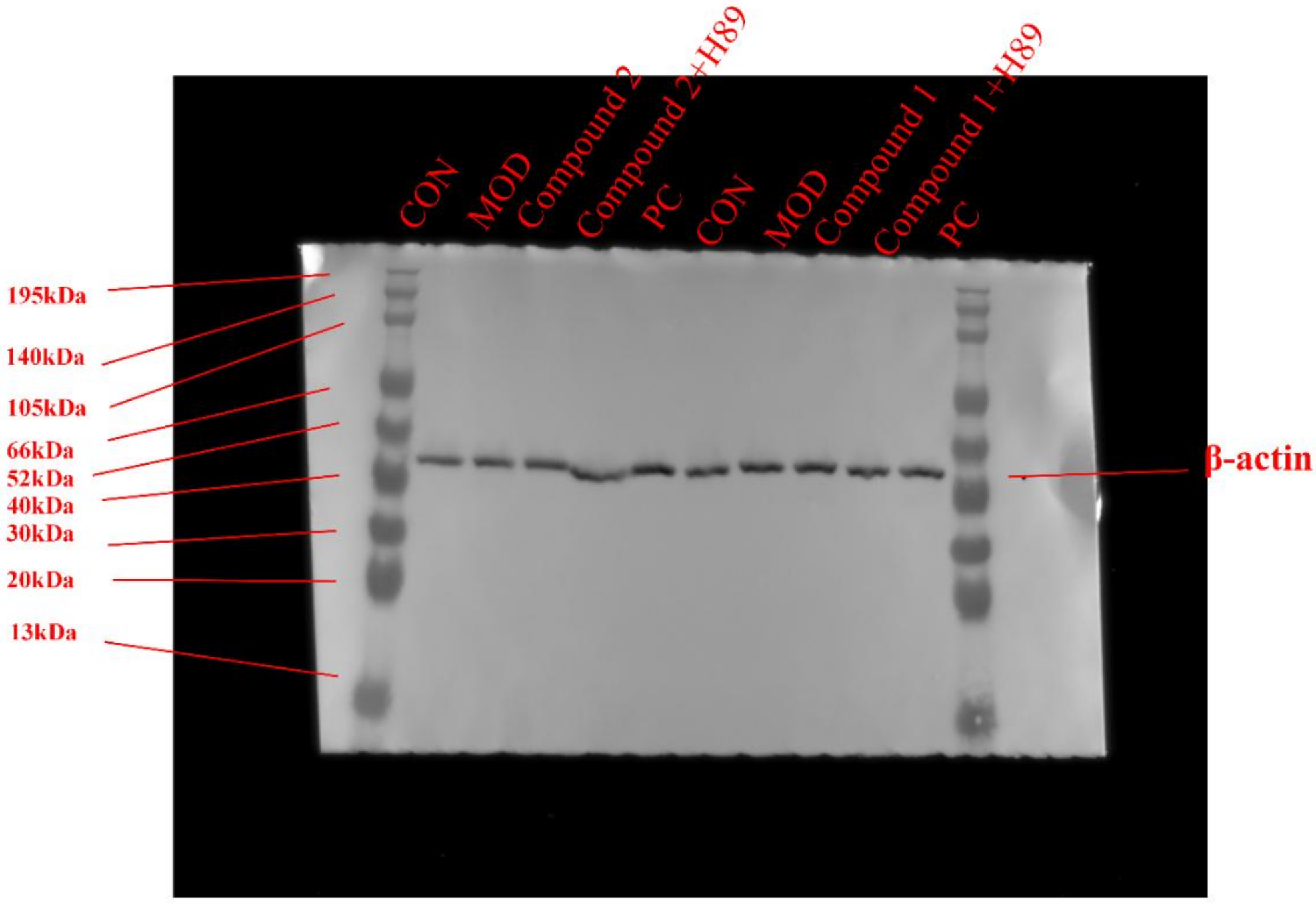
CON
MOD
Compound 2
Compound 2+H89
PC
CON
MOD
Compound 1
Compound 1+H89
PC
195kDa
140kDa
105kDa
66kDa
52kDa
40kDa
30kDa
20kDa
13kDa
β-actin

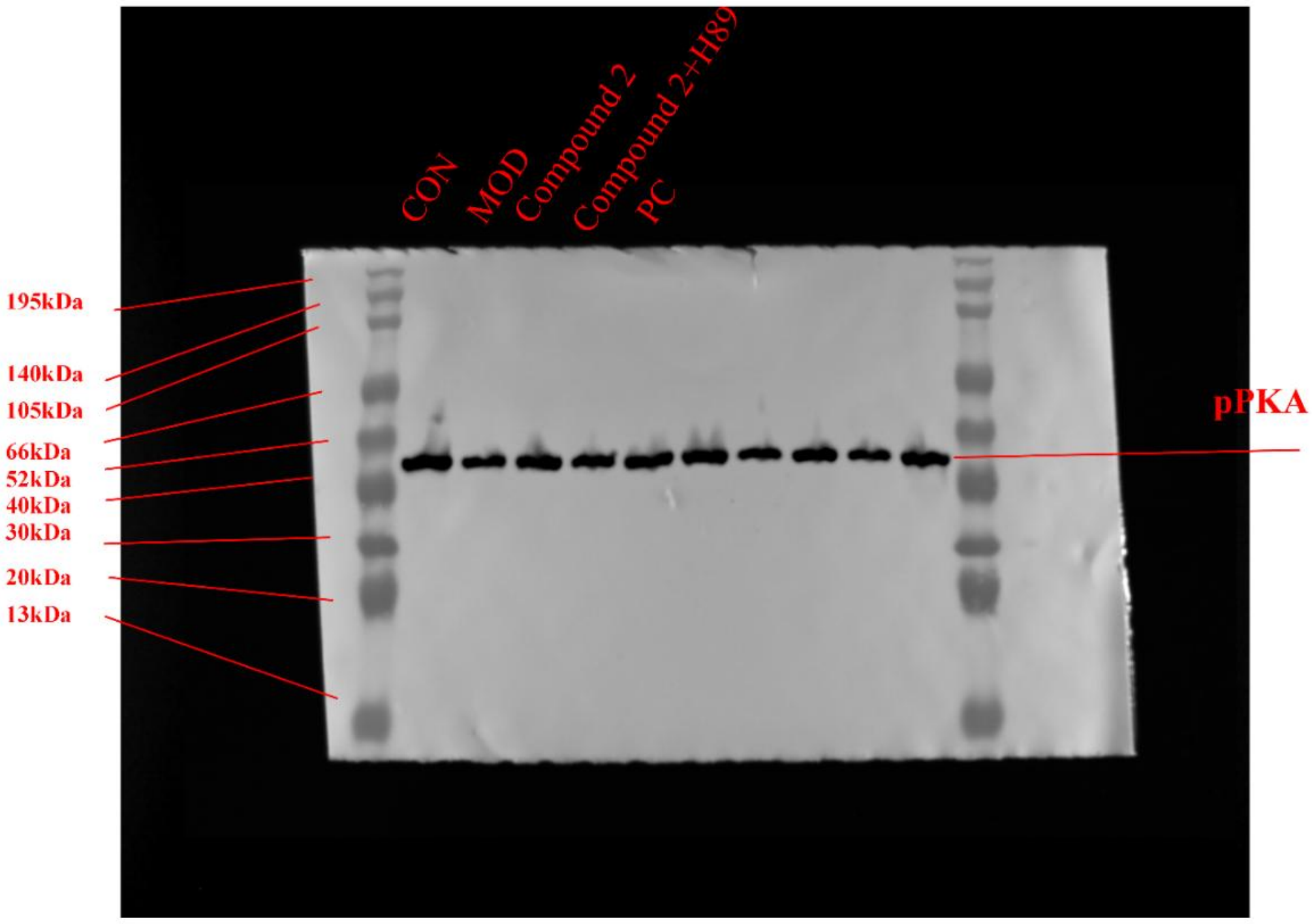
CON
MOD
Compound 2
Compound 2+H89
PC
195kDa
140kDa
105kDa
66kDa
52kDa
40kDa
30kDa
20kDa
13kDa
pPKA

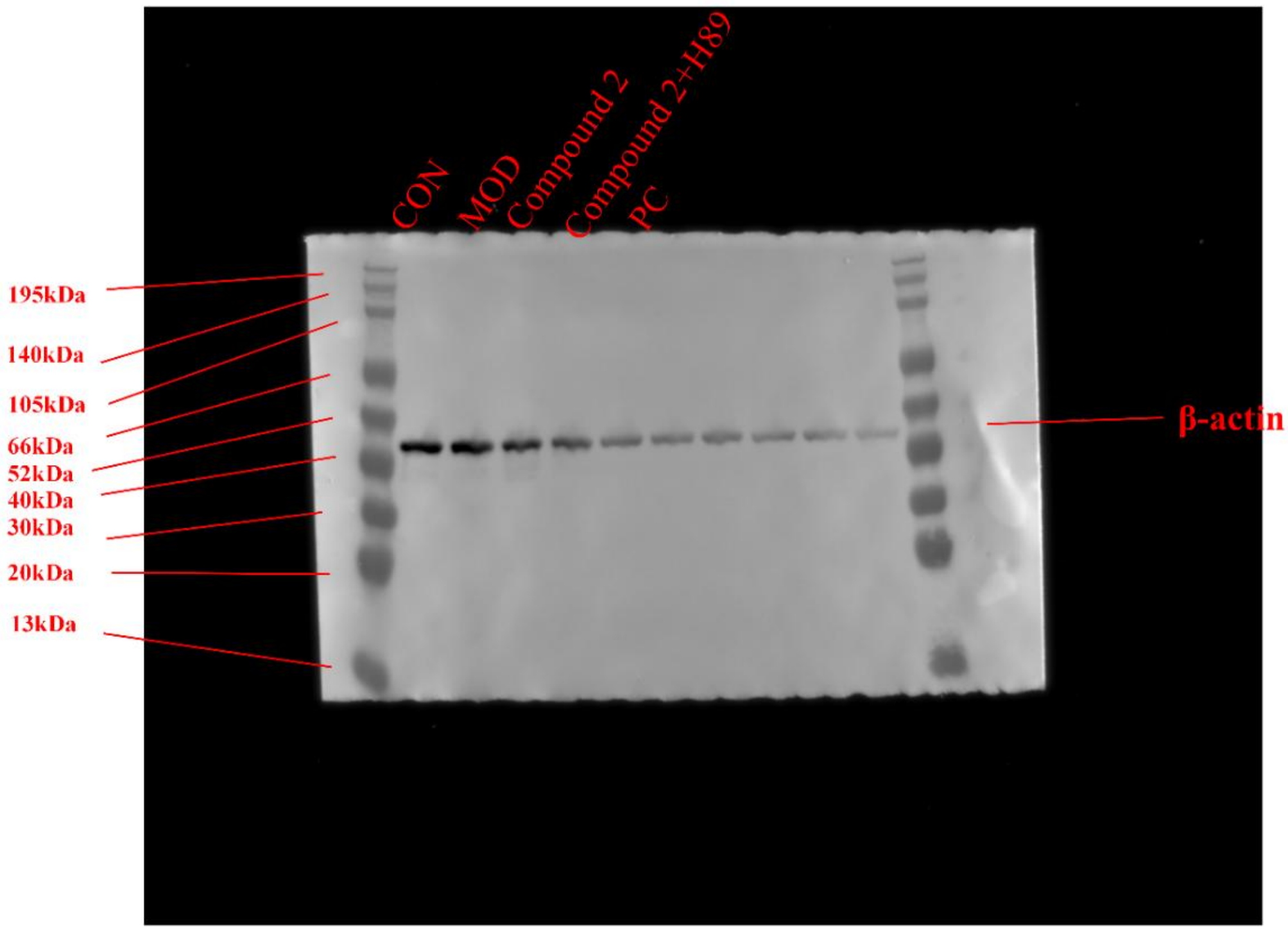
CON
MOD
Compound 2
Compound 2+H89
PC
195kDa
140kDa
105kDa
66kDa
52kDa
40kDa
30kDa
20kDa
13kDa
β-actin

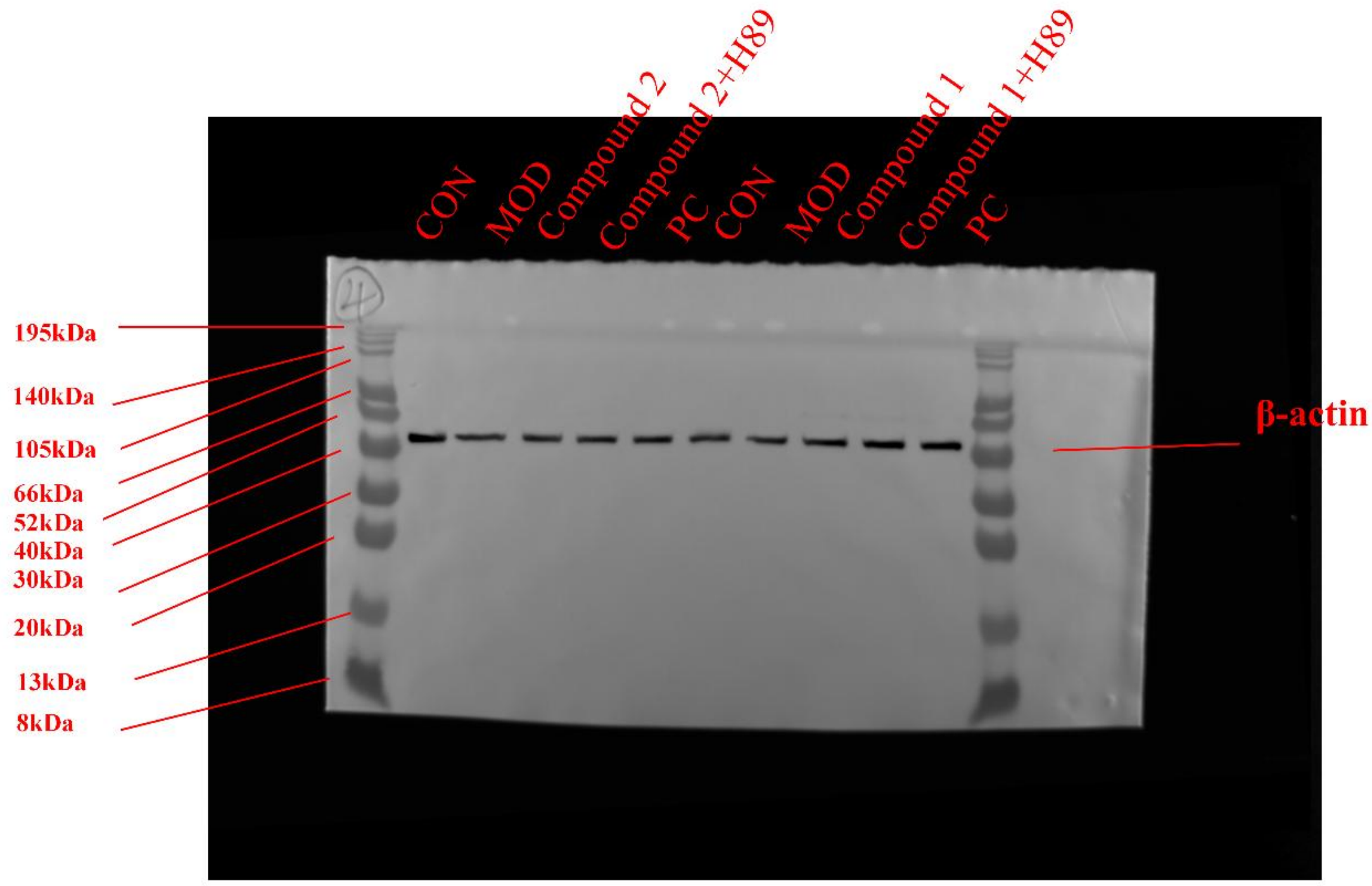

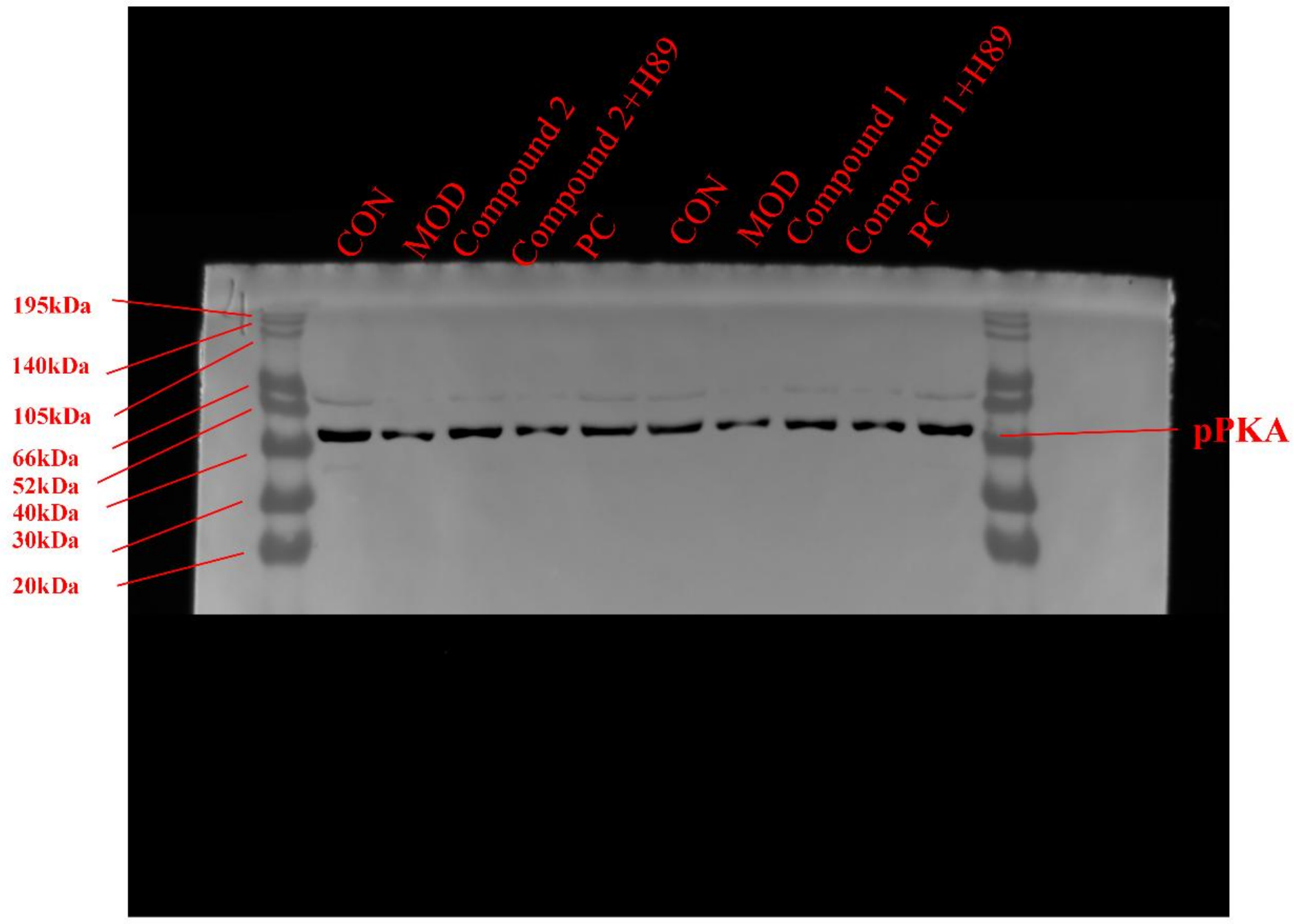

Fig. S12 The western blot images of p-PKA protein bands (n=3).

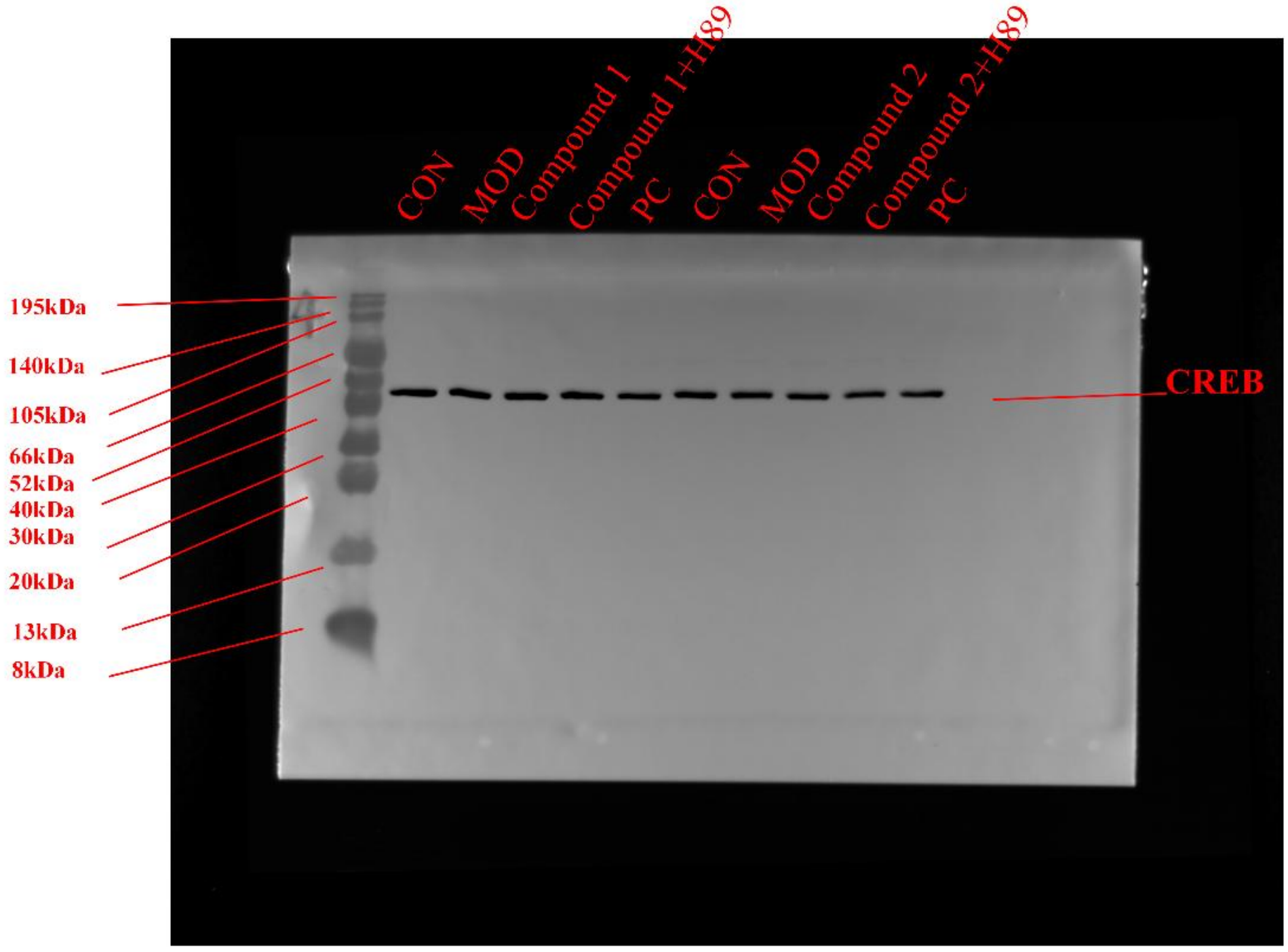
CON
MOD
Compound 1
Compound 1+H89
PC
CON
MOD
Compound 2
Compound 2+H89
PC
195kDa
140kDa
105kDa
66kDa
52kDa
40kDa
30kDa
20kDa
13kDa
8kDa
CREB

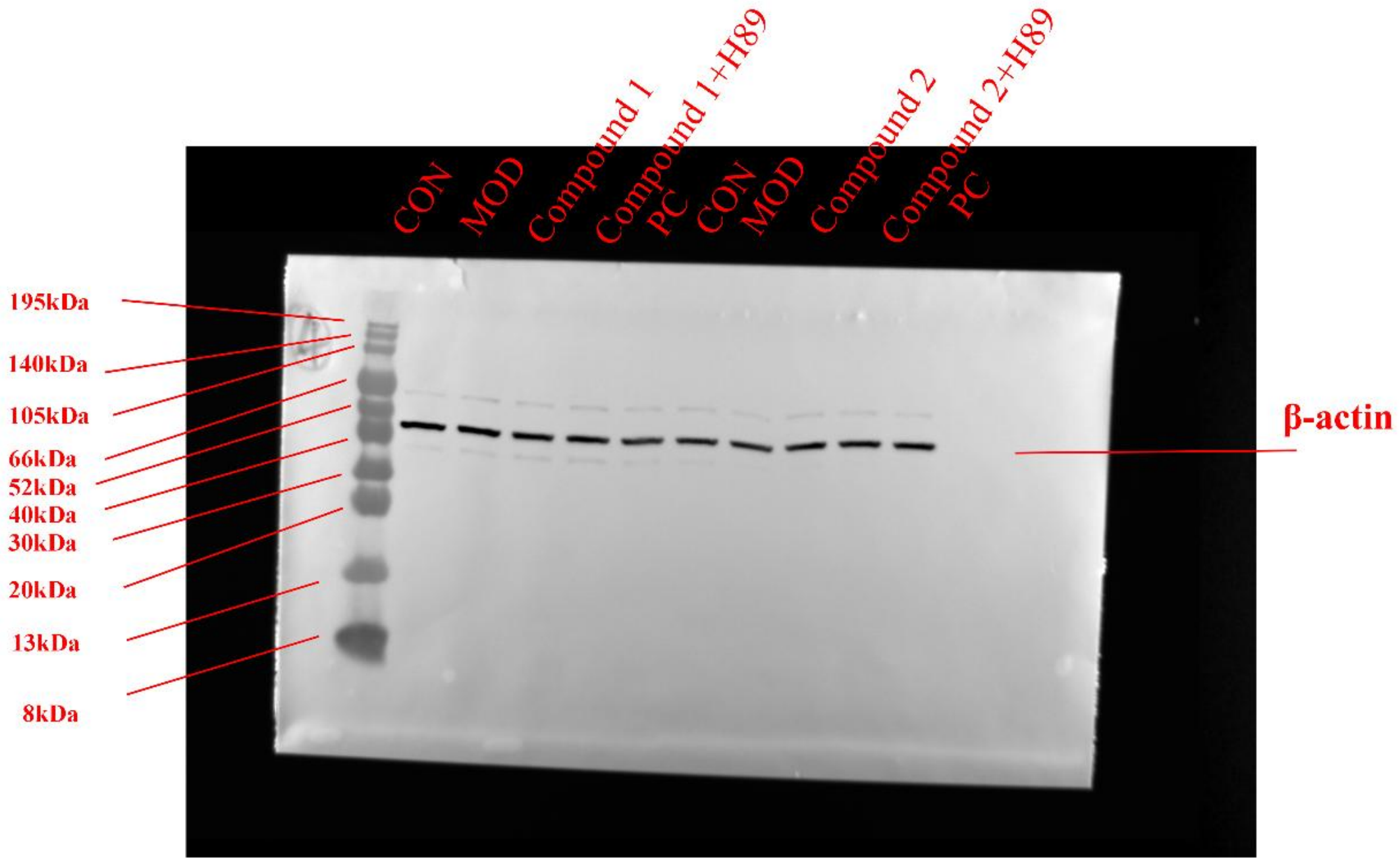
CON
MOD
Compound 1
Compound 1+H89
PC
CON
MOD
Compound 2
Compound 2+H89
PC
195kDa
140kDa
105kDa
66kDa
52kDa
40kDa
30kDa
20kDa
13kDa
8kDa
β-actin

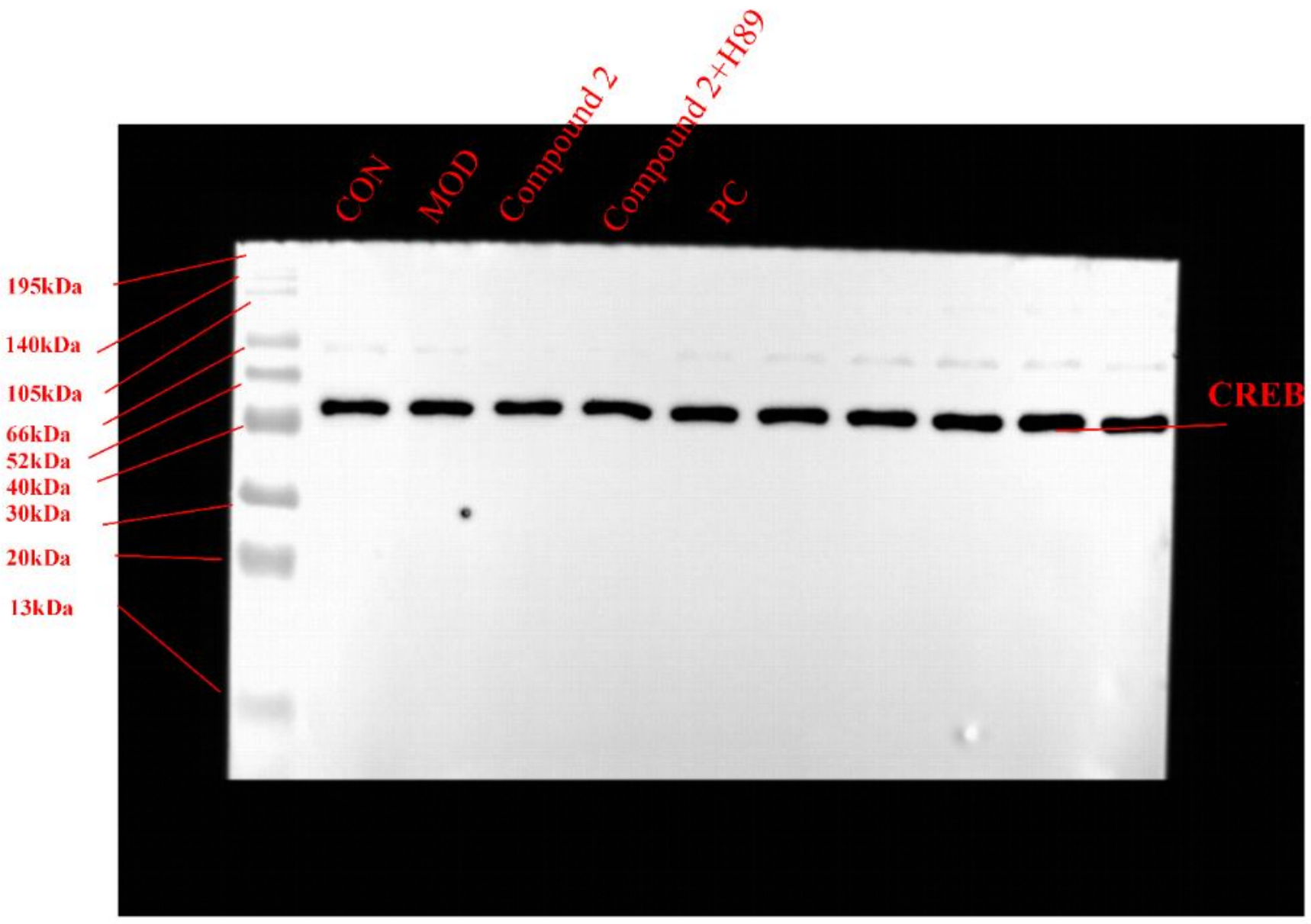
CON
MOD
Compound 2
Compound 2+H89
PC
195kDa
140kDa
105kDa
66kDa
52kDa
40kDa
30kDa
20kDa
13kDa
CREB

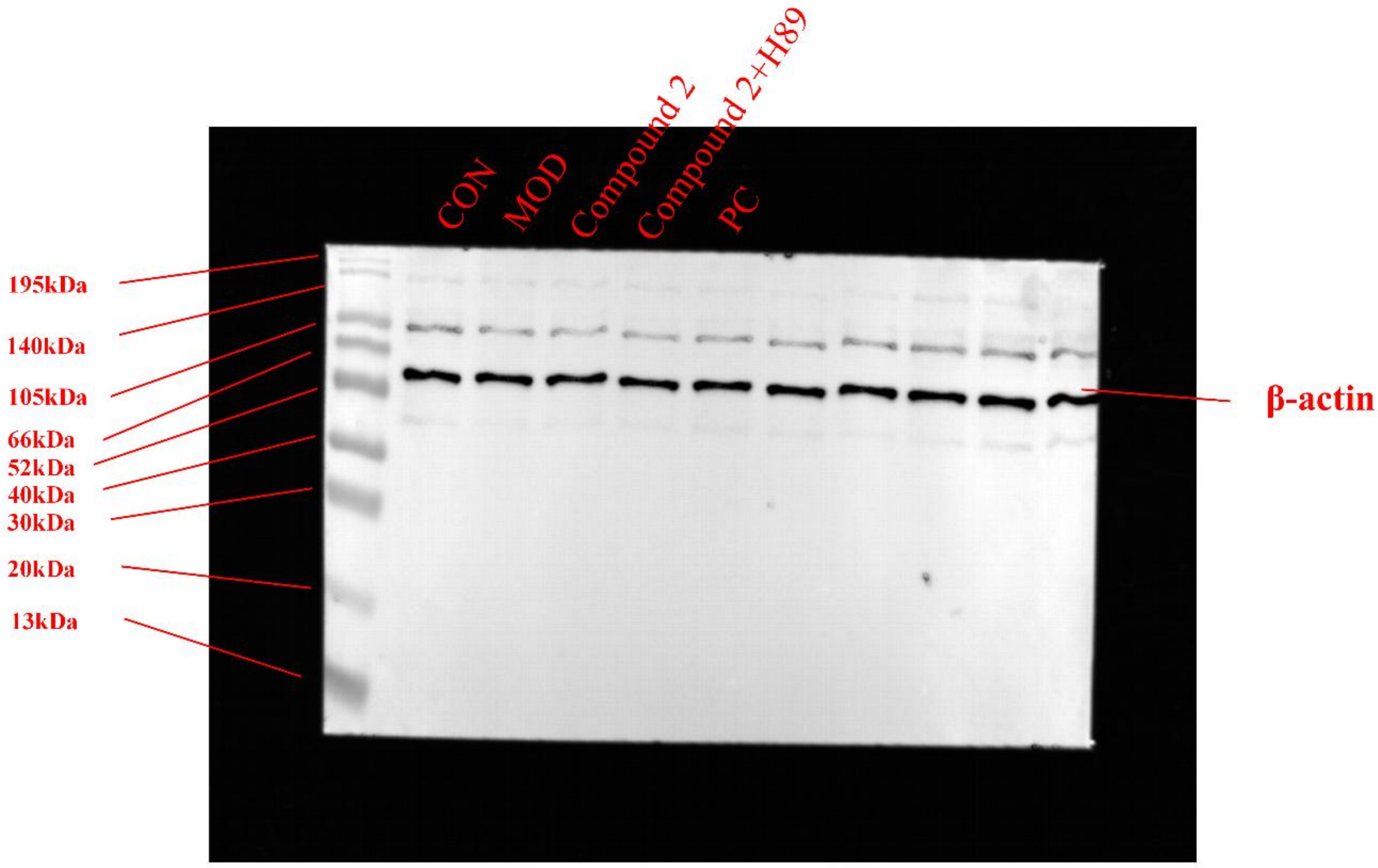
CON
MOD
Compound 2
Compound 2+H89
PC
195kDa
140kDa
105kDa
66kDa
52kDa
40kDa
30kDa
20kDa
13kDa
β-actin

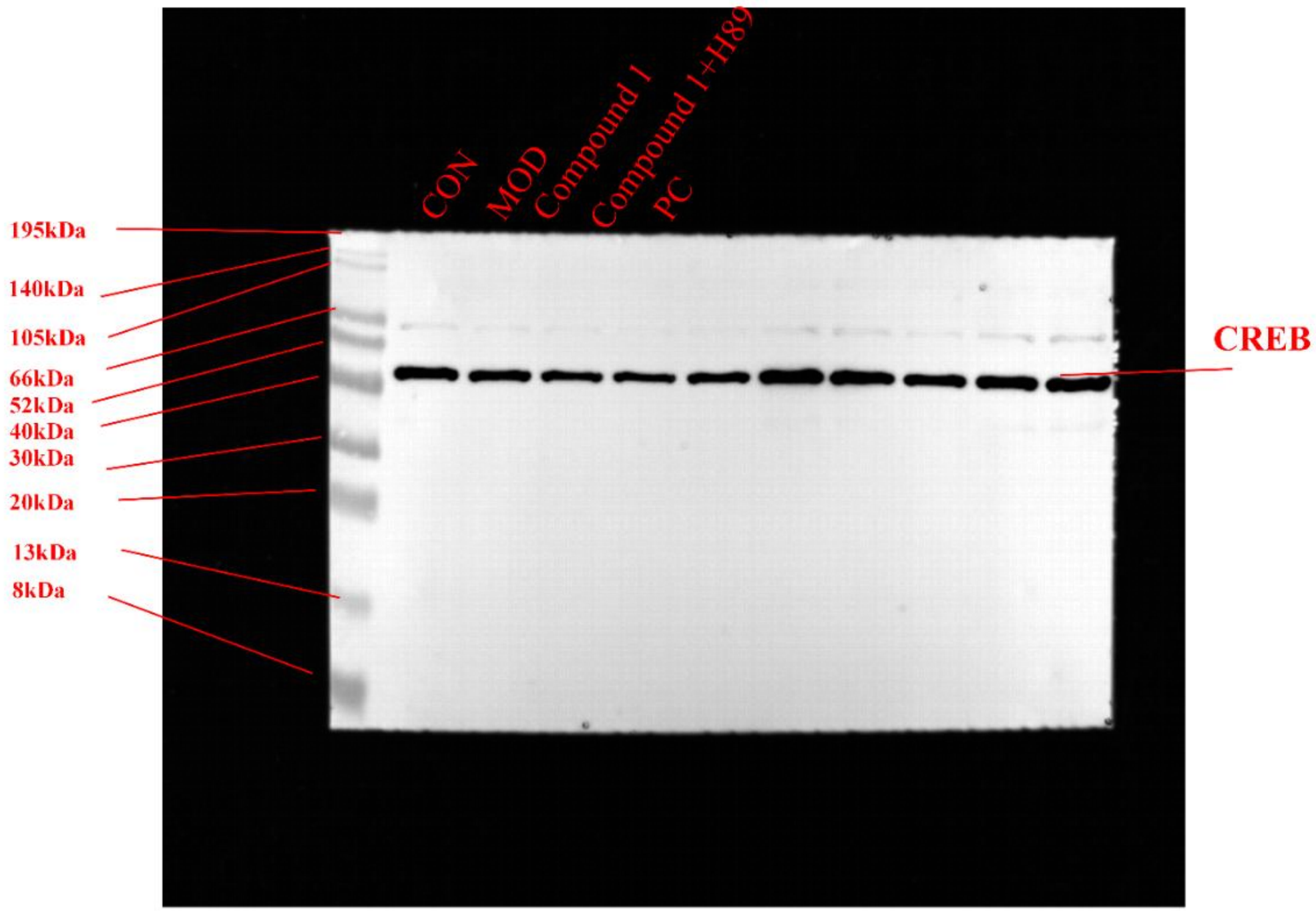
CON
MOD
Compound 1
Compound 1+H89
PC
195kDa
140kDa
105kDa
66kDa
52kDa
40kDa
30kDa
20kDa
13kDa
8kDa
CREB

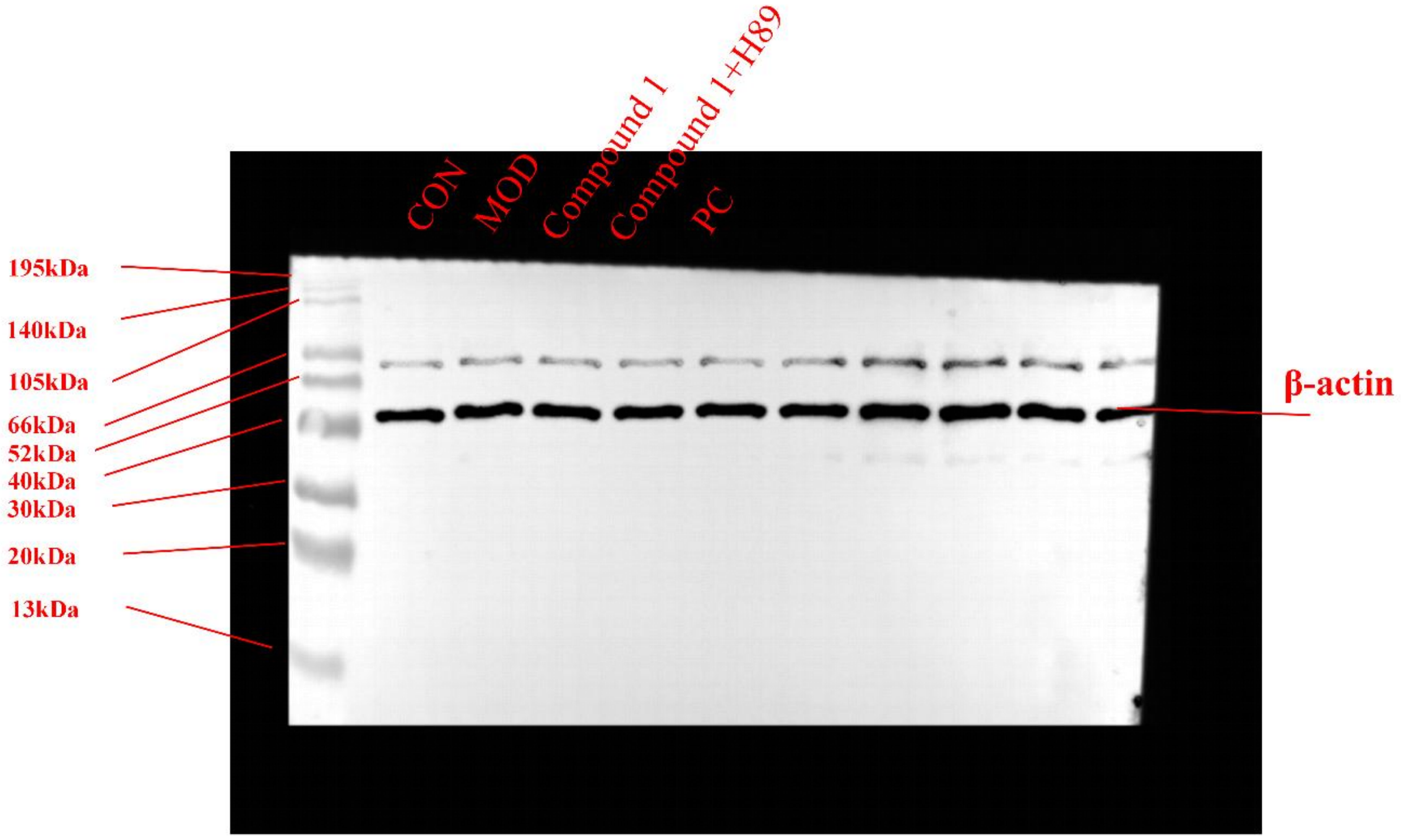
CON
MOD
Compound 1
Compound 1+H89
PC
195kDa
140kDa
105kDa
66kDa
52kDa
40kDa
30kDa
20kDa
13kDa
β-actin

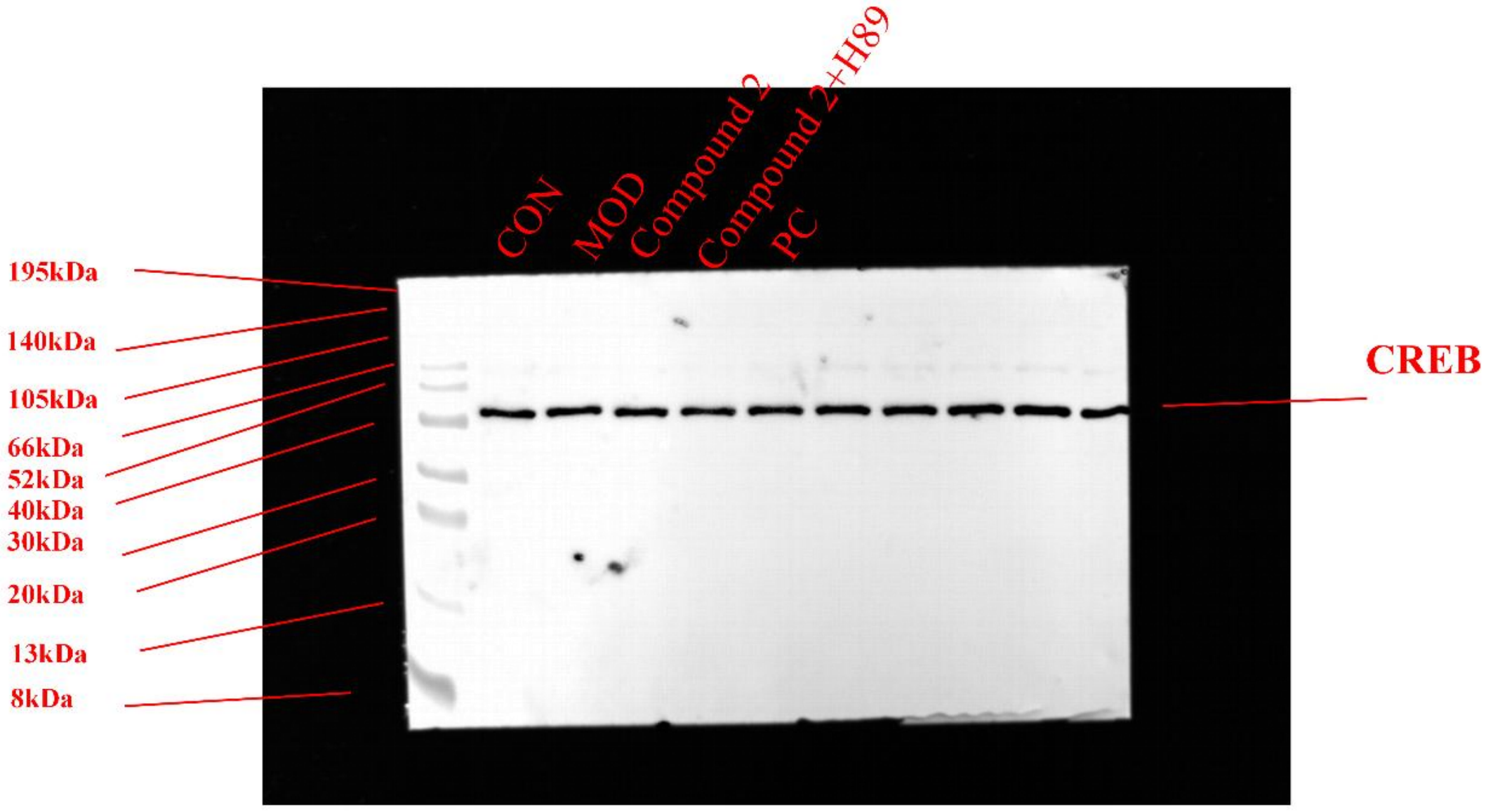
CON
MOD
Compound 2
Compound 2+H89
PC
195kDa
140kDa
105kDa
66kDa
52kDa
40kDa
30kDa
20kDa
13kDa
8kDa
CREB

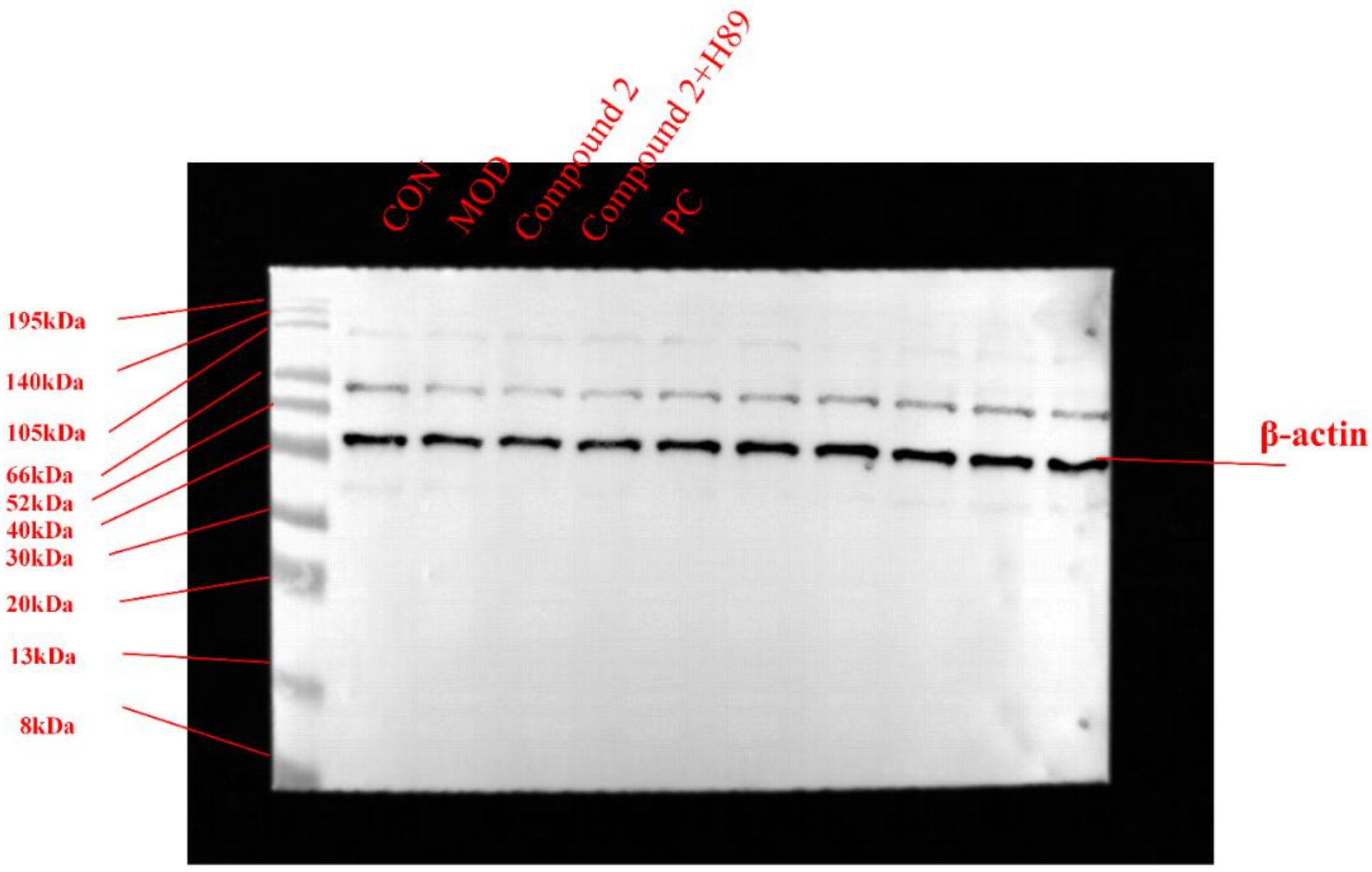
CON
MOD
Compound 2
Compound 2+H89
PC
195kDa
140kDa
105kDa
66kDa
52kDa
40kDa
30kDa
20kDa
13kDa
8kDa
β-actin

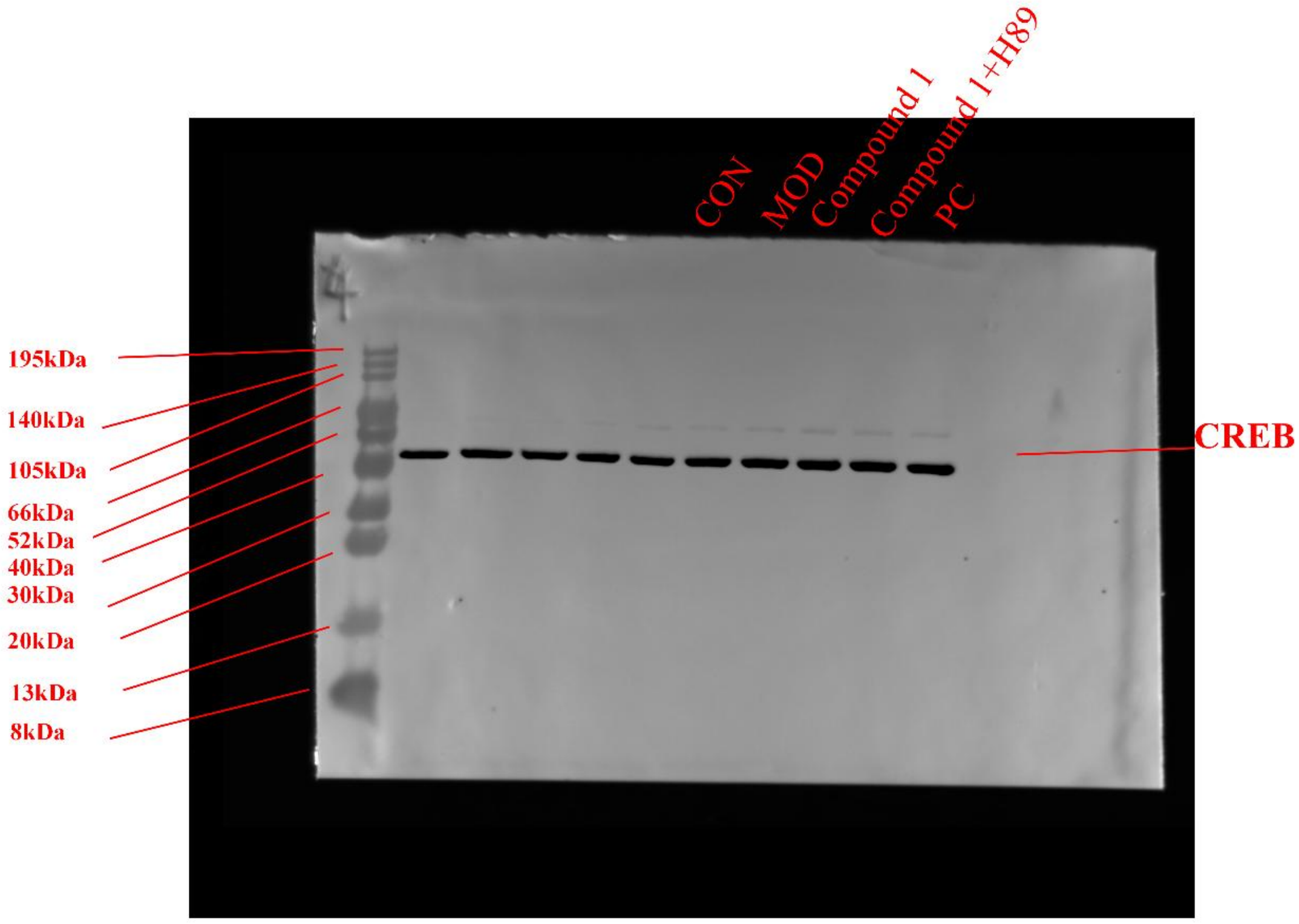

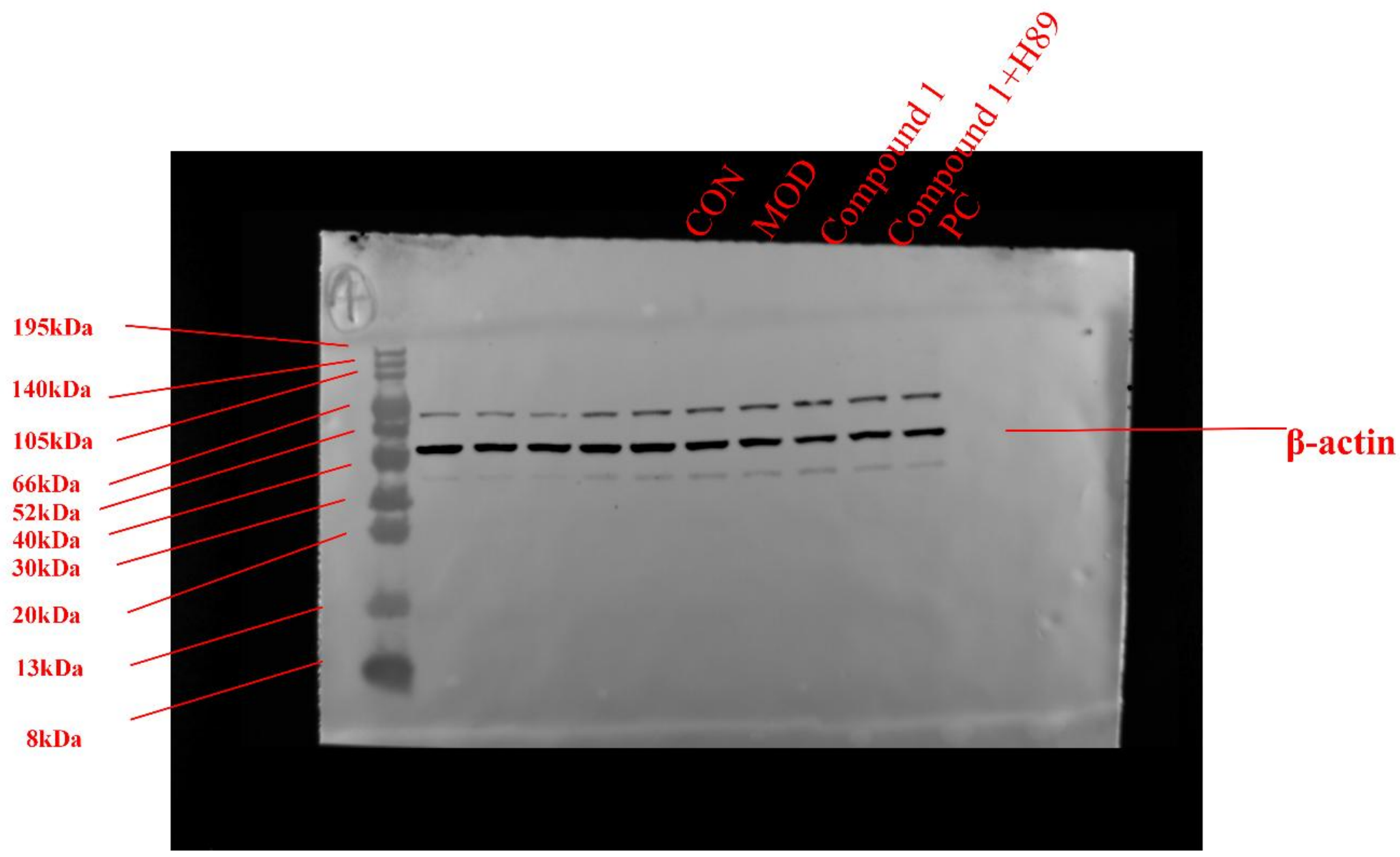

Fig. S13 The western blot images of CREB protein bands (n=3).

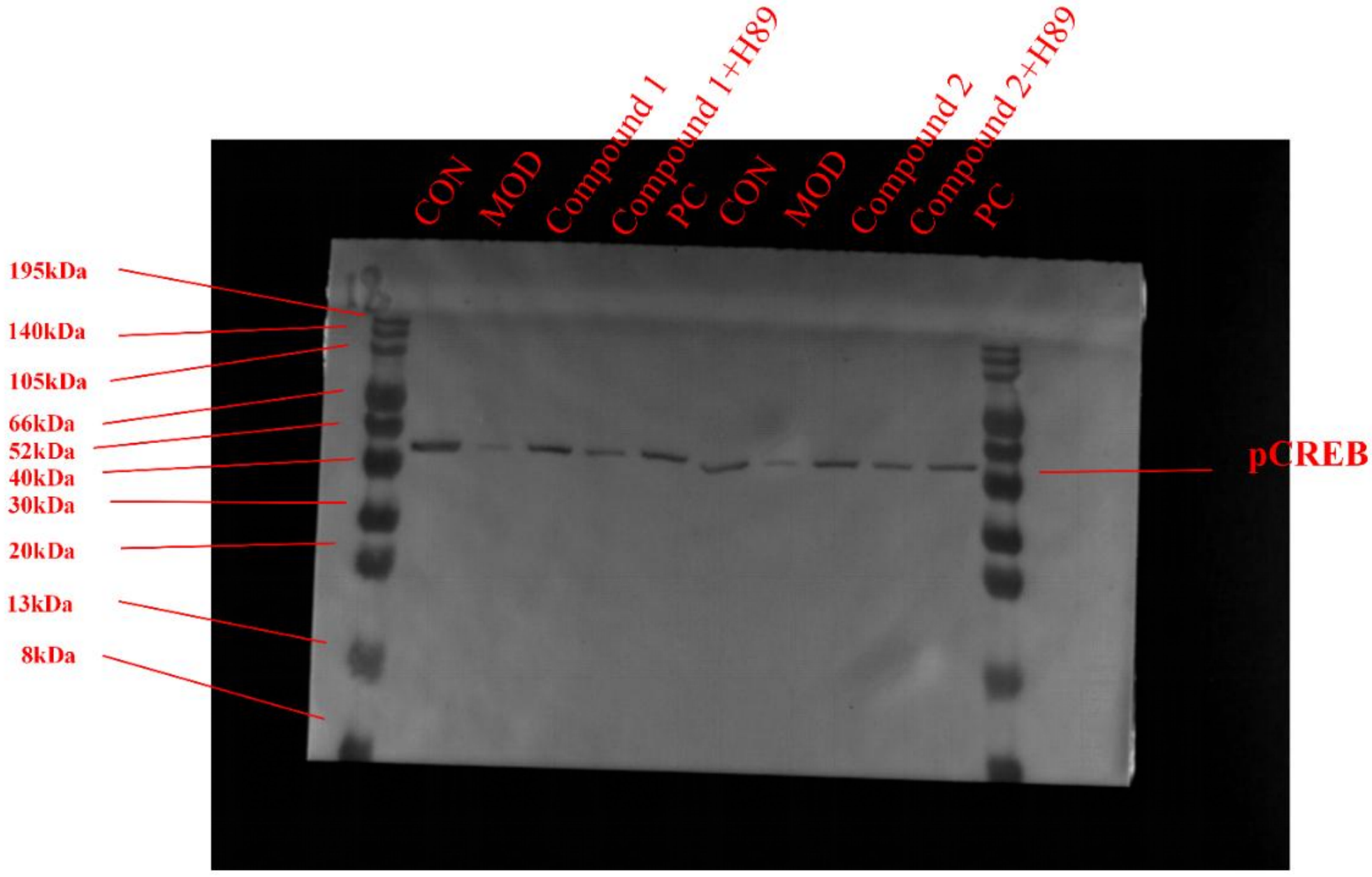
CON
MOD
Compound 1
Compound 1+H89
PC
CON
MOD
Compound 2
Compound 2+H89
PC
195kDa
140kDa
105kDa
66kDa
52kDa
40kDa
30kDa
20kDa
13kDa
8kDa
pCREB

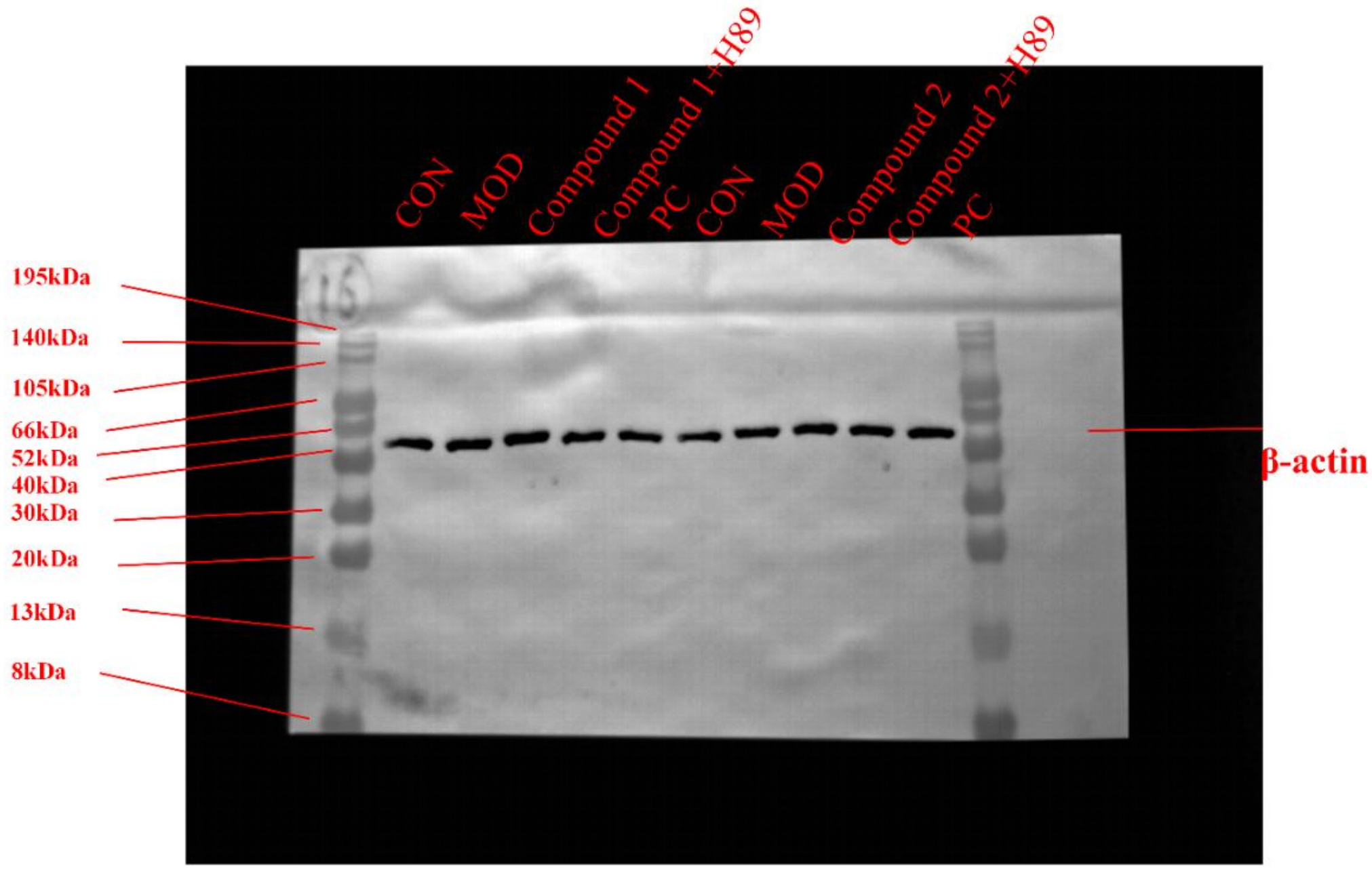
CON
MOD
Compound 1
Compound 1+H89
PC
CON
MOD
Compound 2
Compound 2+H89
PC
195kDa
140kDa
105kDa
66kDa
52kDa
40kDa
30kDa
20kDa
13kDa
8kDa
β-actin

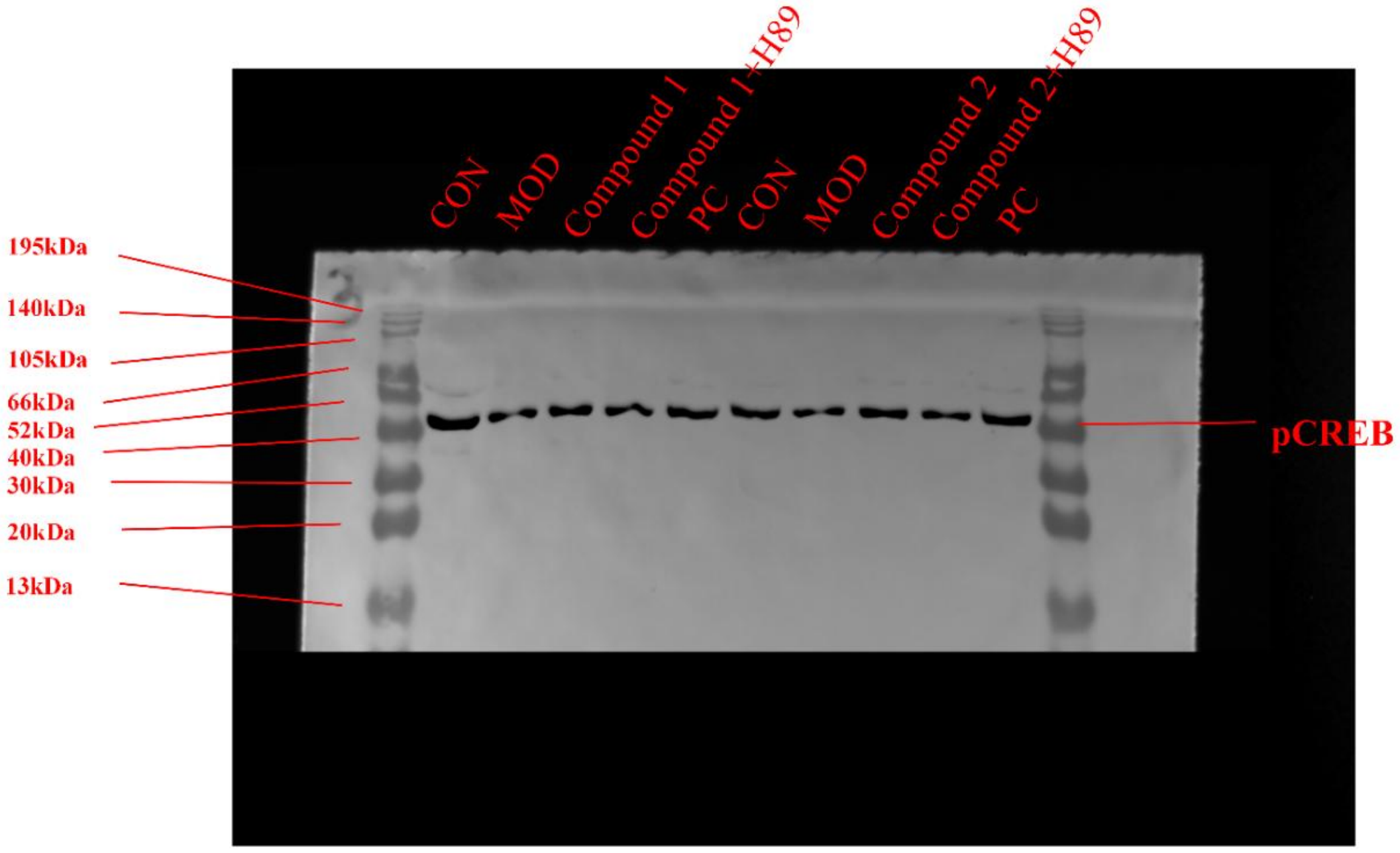
CON
MOD
Compound 1
Compound 1+H89
PC
CON
MOD
Compound 2
Compound 2+H89
PC
195kDa
140kDa
105kDa
66kDa
52kDa
40kDa
30kDa
20kDa
13kDa
pCREB

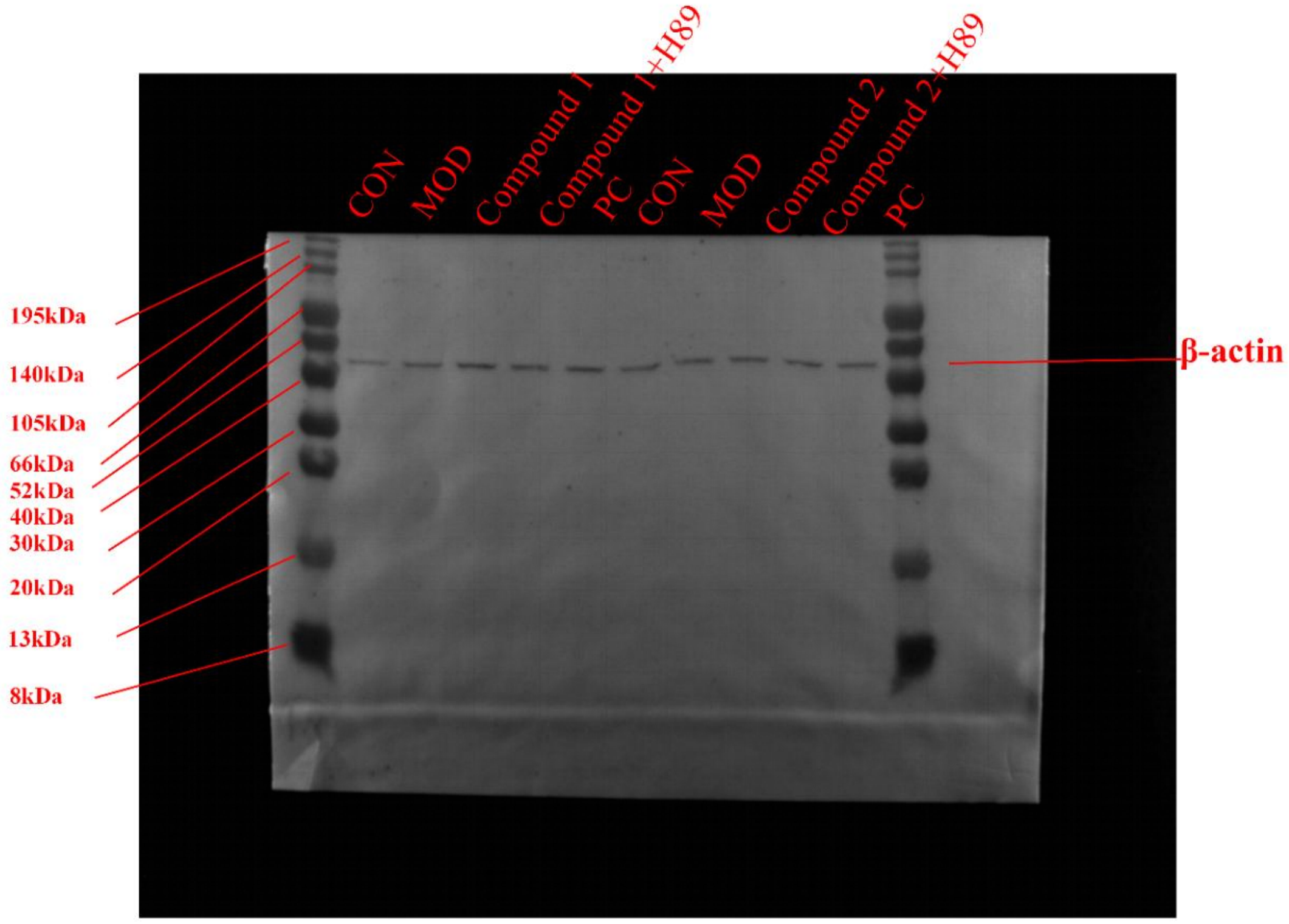
CON
MOD
Compound 1
Compound 1+H89
PC
CON
MOD
Compound 2
Compound 2+H89
PC
195kDa
140kDa
105kDa
66kDa
52kDa
40kDa
30kDa
20kDa
13kDa
8kDa
β-actin

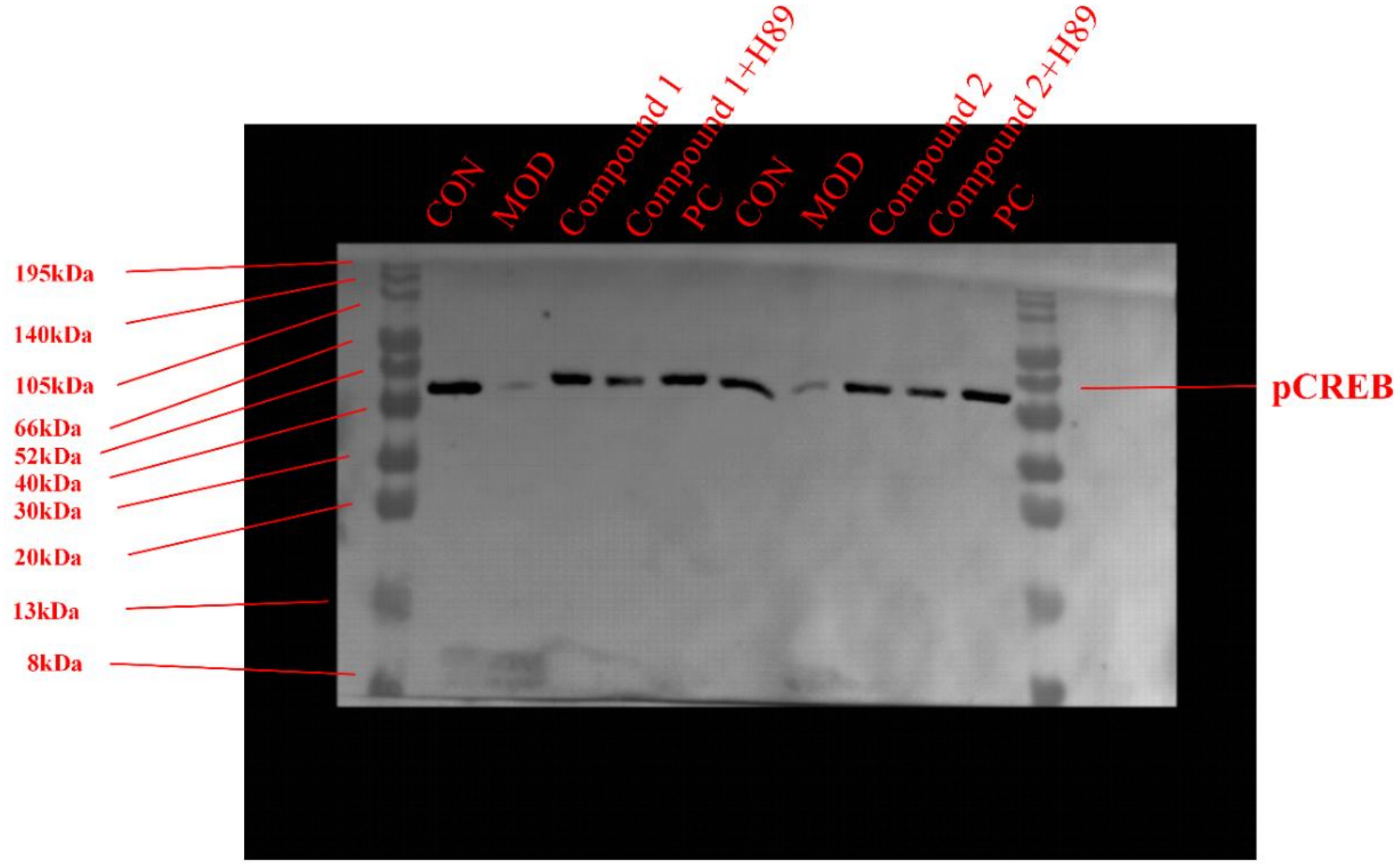

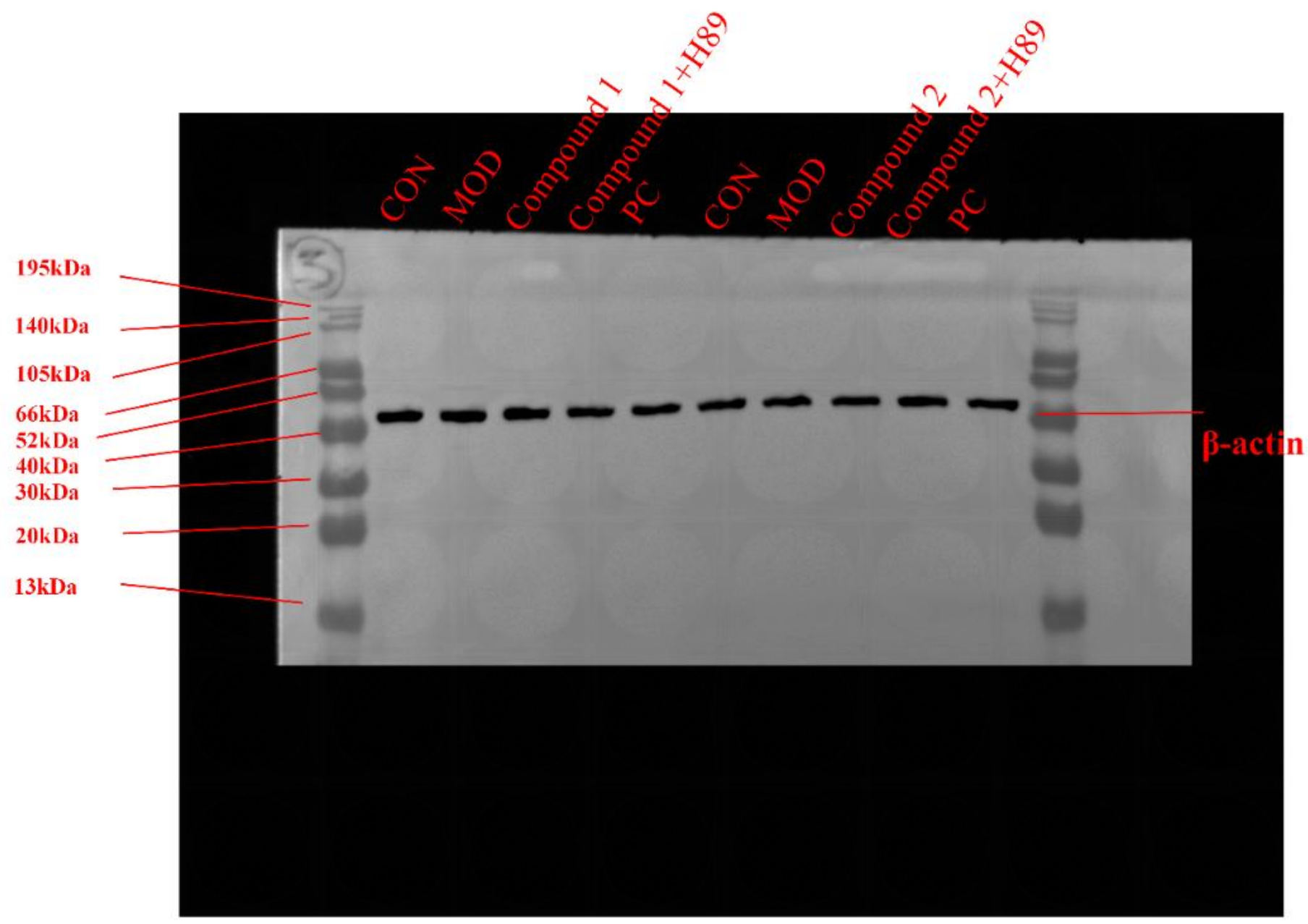

Fig. S14 The western blot images of p-CREB protein (n=3).
